# Characterization and comparation of toxicity between natural realgar and artificially optimized realgar

**DOI:** 10.3389/fphar.2024.1476139

**Published:** 2024-10-28

**Authors:** Lu Luo, Xueying Xin, Qiaochu Wang, Mengjia Wei, Nanxi Huang, Shuangrong Gao, Xuezhu Gu, Raorao Li

**Affiliations:** ^1^ Institute of Chinese Materia Medica, China Academy of Chinese Medical Sciences, Beijing, China; ^2^ Department of Pharmacy, Union Hospital, Tongji Medical College, Huazhong University of Science and Technology, Wuhan, China; ^3^ Department of Biochemistry and Molecular and Cellular Biology, Georgetown University, Washington, DC, United States; ^4^ Departments of Oncology, Georgetown University, Washington, DC, United States

**Keywords:** natural realgar, artificially optimized realgar, identification characterization, arsenic valence, comparison of toxicity

## Abstract

**Objective:**

Realgar possesses important medical properties. This article aims to evaluate realgar and emerging artificially optimized realgar to ensure safe clinical use.

**Methods:**

Multiple techniques were employed to test natural realgar and artificially optimized realgar. Soluble arsenic content in representative samples were measured. Natural realgar and artificially optimized realgar were administered to KM mice via gavage for 28 days, and the extent of liver and kidney tissue damage, arsenic accumulation and form of arsenic were measured.

**Results:**

Natural realgar and artificially optimized realgar can be distinguished by their physical properties or spectral signatures. ICP-MS and EPMA identified different contents of elements between two groups. In simulated gastric and intestinal fluids, only As (III) and As (V) were detected. Toxicity experiments *in vivo* demonstrate that both groups caused minimal liver and kidney damage at a dose of 30 mg·kg^−1^. At a dose of 180 mg·kg^−1^, artificially optimized realgar caused significantly greater liver and kidney damage.

**Conclusion:**

The differences between natural realgar and artificially optimized realgar were successfully distinguished through several methods. *In vitro* experiments showed that As is the main component exerting their medicinal effects. *In vivo* toxicity tests demonstrated that at higher dose, artificially optimized realgar exhibited significantly higher toxicity, suggesting that natural and artificially optimized realgar have different toxic properties.

## 1 Introduction

Mineral medicines have a long history in China. Records of their use date back to 1600 BCE to 475 BCE (Zhang et al., 2024; Chen et al., 2023). One of these mineral medicines is realgar (Luo et al., 2023). Realgar is a traditional Chinese medicine derived from the realgar sulfide mineral and is effective in treating diseases demonstrated by extensive pharmacological studies (Wu et al., 2023). Earlier study identified the role of realgar on antibacterial. For example, realgar has demonstrated efficacy in killing *Escherichia coli* and *Staphylococcus aureus* (Cao et al., 1986). Studies have shown that using realgar extract to treat mice inoculated with *S. aureus* and *Pseudomonas aeruginosa* significantly reduces mortality rates (Du, 2008). Some studies suggest that realgar enhances the defense capabilities of *Caenorhabditis elegans* against *Enterococcus faecalis* by inducing immune responses and regulating the p38 MAPK and DAF-16 pathways (Ma et al., 2021). Other studies expanded the role of realgar to anti-virus. For example, antiviral activity of nano-realgar against HSV-2-infected cells under different administration methods was investigated (Wang et al., 2019). The result showed that nano-realgar’s preventive administration had superior anti-HSV-2 activity compared to therapeutic and direct inactivation administration.

Recent studies further expand the use of realgar. Realgar is particularly used in the clinical treatment of acute promyelocytic leukemia (Li et al., 2017). Anti-myeloma activity of nano-realgar and ATO (arsenic trioxide) were compared and both induce apoptosis by causing DNA fragmentation and cleavage of anti-apoptotic proteins (Cholujova et al., 2017). However, nano-realgar exhibited superior activity compared to ATO. This study provides new guidance for medication and prognosis in clinical treatment of myeloma patients. Other study suggests that the inorganic arsenic in realgar exerts anti-cancer effects by inducing tumor cell apoptosis, differentiation, inhibiting cancer cell proliferation, and affecting tumor angiogenesis (Wu et al., 2011). Studies showed that realgar inhibits the survival and metastasis of breast cancer cells by regulating the cell cycle, causing G2/M phase arrest, activating pro-apoptotic protein expression, inducing autophagy, and increasing ROS accumulation within cells (Zhao et al., 2019).

Realgar consists of bound arsenic and free arsenic. Arsenic includes arsenic combined with sulfur, which is poorly soluble in water and less absorbable, generally considered less toxic, while arsenic combined with oxygen, which is slightly soluble in water and more toxic (Zhang et al., 2015). Modern research indicates that soluble arsenic is the main component exerting realgar’s medicinal effects (Chen et al., 2022; Liu et al., 2023). However, improper use of realgar can cause toxicity, including hepatotoxicity, nephrotoxicity, neurotoxicity, and genotoxicity. For example, administering different doses of realgar to mice by gavage for 3 weeks caused liver cell damage (Zhang et al., 2014). Oxidative stress and inflammatory responses were identified as the main biological mechanisms leading to these lesions (Zhu et al., 2013). Other studies showed that after 8 weeks of realgar gavage, the arsenic content in the liver of the treated mice significantly increased compared to the control group, showing a dose-dependent trend (Li et al., 2021). Histopathological examination of liver tissues in the treated group revealed vacuolization, expanded cell gaps, and inflammatory cell infiltration.

In addition to arsenic, realgar contains various inorganic elements, which are crucial for their medicinal effects. However, similar to arsenic, heavy metals such as lead (Pb), cadmium (Cd), mercury (Hg), and copper (Cu) present potential health risks and monitoring these elements in realgar is essential for clinical use. Measuring 30 trace elements in realgar and common realgar-containing Chinese patent medicines showed that the Hg content in realgar exceeded the standard limit, with principal component analysis indicating that Chromium (Cr), Manganese (Mn) and Cd could be characteristic elements of these medicines (Li et al., 2017). Others established characteristic profiles of 12 inorganic elements in processed realgar products and conducted *in vitro* anti-colon cancer experiments, finding that the anti-colon cancer effect of realgar is closely related to 11 elements such as Arsenic (As), Strontium (Sr) and Mn (Wang et al., 2023).

Natural realgar is extracted from realgar deposits, which primarily form due to tectonic activity. The Shimen realgar mine in Hunan is the largest mineral resource of high-quality realgar in Asia. It is also the only government-approved production site for medicinal realgar in China. However, years of mining has severely polluted the surrounding rivers and soil with arsenic and other metals due to historical unregulated mining and the careless disposal of mining waste (Liu, 2014). As a result, mining remains banned. Therefore, due to the non-renewable nature of this mineral and national policies restricting the exploitation of mineral resources, identifying the source of natural realgar has become more important in recent years. Many methods have been developed to identify the realgar. Traditionally, microscopic identification is used to identify morphology and optical characteristics. Recently, techniques such as Raman spectroscopy, XRD fingerprinting, and X-ray diffraction analysis have been employed to analyze different types of realgar (Cao et al., 2012; Tian et al., 1998; Zhang et al., 2011; Liao et al., 2013; Luo et al., 2023; Ming et al., 2018). For example, XRD and Raman spectroscopy can be used to identify the crystal structure of medicinal realgar in China, confirming that the realgar sourced from the Shimen mine in Hunan has an α-As_4_S_4_ crystal structure (Zhang et al., 2011).

With the growing recognition of realgar’s medicinal value, the supply-demand conflict has intensified, leading to the appearance of optimized artificial realgar in the medicinal market. This raises the question: is there a quality difference between natural realgar and artificial optimized realgar? There are few studies on this topic. This study found differences between natural and artificial optimized realgar in terms of morphology, and after grinding, they could be distinguished by observing impurity content, XRD phase analysis, Raman spectral peak positions, and differences in inorganic elements. *In vitro* extraction experiments showed that the soluble arsenic forms in natural and synthetic realgar were both As (III) and As (V) in three dissolution media that mimicking human gastrointestinal fluids. Toxicity experiments indicated that at a dose of 30 mg·kg^−1^, both natural and artificially optimized realgar caused minimal liver and kidney damage, with no significant difference in damage degree. However, at a dose of 180 mg·kg^−1^, artificially optimized realgar caused significantly greater liver and kidney damage than natural realgar. This study used various methods to analyze the components of natural and artificially optimized realgar, selecting representative samples for comparative liver and kidney toxicity studies, providing experimental data for their identification, quality evaluation, and clinical safety.

## 2 Method

### 2.1 Realgar samples sources

Thirty samples were collected from various sources for testing. Among these, 24 were medicinal material samples and 6 were realgar processed products (commercially available drinks). The detailed information is in Supplementary Table S1.

### 2.2 Realgar sample characteristics

Sample characteristics were recorded by a digital camera (A7CR, Sony) or polarized optical microscope (AxioScope.A1, ZEISS).

### 2.3 Spectral characteristics of natural and artificially optimized realgar

A suitable amount of each batch of samples was ground into a fine, uniform powder using an agate mortar, and prepared for testing. A small quantity of the powdered sample was spread evenly in the groove of a sample plate using a glass plate. X-ray diffraction spectra for each sample were collected according to the manual, with data processing conducted using MDI Jade 6.5 software. The X-ray diffraction instrument used was a Bruker D8 Advance (Germany), with a Cu target. It operated at a voltage of 40 kV and a current of 40 mA. The scanning range was 10°–70°, with a scanning speed of 8°·min^−1^ and a step size of 0.02°. The PDF card database used was the PDF2 issued by the International Centre for Diffraction Data (ICDD).

### 2.4 Raman spectra of natural and artificially optimized realgar

A suitable number of samples were ground into a fine powder using an agate mortar. For each sample, 2 g of the powder was placed in a sample cup and gently shaken to create a smooth surface. The probe was then lightly touched to the surface of the powder to collect the spectrum. Three spectra were collected for each batch of samples and averaged to obtain the analytical spectrum for each sample. The Raman spectra were obtained using a laser micro-Raman spectrometer (DXR2xi, Thermo Scientific, United States). It was equipped with a 785 nm semiconductor laser, with a spectral measurement range of 3,300–50 cm^−1^, a spectral resolution of 2 cm^−1^, a laser power of 5.6 mW, and an integration time of 1.5 s (Ralbovsky and Lednev, 2020).

### 2.5 Elemental analysis of natural and artificially optimized realgar

#### 2.5.1 Inductively coupled plasma mass spectrometry (ICP-MS)

Accurately weigh approximately 0.2 g of each of realgar samples and place them into 50 mL Teflon digestion vessels. Then add 5 mL of aqua regia, 2 mL of hydrofluoric acid, and 1 mL of hydrogen peroxide to each vessel. Place the vessels on a graphite heating plate and digest at 120°C–200°C for 120–180 min, continuously adding the aforementioned acids until the samples are fully digested. After digestion, evaporate the acids, cool the samples, filter them, and dilute to a certain volume before testing for 65 trace elements. The instrument conditions are listed as follows: RF Power: 1,200 W, Carrier Gas Flow Rate (High Purity Argon): 0.7 L·min^−1^, Makeup Gas Flow Rate: 0.45 L·min^−1^, Pump Speed: 0.1 r·s^−1^, Sampling Depth: 8.0 mm (Aydemir et al., 2020).

#### 2.5.2 Measurement of total arsenic content

Using the titration method specified under “Content Determination” in the 2020 edition of the Chinese Pharmacopoeia for realgar, see Supplementary Material for detailed methodology. Orthogonal partial least squares discriminant analysis (OPLS-DA) was conducted with SIMCA 14.1 software.

### 2.6 Electron probe microanalyzer analysis (EMPA) of natural and artificially optimized realgar

A carbon film was coated on the surface of the thin section, and the defined locations were scanned to determine the distribution characteristics of 11 elements: As, S, Ca, Mg, Si, Na, Fe, Al, Pb, Zn, and Ti. Then, typical points in different mineral phases of the samples were selected for quantitative analysis using an electron probe (Electron Probe X-ray Microanalyzer, EMPA-1720, Shimadzu, Japan and Box Resistance Furnace, R-12, Shanghai Yutong Instruments) to determine their micro-area composition and the mass fractions of the elements. The instrument conditions are shown: accelerating voltage: 15 kV, beam current: 1 × 10^−^⁸ A, beam diameter: 5 μm (Yang et al., 2022).

### 2.7 Measurement of soluble As in the artificial environment

#### 2.7.1 Preparation of dissolution media and test solutions and standard solutions

The preparation of each dissolution medium was based on the 2020 edition of the Chinese Pharmacopoeia (National Pharmacopoeia Commission, 2020), see Supplementary Material for detailed methodology of dissolution media and test solutions.

#### 2.7.2 HPLC-ICP-MS

HPLC-ICP-MS was set as following: RF Power: 1,550 W; Carrier Gas Flow Rate (High Purity Argon): 0.65 L·min^−1^; Makeup Gas Flow Rate: 0.45 L·min^−1^; Pump Speed: 0.1 r·s^−1^; Sampling Depth: 8.0 mm (Favilli et al., 2022).

#### 2.7.3 Chromatographic conditions

The chromatographic column used was a Shimadzu Shim-pack VP-ODS anion exchange column (250 mm × 4.6 mm, 5 µm). The injection volume was 10 μL, and the mobile phase consisted of 10 mmol·L^−1^ sodium butanesulfonate, 4 mmol·L^−1^ malonic acid, 4 mmol·L^−1^ tetramethylammonium hydroxide, and 5 mmol·L^−1^ ammonium dihydrogen phosphate (0.5% methanol) with isocratic elution at a flow rate of 1.0 mL·min^−1^.

#### 2.7.4 Samples preparation

Fifteen batches of realgar samples were used for the determination of soluble arsenic content: six batches of natural realgar (N1, N2, N4, N8, N9, N10), six batches of artificially optimized realgar (S1, S4, S7, S8, S9, S10), and three batches of realgar slices (Y2, Y3, Y4).

### 2.8 Animal study

#### 2.8.1 Samples

Natural realgar (N1) and artificially optimized realgar (S1) were selected, both purchased from Bozhou Medicinal Herb Market. They were made into suspensions of different concentrations using 0.5% CMC-Na.

#### 2.8.2 Animals

Ninety SPF-grade Kunming mice, equally divided by sex, weighing 18–22 g, were purchased from Beijing Vital River Laboratory Animal Technology Co., Ltd. (Production License No. SCXK (Beijing) 2021-0011), see Supplementary Material for detailed animal housing conditions. SPF-grade Kunming mice were randomly divided into eight groups based on body weight: a control group (0.5% CMC-Na), low (NR-L), medium (NR-M), and high (NR-H) dosage groups of natural realgar (30, 270, 810 mg·kg^−1^, equivalent to 2, 18, and 54 times the maximum daily human dosage according to the Pharmacopoeia, respectively), and low (SR-L), medium (SR-M), and high (SR-H) dosage groups of artificially optimized realgar (30, 270, 810 mg·kg^−1^). Each group consisted of 10 mice, with equal numbers of males and females. After a 3-day acclimatization period, the mice were administered their respective treatments via gavage for 28 days, with a gavage volume of 0.2 mL·10 g^−1^.

#### 2.8.3 Urine sampling and blood preparation and systemic examination

Starting on day 25 of administration, one mouse from each group was placed in a metabolic cage to collect 12-h urine samples. This continued for three consecutive days until all urine samples were collected. Samples were stored at −80°C. Blood was collected from the orbital sinus of the mice into 2 mL centrifuge tubes, left at room temperature for 30 min, and then centrifuged at 3,000 rpm for 10 min. The upper serum layer was separated to measure ALT, AST, and BUN levels, with the remaining serum stored at −80°C. After blood collection from the orbital sinus, the mice were euthanized, and their bodies and liver and kidney organs were examined for obvious enlargement.

#### 2.8.4 Organ index and histopathological examination of organs

The liver and kidneys were harvested and weighed. The organ index was calculated using the formula: Organ Index (%) = (Organ Weight/Body Weight) × 100%. A liver lobe and a horizontally cut half kidney were fixed in 4% formaldehyde, dehydrated stepwise in alcohol, embedded in paraffin, sectioned into 3 μm thick slices, stained with HE, and examined under an optical microscope.

#### 2.8.5 Determination of total arsenic in blood samples

Serum (0.2 mL) was mixed with 0.7 mL nitric acid, heated in an 80°C water bath for 2 h until the solution was clear, diluted with 2.1 mL ultrapure water, mixed thoroughly, and measured using ICP-MS. The conditions are shown as follows, with a blank experiment conducted simultaneously: RF Power: 1,550 W; Carrier Gas Flow Rate: 0.65 L·min^−1^; Makeup Gas Flow Rate: 0.45 L·min^−1^; Pump Speed: 0.1 r·s^−1^; Sampling Depth: 8.0 mm.

#### 2.8.6 Determination of total arsenic in liver and kidney tissue

Liver tissue (0.3 g) was precisely weighed, mixed with 6 mL nitric acid and 1 mL hydrogen peroxide, and digested using microwave digestion. The digestion program is shown as follows: 100°C for 3 min; 130°C for 5 min; 150°C for 5 min; 170°C for 3 min; 190°C for 20 min. After digestion, the solution was diluted to 25 g with ultrapure water, mixed thoroughly, and measured using ICP-MS. Conditions are as shown in Tables 5–12, with a blank experiment conducted simultaneously. Approximately 0.2 g of kidney tissue was precisely weighed, mixed with 6 mL nitric acid and 1 mL hydrogen peroxide, and digested using microwave digestion. After digestion, the solution was diluted to 25 g with ultrapure water, mixed thoroughly, and measured using ICP-MS.

#### 2.8.7 Determination of arsenic species in urine

A certain amount of urine was diluted threefold with ultrapure water, mixed thoroughly, and filtered through a 0.45 μm filter membrane before measurement using ICP-MS, and the chromatographic column used was a Shimadzu Shim-pack VP-ODS anion exchange column (250 mm × 4.6 mm, 5 µm). The injection volume was 10 μL, and the mobile phase consisted of 10 mmol·L^−1^ sodium butanesulfonate, 4 mmol·L^−1^ malonic acid, 4 mmol·L^−1^ tetramethylammonium hydroxide, and 5 mmol·L^−1^ ammonium dihydrogen phosphate (0.5% methanol) with isocratic elution at a flow rate of 1.0 mL·min^−1^. A blank experiment was conducted simultaneously. This method references the preliminary work of the research group and the national standard GB 5009.11-2014 “National Food Safety Standard - Determination of Total Arsenic and Inorganic Arsenic in Food.”

### 2.9 Data analysis

Data analysis was performed using SPSS 26.0 statistical software. Measurement data were expressed as mean ± standard error (Mean ± SEM). For multiple group comparisons, if the data conformed to a normal distribution and met the homogeneity of variance test, one-way ANOVA was used; otherwise, Tamhane’s T2 (M) was applied. A *P*-value < 0.05 was considered statistically significant. GraphPad Prism 9 was used for graphing.

## 3 Result

### 3.1 Characteristics of realgar sample

**FIGURE 1:**
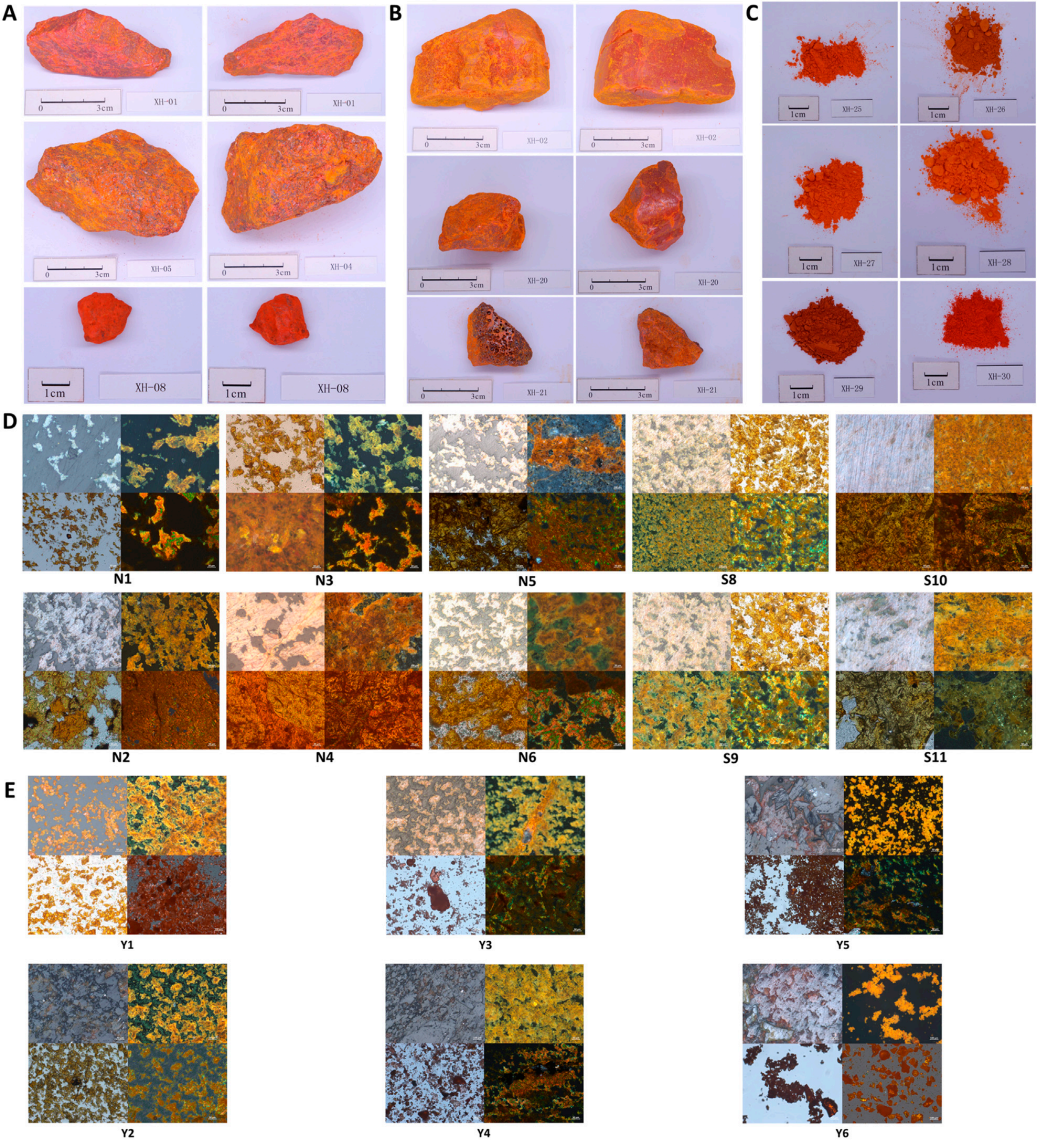
Characteristics of realgar samples. **(A)** The characteristic feature of natural realgar (1 cm), **(B)** The characteristic feature of artificially optimized realgar (1 cm), **(C)** The identification of characteristic features in realgar processed products (1 cm), **(D)** The polarized optical microscopic characteristics of natural realgar and artificially optimized realgar, **(E)** The microscopic characteristics of realgar processed products.

Following the requirements of 2020 edition of Pharmacopoeia of The People’s Republic of China, 30 samples collected from different resources were tested for characteristics including form, fragrance, and texture. Natural realgar typically appears in irregular blocks, deep red or orange in color, with diamond-like luster on the crystal surface. It is brittle and easily broken, with a resin-like luster on the fracture surface, as shown in Figure 1A. The remaining samples are shown in Supplementary Figure S1. Among them, XH-01, XH-05, XH-08, XH-10, XH-11, XH-13, XH-14, XH-15, XH-16, XH-19, XH-22, XH-23, and XH-24 conform to the above characteristics and have been renumbered as N1–N13. Artificially optimized realgar also appears in irregular blocks, deep red or orange-red in color. However, it is with a mostly smooth surface or honeycomb-like pores on the surface and is brittle, easily broken, with a slight distinctive odor and a bland taste, as shown in Figure 1B. The remaining samples are shown in Supplementary Figure S2. Samples XH-02, XH-03, XH-04, XH-06, XH-07, XH-09, XH-12, XH-17, XH-18, XH-20, and XH-21 display the characteristics of artificially optimized realgar more prominently and are labeled as S1–S11. Realgar processed products such as commercially realgar drinks are mostly in powdered form or as powder aggregates, ranging in color from orange-yellow to dark red, and lack luster. It is impossible to distinguish whether the raw material used is natural realgar or artificially optimized realgar with the naked eye. Samples XH-25 to XH-30 have been renumbered as Y1–Y6. The characteristic features are shown in Figure 1C.

As a result, our research above suggests that there are significant differences in the appearance and characteristics between natural realgar and artificially optimized realgar. However, realgar used in medicine is often in the form of orange-yellow powder, which makes identification much more challenging. Therefore, polarized light microscopy was used to observe the microscopic characteristics of realgar samples, providing a reference for the identification and analysis of artificially optimized and natural realgar. Under polarized light microscopy, both natural and artificially optimized realgar exhibit an irregular block structure with clear boundaries. Under reflected single polarized light, natural realgar samples N1 to N13 and artificially optimized realgar samples S1–S10 mostly appear gray-white, with indistinct microscopic features (see Figures 1A–D). Under reflected cross-polarized light, natural realgar predominantly shows orange-yellow crystals with numerous white crystals, presumed to be impurities like quartz and calcite. In contrast, artificially optimized realgar mainly displays orange-yellow realgar crystals with a significantly reduced number of white crystals (see Figures 1B–D). Under transmitted single polarized light, both natural and artificially optimized realgar appear orange-yellow and exhibit a mesh-like distribution, characteristic of heterogeneous bodies (see Figures 1C, D). Under transmitted cross-polarized light, both types of realgar show pleochroism, displaying green, yellow, and orange colors. This feature enables the rapid identification of realgar in compound preparations (see Figures 1D, E). The microscopic features of the remaining samples are shown in Supplementary Figure S3.

Under reflected single polarized light, realgar processed products Y1–Y6 mostly appear pale yellow, with indistinct microscopic features (see Figures 1A–E). Under reflected cross-polarized light, realgar processed products predominantly display orange-yellow crystals, with a significant variation in impurity content among different pieces, indicating inconsistent quality in commercially available products (see Figures 1B–E). Under transmitted single polarized light, realgar processed products appear dark red (see Figures 1C–E). Under transmitted cross-polarized light, realgar processed products mostly show an orange-red granular distribution with more distinct microscopic features (see Figures 1D, E).

### 3.2 Spectral characteristics of natural and artificially optimized realgar

Currently, four crystal structures of realgar have been identified: α-As₄S₄, β-As₄S₄, χ-AsS, and pararealgar (Bonazzi et al., 1996; Douglass et al., 1992; Ballirano and Maras, 2002). Among these, α-As₄S₄ exists as a low-temperature phase, while β-As₄S₄ is typically obtained by heating above 252°C. χ-AsS is an intermediate phase in the transformation of realgar to pararealgar, which is a photoinduced polymorph. This study performed X-ray diffraction analysis on 30 samples collected from different sources to observe the phase structure differences between natural and artificially optimized realgar.

**FIGURE 2:**
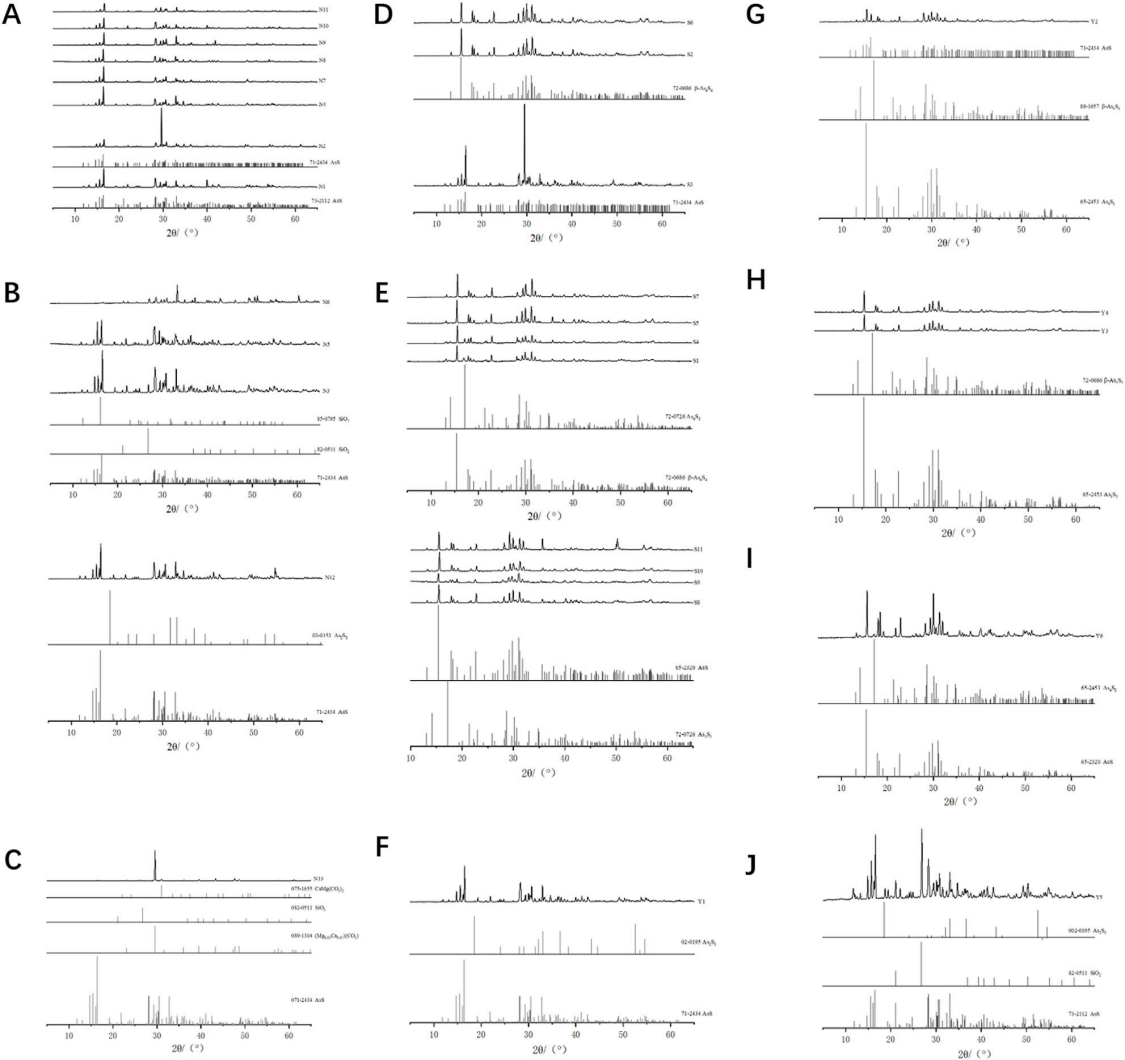
Spectral characteristics of natural and artificially optimized realgar. **(A)** XRD profiles of natural realgar one phases and standard PDF card, **(B)** XRD profiles of natural realgar two phases and standard PDF card, **(C)** XRD profiles of natural realgar four phases and standard PDF card, **(D)** XRD profiles of artificially optimized realgar one phases and standard PDF card, **(E)** XRD profiles of artificially optimized realgar two phases and standard PDF card, **(F)** XRD profiles of realgar processed product Y1 and standard PDF card, **(G)** XRD profiles of realgar processed product Y2 and standard PDF card, **(H)** XRD profiles of realgar processed product Y3, Y4 and standard PDF card, **(I)** XRD profiles of realgar processed product Y6 and standard PDF card, **(J)** XRD profiles of realgar processed product Y5 and standard PDF card.

For natural realgar, the XRD spectra of natural realgar samples N1, N2, N4, and N7–N11 each correspond to a single phase. The characteristic peak data were matched to PDF card numbers 73-2112 and 71-2434, identifying the substance as Realgar with the chemical formula AsS (see Figure 2A). The XRD spectra of natural realgar samples N3, N5, and N6 each contain two phases. The characteristic peak data correspond to PDF card numbers 82-0511 (85-0795) and 71-2434, identifying the substances as Quartz and Realgar, with chemical formulas SiO₂ and AsS, respectively. The XRD spectrum of natural realgar sample N12 also consists of two phases. The characteristic peaks correspond to PDF card numbers 03-0153 and 71-2434, identifying the substances as Orpiment and Realgar, with chemical formulas As₂S₃ and AsS (see Figure 2B). The XRD spectrum of natural realgar sample N13 contains four phases. The characteristic peak data correspond to PDF card numbers 89-1304, 82-0511, 75-1655, and 71-2434, identifying the substances as Calcite, Magnesite, Quartz, Realgar, and Dolomite, with chemical formulas (Mg₀.₀₃Ca₀.₉₇) (CO₃), SiO₂, CaMg (CO₃)₂, and AsS (see Figure 2C).

The XRD spectra of artificially optimized realgar samples S2, S3, and S6 each correspond to a single phase. The characteristic peak data were matched to PDF card numbers 72-0686 and 71-2434, identifying the substances as Pararealgar (syn) and Realgar, with chemical formulas β-As₄S₄ and AsS (see Figure 2D). The XRD spectra of artificially optimized realgar samples S1, S4, S5, and S7 each contain two phases. The characteristic peak data correspond to PDF card numbers 72-0686 and 65-2453, identifying the substances as Pararealgar (syn) and Arsenic Sulfide, with chemical formulas β-As₄S₄ and As₄S₃. The XRD spectra of artificially optimized realgar samples S8–S11 also consist of two phases. The characteristic peak data correspond to PDF card numbers 65-2320 and 72-0726, identifying the substances as Arsenic Sulfide and Dimorphite I (syn), with chemical formulas AsS and As₄S₃ (see Figure 2E).

The XRD spectrum of realgar processed products Y1 consists of two phases. The characteristic peak data were matched to PDF card numbers 71-2434 and 02-0195, identifying the substances as Realgar and Orpiment, with chemical formulas AsS and As₂S₃ (see Figure 2F). The XRD spectrum of realgar processed products Y2 corresponds to PDF card numbers 71-2434 (88-1657) and 65-2453, identifying the substances as Realgar (Alacranite, syn) and Arsenic Sulfide, with chemical formulas AsS (β-As₄S₄) and As₄S₃ (see Figure 2G). The XRD spectra of realgar processed products Y3 and Y4 correspond to PDF card numbers 72-0686 and 65-2453, identifying the substances as Pararealgar (syn) and Arsenic Sulfide, with chemical formulas β-As₄S₄ and As₄S₃ (see Figure 2H). The XRD spectrum of realgar processed products Y6 corresponds to PDF card numbers 65-2320 and 65-2453, identifying both substances as Arsenic Sulfide, with chemical formulas β-As₄S₄ and As₄S₃ (see Figure 2I). The XRD spectrum of realgar processed products Y5 contains three phases. The characteristic peak data were matched to PDF card numbers 02-0195, 82-0511, and 73-2112, identifying the substances as Orpiment, Quartz, and Realgar, with chemical formulas As₂S₃, SiO₂, and AsS (see Figure 2J).

### 3.3 Raman spectroscopy of natural and artificially optimized realgar

Current research indicates that within the infrared spectrum range of 400–4,000 cm^−1^, realgar exhibits no distinctive absorption peaks other than those caused by moisture interference (Wu et al., 2022). Therefore, infrared spectroscopy is not suitable for characterizing realgar. In contrast, studies show that Raman spectroscopy effectively highlights the characteristic absorption peaks of realgar and offers advantages such as speed, simplicity, and accuracy.

**FIGURE 3:**
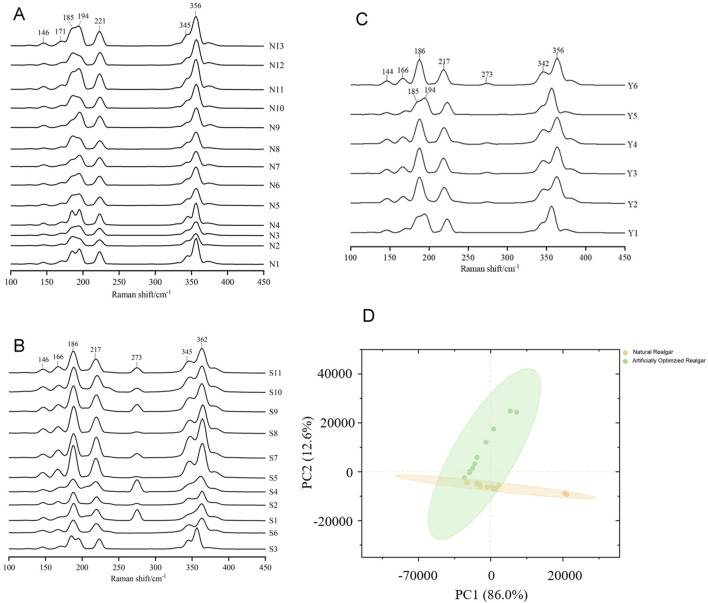
Raman spectrum of natural and artificially optimized realgar. **(A)** Comparison among Raman spectrum from samples of natural realgar, **(B)** Comparison among Raman spectrum from samples of artificially optimized realgar, **(C)** Comparison among Raman spectrum from samples of realgar processed products, **(D)** Spatial scatter diagram of principal component analysis of natural realgar and artificially optimized realgar.

The Raman spectrum of natural realgar shows strong absorption peaks at wavelengths 356, 345, 221, 194, 185, 171, and 146 cm^−1^, with good spectral consistency and similar peak positions across samples. Peaks at 185 and 194 cm^−1^ appear as double peaks, and there is a shoulder peak at 345 cm^−1^ to the left of the 356 cm^−1^ peak, which is weaker (see Figure 1C). These results align with literature reports where characteristic peaks for realgar are noted at 183, 194, 221, and 354 cm^−1^ (Scheuermann and Ritter, 1969; Luo, 2018). The peaks at 185 and 194 cm^−1^ correspond to As-As stretching vibrations, the strong signal at 221 cm^−1^ results from the combined bending vibrations of S-As-S and stretching vibrations of As-S, and the peak at 354 cm^−1^ is attributed to S-As-S stretching vibrations. The Raman spectrum of artificially optimized realgar shows strong absorption peaks at wavelengths 362, 345, 273, 217, 186, 166, and 146 cm^−1^. Except for a shoulder peak at 345 cm^−1^ to the left of 362 cm^−1^, all other peaks are single peaks (see Figure 2C). Unlike natural realgar, the artificially optimized realgar exhibits an absorption peak at 273 cm^−1^ (Figures 3A, B). Literature indicates that the differences in peak positions between natural and artificially optimized realgar are due to differences in their phase compositions, with the absorption peaks of artificially optimized realgar matching the Raman characteristic spectrum of Pararealgar (Kadıoğlu et al., 2009; Figures 3A, B). The Raman spectra of processed realgar products Y2, Y3, Y4, and Y6 align with the strong absorption peaks of artificially optimized realgar, while Y1 and Y5 match the peak positions of natural realgar (see Figure 3C). This suggests that Raman spectroscopy could serve as a rapid and accurate method for distinguishing between natural and artificially optimized realgar decoctions.

Principal Component Analysis (PCA) of the Raman spectrum data for 24 samples in the wavenumber range of 500–100 cm^−1^ (see Figure 3D) shows that PC1 accounts for 86.0% of the total variance, and PC2 accounts for 12.6%, with a cumulative contribution of 98.6%. The first two principal components capture most of the variance. Natural realgar samples show a clustering tendency, while artificially optimized realgar samples are more dispersed, with no overlap between the two categories. This indicates that combining Raman spectroscopy with PCA analysis can effectively classify natural and artificially optimized realgar.

### 3.4 Elemental analysis of natural and artificially optimized realgar

Elements in realgar is closely associated with its therapeutic effects. According to the 2020 edition of the Chinese Pharmacopoeia, the arsenic content in realgar, calculated as diarsenic trisulfide (As₂S_3_), should not be less than 90%. The titration results of 13 batches of natural realgar samples showed that five batches (N2, N3, N5, N6, and N13) did not meet the specified value. Among the 11 batches of artificially optimized realgar samples, only one batch (S3) did not meet the specified value. Additionally, in six batches of realgar slices, Y1 and Y5 did not meet the specified content standards. Although the determination of total arsenic content provides some regulation on the quality of realgar, it is not effective in distinguishing between natural and artificially optimized realgar. Moreover, the total arsenic content in artificially optimized realgar is generally higher than that in natural realgar. The content results are shown in Table 1.

**TABLE 1:** Total arsenic content in natural realgar, artificially optimized realgar and its processed products.

Sample number	Total arsenic content/%	Sample number	Total arsenic content/%
N1	92.4	S3	84.5
N2	78.2	S4	91.7
N3	87.6	S5	99.7
N4	96.5	S6	97.1
N5	35.0	S7	100.3
N6	44.5	S8	99.8
N7	94.8	S9	99.4
N8	95.1	S10	97.9
N9	98.3	S11	90.5
N10	97.8	Y1	76.2
N11	98.0	Y2	91.8
N12	90.3	Y3	98.5
N13	6.5	Y4	100.2
S1	97.0	Y5	42.7
S2	96.7	Y6	98.3

**FIGURE 4:**
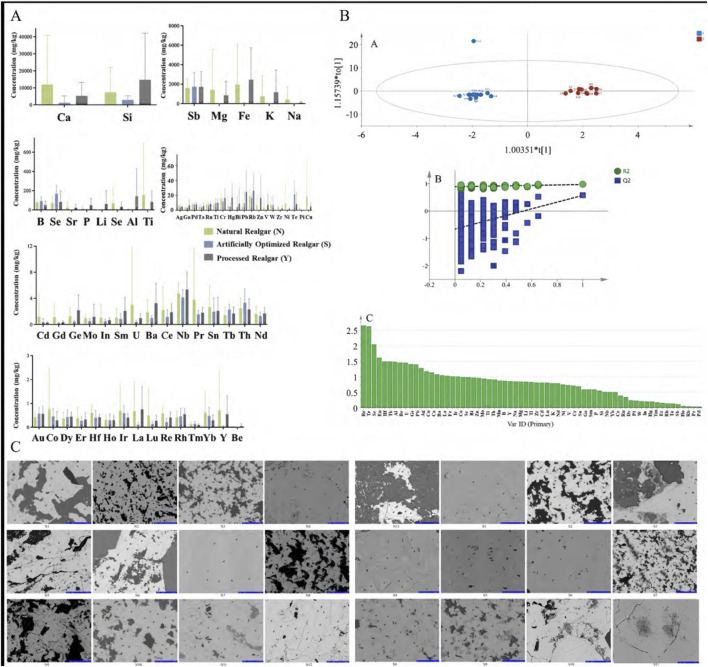
Elemental analysis of natural and artificially optimized realgar. **(A)** Content difference of natural realgar, artificial optimized realgar and its processed products; **(B)** OPLS-DA **(A)**, permutation test of permutation test of OPLS- DA model **(B)**, VIP value plot **(C)** of natural and artificially optimized realgar; **(C)** Electron microprobe scans of natural and artificially optimized realgar (40 μm).

In total, 65 inorganic elements were detected in natural realgar, artificially optimized realgar, and realgar slices. According to the data in Tables 2–4, the major elements in natural realgar, ranked from highest to lowest average content, are Ca (11946.3 mg·kg^−1^), Mg (1417.6 mg·kg^−1^), K (736.6 mg·kg^−1^), Na (404.5 mg·kg^−1^), and P (12.8 mg·kg^−1^). The trace elements with higher contents are Si (7352.0 mg·kg^−1^), Fe (1964.1 mg·kg^−1^), Sb (1610.5 mg·kg^−1^), Ti (154.1 mg·kg^−1^), and B (79.9 mg·kg^−1^). Among heavy metals and harmful elements, Cu has the highest content with an average of 18.4 mg·kg^−1^, followed by Hg (5.7 mg·kg^−1^) and Pb (4.9 mg·kg^−1^), with Cd having the lowest content at an average of 1.2 mg·kg^−1^. For artificially optimized realgar, the major element with the highest content is Ca (1273.0 mg·kg^−1^), followed by Na (19.8 mg·kg^−1^), K (9.2 mg·kg^−1^), Mg (6.2 mg·kg^−1^), and P with the lowest content (2.6 mg·kg^−1^). The trace elements, ranked from highest to lowest average content, are Si (2896.1 mg·kg^−1^), Sb (1743.2 mg·kg^−1^), Se (168.6 mg·kg^−1^), B (92.1 mg·kg^−1^), and Fe (48.0 mg·kg^−1^). Among heavy metals and harmful elements, Pb has the highest content with an average of 9.7 mg·kg^−1^, and Cd has the lowest content at an average of 0.3 mg·kg^−1^. These results indicate significant differences in the inorganic element content between natural realgar and artificially optimized realgar, with natural realgar generally having higher overall inorganic element content compared to artificially optimized realgar, as shown in Figure 4A.

**TABLE 2:** Content of trace elements in natural realgar (mg·kg^−1^).

Element	N1	N2	N3	N4	N5	N6	N7	N8	N9	N10	N11	N12	N13
Ag	1.77	14.60	4.76	7.07	1.56	9.00	3.80	6.10	9.56	10.09	8.21	4.01	1.36
Al	0.43	12.37	13.37	1.59	13.56	2.53	9.01	0.48	0.34	2.67	25.55	9.37	6.06
Au	0.56	0.28	0.72	0.01	0.27	0.70	0.59	0.48	0.75	0.29	0.30	0.42	0.24
B	61.68	35.06	134.56	92.50	128.10	82.49	92.26	49.32	107.12	72.19	73.90	47.99	61.32
Ba	0.52	5.42	1.83	0.67	3.29	0.91	0.90	0.46	1.07	1.00	1.03	0.66	6.43
Be	0.00	0.01	0.01	0.01	0.20	0.01	0.02	0.01	0.02	0.01	0.01	0.03	0.01
Bi	0.77	0.48	0.62	0.77	0.79	0.02	0.38	1.27	0.24	1.74	0.32	0.35	0.55
Ca	27.93	35130.52	47.66	30.72	7686.14	102.45	1113.49	22.19	29.73	33.84	158.94	8429.96	102487.97
Cd	0.19	0.01	0.13	0.17	12.82	0.03	0.20	0.06	0.17	0.14	0.25	0.01	0.80
Ce	0.32	0.13	0.31	0.89	12.10	0.08	1.67	1.09	2.42	1.95	0.35	0.15	7.34
Co	0.22	0.01	0.44	0.08	6.42	0.37	0.47	0.22	0.71	0.13	0.75	0.03	0.04
Cr	7.02	10.62	15.23	11.09	31.31	13.79	17.60	5.94	19.91	12.99	10.94	8.39	32.12
Cu	0.21	0.96	0.86	0.45	187.35	0.56	1.40	0.06	32.72	0.27	0.39	2.76	11.64
Dy	0.08	0.51	0.95	0.40	0.03	0.52	0.62	0.04	0.31	0.39	0.53	0.38	0.16
Er	0.06	0.08	0.20	0.15	1.38	0.01	0.09	0.12	0.33	0.02	0.70	0.50	1.32
Eu	0.02	0.11	0.05	0.02	0.37	0.03	0.05	0.01	0.06	0.09	0.04	0.06	0.26
Fe	6.65	438.36	171.11	1.11	11509.89	519.33	283.30	15.63	0.22	154.02	329.82	1046.78	11057.28
Ga	1.37	4.18	0.89	4.25	13.48	2.49	1.91	1.22	3.32	3.66	5.07	3.46	3.33
Gd	0.51	0.87	0.10	0.71	5.41	0.11	0.25	0.04	0.22	0.04	0.48	0.81	4.96
Ge	0.75	0.09	0.37	0.86	2.14	1.98	0.78	0.78	0.77	0.61	4.50	0.25	2.61
Hf	0.62	0.44	0.49	0.12	0.25	0.98	0.40	0.16	0.84	0.81	0.60	0.89	1.22
Hg	5.77	3.18	6.60	0.77	4.04	0.55	1.88	0.20	44.59	0.97	2.37	3.16	0.55
Ho	0.50	0.15	0.15	0.13	0.45	0.13	0.39	0.22	0.47	0.65	0.73	0.07	0.01
In	0.33	0.81	0.24	0.94	77.18	0.14	0.64	0.00	0.69	0.64	0.14	0.65	0.46
Ir	0.82	0.14	0.46	0.32	3.96	0.03	0.75	0.56	0.10	0.51	0.30	0.46	0.65
K	0.64	5.24	1.23	0.60	7689.83	0.99	0.15	2.15	0.04	0.77	2.12	0.20	1872.26
La	0.14	0.31	0.28	0.14	3.32	0.06	0.31	0.20	0.11	0.17	0.02	0.18	3.58
Li	0.06	0.08	0.03	0.09	0.77	0.08	0.04	0.09	0.12	0.06	0.21	0.17	2.43
Lu	0.06	0.12	0.04	0.18	4.17	0.03	0.31	0.15	0.33	0.08	0.61	0.07	0.36
Mg	2.49	120.11	6.88	5.08	3337.11	4.26	11.76	1.70	1.36	4.00	41.94	33.33	14858.22
Mn	0.04	30.80	0.01	0.12	247.54	0.03	0.37	0.02	0.16	0.24	2.84	7.60	577.06
Mo	0.56	2.43	0.57	0.83	0.19	0.82	1.93	0.81	0.58	0.55	0.88	17.70	0.30
Na	14.11	68.04	24.36	24.23	151.13	17.74	27.02	15.86	27.30	26.49	18.43	14.93	4828.64
Nb	2.89	2.91	5.33	3.62	7.96	4.12	3.44	2.88	5.62	5.21	6.99	4.48	6.61
Nd	0.39	1.07	1.59	1.31	0.46	0.79	0.50	1.05	1.59	2.92	2.24	2.49	4.48
Ni	0.33	0.86	0.10	0.29	101.70	0.35	0.31	0.46	0.24	0.64	0.25	0.15	12.20
P	0.98	0.16	0.74	0.58	102.46	0.71	0.99	0.16	0.26	0.63	0.54	0.16	58.67
Pb	0.87	0.69	5.05	2.52	33.04	0.81	0.49	0.05	0.43	0.75	0.25	3.45	14.85
Pd	5.49	5.60	8.84	7.15	1.26	6.36	7.09	4.32	10.70	10.73	12.52	9.83	7.64
Pr	0.98	1.20	1.84	1.12	29.77	1.74	1.60	0.96	2.95	1.23	2.91	1.62	1.15
Pt	0.24	0.10	0.37	0.06	259.79	0.36	0.94	0.69	0.98	0.53	0.64	0.98	0.72
Rb	11.77	7.99	18.84	13.21	40.72	13.46	14.38	7.56	21.69	21.71	29.65	22.16	21.23
Re	0.67	0.64	0.79	0.25	0.20	0.93	0.71	0.37	0.01	0.38	0.81	0.90	0.87
Rh	0.14	0.66	0.97	0.46	0.07	0.35	0.27	0.25	0.77	0.23	0.14	0.31	0.72
Ru	1.83	3.62	6.56	2.87	13.73	3.66	6.54	1.56	9.40	5.00	1.42	2.24	7.15
Sb	935.75	638.98	2991.18	1411.79	531.91	3074.87	1740.49	3096.82	1644.09	2130.97	953.62	1117.63	668.89
Se	45.57	136.54	27.58	33.79	16.27	24.32	220.02	49.80	57.40	45.21	52.99	202.97	9.49
Si	2230.19	179.09	4503.09	2135.48	35435.16	1404.40	1453.39	684.63	1307.28	631.84	563.59	317.26	44730.89
Sm	0.67	0.37	0.89	0.54	5.40	0.32	0.86	0.48	0.68	0.17	1.78	0.37	0.75
Sn	0.08	4.85	4.83	2.29	0.46	0.16	1.96	0.16	2.46	4.82	0.92	0.35	11.39
Sr	0.24	32.47	0.63	0.36	59.67	0.40	6.36	0.19	0.44	0.41	1.24	3.43	211.21
Ta	4.47	3.59	6.71	4.89	6.91	6.75	6.91	5.13	9.57	8.01	15.53	10.35	11.28
Tb	1.92	0.29	1.94	0.44	0.91	0.80	0.44	0.15	0.61	1.03	4.29	2.85	2.75
Te	4.87	0.81	0.42	0.02	12.78	1.00	0.08	0.66	0.22	0.94	0.56	0.34	0.54
Th	0.32	2.79	2.19	1.48	1.58	2.81	1.13	0.44	3.16	4.46	1.17	5.11	5.28
Ti	0.07	1.28	0.67	0.12	1939.39	0.12	1.07	0.04	0.06	0.00	2.18	1.44	57.02
Tl	2.39	5.62	7.58	6.59	10.87	8.15	1.20	6.47	0.41	8.93	15.90	6.84	20.04
Tm	0.08	0.14	0.03	0.11	0.12	0.19	0.08	0.04	0.03	0.34	0.12	0.17	0.28
U	0.49	0.10	0.99	0.48	32.12	0.64	0.40	0.63	0.49	1.93	0.54	0.24	0.61
V	0.01	0.37	0.11	0.13	46.35	0.17	0.18	0.14	0.56	0.05	0.13	0.33	7.75
W	0.26	10.49	14.33	0.84	0.41	20.31	0.42	3.76	0.93	0.77	0.11	0.26	41.99
Y	0.01	0.38	0.05	0.02	3.74	0.01	0.02	0.02	0.01	0.02	0.04	0.05	4.81
Yb	2.56	0.08	0.39	0.02	0.67	0.03	0.67	0.40	0.44	0.16	0.15	0.00	2.49
Zn	0.72	0.95	0.53	0.52	47.48	0.71	0.50	0.60	0.83	0.97	0.73	0.60	26.23
Zr	0.06	0.85	0.01	0.04	42.61	0.10	0.16	0.09	0.51	0.03	0.80	0.71	9.57

**TABLE 3:** Content of trace elements in artificial optimized realgar (mg·kg^−1^).

Element	S1	S2	S3	S4	S5	S6	S7	S8	S9	S10	S11
Ag	3.57	2.05	2.75	1.64	9.48	1.65	4.70	5.28	2.50	8.42	5.92
Al	0.20	4.60	3.89	3.05	4.66	2.01	2.36	0.98	0.30	0.75	4.00
Au	0.70	0.17	0.46	0.79	0.48	0.56	0.90	0.35	0.38	0.52	0.99
B	109.10	108.69	65.84	155.87	164.19	140.89	70.01	36.99	35.51	68.29	58.07
Ba	0.74	1.41	0.55	1.16	1.54	1.09	1.35	0.40	0.75	0.97	0.88
Be	0.02	0.01	0.00	0.00	0.01	0.01	0.01	0.00	0.00	0.01	0.01
Bi	0.81	0.19	0.26	0.65	0.61	0.54	0.59	0.51	0.80	0.22	47.92
Ca	62.91	138.40	13391.69	59.64	119.24	90.63	23.49	12.60	33.53	35.35	35.86
Cd	0.48	0.04	0.05	0.19	0.02	0.46	0.15	0.02	0.10	0.07	1.95
Ce	2.38	1.28	0.06	1.63	3.51	0.98	0.08	0.32	0.07	0.77	1.52
Co	0.02	0.60	0.87	0.99	0.45	0.38	0.27	0.11	0.52	0.74	0.08
Cr	16.23	10.13	5.53	14.13	15.39	17.64	7.68	12.19	7.01	11.15	13.91
Cu	0.20	0.87	0.48	0.34	0.81	0.65	0.47	0.11	0.56	0.38	0.05
Dy	0.68	0.13	0.35	0.84	0.04	0.65	0.28	0.15	0.88	0.61	0.12
Er	0.51	0.19	0.33	0.26	0.38	0.59	0.19	0.00	0.08	0.35	0.10
Eu	0.05	0.01	0.06	0.03	0.03	0.01	0.01	0.03	0.02	0.02	0.02
Fe	20.78	75.53	38.76	0.67	0.17	0.46	35.34	21.69	14.36	192.04	128.64
Ga	2.51	3.26	4.28	1.85	2.74	6.10	4.86	2.62	1.69	2.50	2.54
Gd	0.14	0.30	0.09	0.23	0.06	0.55	0.17	0.09	0.03	0.11	0.08
Ge	0.43	0.39	0.65	0.28	0.00	0.46	0.57	0.78	0.27	0.51	0.52
Hf	0.11	0.76	0.45	0.00	0.01	0.04	0.50	0.50	0.66	0.75	0.63
Hg	0.28	15.91	0.33	9.03	0.98	2.79	9.14	2.95	5.28	5.04	4.76
Ho	0.26	0.60	0.03	0.49	0.01	0.91	0.25	0.08	0.17	0.23	0.22
In	0.87	0.73	0.02	0.32	0.39	0.52	0.39	0.63	0.27	0.30	0.91
Ir	0.20	0.87	0.60	0.85	0.98	0.38	0.27	0.94	0.87	0.17	0.35
K	11.61	14.99	0.66	2.51	1.32	0.06	10.99	11.20	12.12	15.27	20.96
La	0.07	0.25	0.09	0.27	0.14	0.05	0.04	0.02	0.16	0.07	0.07
Li	0.15	0.14	0.05	0.06	0.04	0.05	0.09	0.05	0.20	0.03	0.12
Lu	0.32	0.01	0.11	0.20	0.08	0.08	0.20	0.10	0.03	0.20	0.12
Mg	2.27	3.53	35.88	1.88	10.45	3.00	2.80	0.43	4.22	2.41	1.69
Mn	0.19	1.09	14.99	0.11	0.04	0.22	0.54	0.37	0.18	0.61	0.44
Mo	0.35	0.13	0.27	0.96	0.41	0.71	0.52	0.64	0.05	0.92	0.98
Na	16.97	24.19	17.39	25.67	30.91	23.07	15.20	8.79	13.91	24.46	17.41
Nb	5.04	4.74	2.77	4.85	5.71	4.47	2.99	2.75	2.29	4.93	5.01
Nd	1.08	2.14	0.41	1.89	1.10	2.03	0.80	0.63	0.89	1.86	1.46
Ni	1.57	0.20	0.51	0.38	0.39	0.72	0.15	0.43	0.40	0.69	0.06
P	0.99	0.26	16.42	0.98	4.46	0.63	0.83	2.99	0.93	0.18	0.40
Pb	5.02	9.13	1.85	5.82	4.61	0.30	6.02	7.46	5.68	7.25	53.84
Pd	9.06	7.12	4.72	10.05	10.14	10.37	5.89	5.68	4.73	8.32	9.35
Pr	2.05	1.82	0.46	2.53	1.65	1.03	1.21	1.13	1.72	1.87	1.69
Pt	0.34	0.14	0.66	0.66	0.68	0.78	0.56	0.06	0.94	0.14	0.75
Rb	19.65	18.56	8.81	21.75	18.99	20.44	11.27	10.83	10.40	21.82	21.12
Re	0.10	0.13	0.68	0.32	0.12	0.05	0.10	0.15	0.17	0.32	0.16
Rh	0.98	0.68	0.10	0.14	0.78	0.16	0.79	0.71	0.48	0.05	0.27
Ru	5.29	2.82	1.24	4.23	5.16	6.83	2.12	4.58	2.09	3.90	6.14
Sb	777.33	1522.02	729.66	1374.27	1456.00	1577.15	1111.91	3099.49	1027.98	905.68	5593.53
Se	179.50	228.83	12.91	171.70	147.22	168.12	201.91	332.54	183.59	201.02	27.33
Si	4254.61	4628.30	2810.41	6552.60	6652.74	3833.12	1375.77	390.84	340.72	566.36	451.33
Sm	0.41	0.37	0.44	0.12	2.63	2.06	0.94	0.86	0.03	1.28	0.06
Sn	0.36	1.43	0.36	3.23	0.86	7.52	1.58	2.07	2.82	0.98	0.37
Sr	0.30	0.36	6.72	0.43	0.79	0.46	0.22	0.13	0.22	0.34	0.29
Ta	6.59	8.70	3.80	8.47	10.90	10.18	4.84	5.11	4.06	8.90	9.71
Tb	3.20	1.83	2.25	2.67	2.53	1.68	1.29	1.18	1.81	2.30	4.07
Te	18.07	25.87	0.97	46.45	0.36	0.48	49.14	30.53	40.27	19.68	0.97
Th	7.52	3.33	1.66	6.03	2.46	2.51	1.42	3.97	0.63	1.74	5.15
Ti	0.03	0.19	0.28	0.13	0.28	0.01	0.08	0.08	0.01	0.09	0.19
Tl	7.45	8.40	5.46	0.93	12.91	9.64	7.56	7.62	8.05	9.10	28.55
Tm	0.33	0.22	0.03	0.15	0.08	0.21	0.02	0.06	0.16	0.24	0.07
U	0.22	0.98	0.42	0.28	0.63	0.18	0.15	0.05	0.37	0.64	0.23
V	0.35	0.01	1.90	0.12	0.12	0.44	0.08	0.15	0.13	0.01	0.26
W	0.42	5.99	15.56	0.02	0.62	38.37	0.30	0.23	0.14	0.36	13.48
Y	0.02	0.03	0.13	0.01	0.04	0.05	0.05	0.05	0.02	0.03	0.08
Yb	0.75	1.26	0.12	0.60	0.69	0.15	0.75	0.16	0.01	0.47	0.33
Zn	0.71	0.52	0.22	0.69	0.48	0.00	0.56	0.06	0.23	0.39	0.38
Zr	0.41	0.24	0.53	0.71	0.45	0.20	0.24	0.15	0.01	0.42	0.03

**TABLE 4:** Content of trace elements in realgar processed products (mg·kg^−1^).

Element	Y1	Y2	Y3	Y4	Y5	Y6
Ag	5.99	4.28	2.86	6.95	4.67	5.66
Al	7.92	719.11	20.15	80.62	19.09	4.72
Au	0.11	0.99	0.43	0.46	0.54	0.87
B	25.28	38.68	36.78	54.09	127.30	28.18
Ba	1.56	4.95	1.41	2.21	8.71	0.68
Be	0.02	0.02	0.00	0.01	0.25	0.01
Bi	0.59	0.00	52.06	0.40	0.45	1.45
Ca	11833.46	873.23	148.33	283.03	18490.26	321.68
Cd	0.30	0.09	0.34	0.19	0.53	0.17
Ce	1.67	2.12	1.12	2.32	3.95	0.22
Co	0.58	0.92	0.05	0.01	0.09	0.12
Cr	13.24	9.11	7.97	30.76	34.33	5.00
Cu	11.87	6.99	2.44	0.15	10.01	0.00
Dy	0.19	0.53	0.52	0.69	0.52	0.24
Er	0.49	0.01	0.34	0.31	0.75	0.09
Eu	0.29	0.01	0.03	0.02	0.41	0.02
Fe	3410.93	2250.44	82.42	396.63	8560.46	74.27
Ga	21.27	3.63	1.57	6.30	7.23	1.74
Gd	0.68	0.15	0.08	0.35	0.52	0.17
Ge	0.79	2.00	0.91	6.91	1.63	0.79
Hf	0.56	0.58	0.29	0.42	0.33	0.35
Hg	3.13	49.83	5.40	27.94	7.27	8.84
Ho	0.04	0.25	0.40	0.19	0.64	0.28
In	0.80	0.89	0.01	0.56	0.96	0.79
Ir	0.35	0.29	0.69	0.17	0.45	0.49
K	1197.80	97.05	4.21	12.50	5678.80	11.96
La	0.93	0.42	0.03	0.40	2.58	0.18
Li	0.55	23.42	0.19	1.20	344.93	0.04
Lu	0.07	0.22	0.34	0.19	0.71	0.13
Mg	3677.78	271.79	41.75	28.32	1094.17	10.55
Mn	63.69	14.55	1.78	2.12	113.80	0.56
Mo	5.06	0.14	0.24	0.99	0.29	0.31
Na	104.64	15.43	26.54	27.19	350.10	34.02
Nb	4.38	4.30	5.16	5.09	10.54	2.70
Nd	0.94	1.86	1.41	2.07	3.17	0.73
Ni	2.39	0.60	0.54	0.44	8.29	0.82
P	102.33	0.30	0.68	28.96	161.62	0.21
Pb	0.78	3.28	52.29	14.86	69.00	7.33
Pd	5.15	7.63	8.03	9.97	11.73	5.50
Pr	1.08	1.52	0.94	3.18	2.15	1.96
Pt	0.60	0.89	0.13	0.07	0.45	0.05
Rb	19.07	17.29	17.58	24.97	70.81	10.08
Re	0.18	0.17	0.40	0.55	0.40	0.77
Rh	0.44	0.36	0.80	0.89	0.46	0.36
Ru	3.15	3.32	4.55	14.09	7.58	1.60
Sb	1762.79	1080.15	4753.34	1567.24	389.33	824.62
Se	74.21	1.73	130.53	0.68	1.73	290.53
Si	17119.38	1468.50	255.46	407.99	69002.51	124.54
Sm	0.14	0.13	0.50	2.42	0.27	0.61
Sn	5.32	0.23	2.09	0.01	3.70	1.18
Sr	39.66	5.96	0.88	5.74	79.71	0.54
Ta	6.63	8.61	6.26	11.12	14.22	4.45
Tb	1.81	3.55	1.43	1.53	0.95	0.84
Te	0.87	0.47	9.09	0.37	0.95	38.72
Th	4.66	3.19	0.50	1.85	0.64	2.91
Ti	239.55	44.35	0.81	3.99	213.23	0.21
Tl	5.97	10.05	18.41	17.07	3.63	6.10
Tm	0.08	0.07	0.10	0.07	0.18	0.09
U	0.76	0.99	0.47	0.71	2.19	0.75
V	7.06	1.21	0.27	0.41	37.02	0.14
W	0.80	0.29	0.49	0.07	0.22	0.46
Y	1.08	0.18	0.09	0.02	1.92	0.02
Yb	0.22	0.71	0.07	0.25	0.34	0.19
Zn	20.21	1.96	1.06	0.23	77.33	0.61
Zr	6.56	0.59	0.39	0.29	6.95	0.06

Using 65 common inorganic elements as dependent variables and 24 batches of different types of realgar medicinal materials as independent variables, orthogonal partial least squares discriminant analysis (OPLS-DA) was conducted with SIMCA 14.1 software (Figures 4A, B). This analysis effectively distinguishes between natural and artificially optimized realgar. In this analysis, the fitting index for the independent variables (Rx^2^) is 0.537, the fitting index for the dependent variables (Ry^2^) is 0.974, and the model prediction index (Q^2^) is 0.575. According to relevant literature, R^2^ and Q^2^ values above 0.5 indicate that the model fitting results are acceptable. After 200 model permutation tests, as shown in Figure 4B, the intersection point of the Q^2^ regression line with the vertical axis is less than 0, indicating that the model is not overfitted and that the model validation is effective. Therefore, the results can be used for distinguishing between natural and artificially optimized realgar. Figures 4B, C shows the variable importance in the projection (VIP) for different elements, where elements with VIP > 1 are Re, Te, Se, Eu, Hf, Tb, Al, Be, U, Ge, Pb, Ag, Cu, Gd, Au, Ca, Ba, La, and Fe, indicating that these 20 elements have a higher contribution to the grouping.

Reports indicate that using electron probe technology to study realgar can quickly determine whether it contains the toxic component arsenic trioxide (As₂O₃) (Xu et al., 2014). The results of the electron probe surface scan show that in most of realgar samples, the content of As and S elements is high. This indicates that the main mineral phase in both natural and artificially optimized realgar is realgar. In natural realgar, the distribution of Fe, Na, and Mg elements is minimal, falling below the detection limit (See Figure 4C; Table 5). Based on the micro-area data, the points where both As and S elements are distributed are identified as realgar. Points where Ca is distributed alone and its content is close to the total element content are identified as calcite, while points where Si is distributed alone and has a high content are identified as quartz. In the S3 sample of artificially optimized realgar, the Ca element content is relatively high, but the XRD detection results show a single realgar phase. This discrepancy may be due to the differences in detection limits between XRD and EMPA; EMPA tests the mineral phase and element composition in specific areas. Additionally, in the S3 sample, 7 points were selected, with some points showing Ca element content as high as 90%, leading to an elevated average value.

**TABLE 5:** Main elements content of natural and synthetics realgar (%).

Elements	As	Si	Fe	Na	S	Zn	Al	Pb	Ti	Mg	Ca
N1	34.87	0.03	—	—	65.01	0.02	—	0.02	0.01	—	0.06
N2	28.06	0.03	—	—	71.84	0.01	—	0.02	0.01	—	0.03
N3	46.11	0.03	—	—	53.82	0.00	0.00	0.02	0.00	—	0.02
N4	45.59	0.06	—	—	54.29	0.01	0.01	0.03	0.00	—	0.01
N5	47.96	0.04	—	—	51.94	0.00	0.02	0.03	0.00	—	0.01
N6	46.39	0.07	—	—	53.51	0.00	0.00	0.01	0.00	—	0.02
N7	41.50	0.04	—	—	58.40	0.01	0.01	0.02	0.00	—	0.02
N8	41.50	0.04	—	—	58.40	0.01	0.01	0.02	0.00	—	0.02
N9	41.50	0.04	—	—	58.40	0.01	0.01	0.02	0.00	—	0.02
N10	42.60	0.04	—	—	57.30	0.01	0.01	0.02	0.00	—	0.02
N11	45.51	0.05	—	—	54.39	0.01	0.01	0.02	0.00	—	0.02
N12	45.36	0.05	—	—	54.54	0.01	0.01	0.02	0.00	—	0.02
N13	45.28	0.05	—	—	54.62	0.01	0.01	0.02	0.00	—	0.02
S1	33.54	0.02	—	—	66.39	0.00	0.00	0.03	0.01	—	0.00
S2	39.37	2.54	—	—	57.84	0.01	0.01	0.03	0.01	—	0.19
S3	17.32	0.04	—	—	45.17	0.00	0.00	0.02	0.00	0.11	37.33
S4	49.05	0.01	—	—	50.86	0.01	0.02	0.03	0.00	—	0.01
S5	46.58	0.01	—	—	53.38	0.01	—	0.02	0.00	—	0.01
S6	43.77	0.02	—	—	56.17	0.00	—	0.02	0.00	—	0.01
S7	51.77	0.25	—	—	47.79	0.01	0.06	0.03	0.01	—	0.09
S8	47.51	0.02	0.01	—	52.42	0.01	—	0.02	0.01	—	0.02
S9	44.71	0.02	0.01	—	55.22	0.01	—	0.02	0.00	—	0.02
S10	46.03	0.02	0.01	—	53.88	0.01	0.00	0.03	0.00	—	0.01
S11	45.99	0.02	0.01	0.01	53.95	0.01	—	0.03	0.01	—	0.00

### 3.5 Toxicity study of natural and artificially optimized realgar

#### 3.5.1 Testing arsenic dissolution amount in artificial environment

Realgar primarily consists of tetraarsenic tetrasulfide (As₄S₄). As early as 1981, the International Health Organization classified arsenic as a human carcinogen (Hu, 1985), and the list of toxic traditional Chinese medicines issued by the State Council in 1988 explicitly included realgar (Jia, 2019; Zhang, 1988). As₄S₄ is insoluble in water and is not easily absorbed by the human body to exert its medicinal effects. Current research indicates that the medicinal mechanism of realgar is mainly due to its soluble components binding with the thiol groups on proteins or small molecules in the body (Parekh, 2021). Therefore, examining the dissolution characteristics of arsenic in gastrointestinal fluids is crucial for assessing the toxicity and efficacy of realgar. Therefore, we replicated the dissolution environment of heavy metal arsenic in human gastrointestinal fluids. The dissolution components of both artificially optimized and natural realgar were identified to seek out differential components.

The average arsenic dissolution amount in artificial intestinal solution for the six batches of natural realgar was 1326.11 mg·kg^−1^, which is 4.16% higher compared to the six batches of artificially optimized realgar. In artificial gastric solution, the average arsenic dissolution amount for the six batches of natural realgar was 2476.78 mg·kg^−1^, 43.85% higher than that of the artificially optimized realgar. In 0.16% hydrochloric acid, the average arsenic dissolution amount for the six batches of natural realgar was 2260.54 mg·kg^−1^, 16.63% higher than the artificially optimized realgar. The results of arsenic valence state determination in the 18 batches of realgar samples showed that As (III) was predominant. The average dissolution amount of As (III) in artificial gastric and intestinal solutions and 0.16% hydrochloric acid for the six batches of natural realgar was 19 times, 20 times, and 7 times that of As (V), respectively. For the six batches of artificially optimized realgar, the average dissolution amount of As (III) in artificial gastric and intestinal solutions and 0.16% hydrochloric acid was 95 times, 24 times, and 6 times that of As (V), respectively. Specific results are shown in Table 6.

**TABLE 6:** Soluble arsenic content in natural realgar, artificial optimized realgar and its processed products.

Sample number	Method	Soluble arsenic content/mg·kg^−1^	As (V) content/mg·kg^−1^	As (III) content/mg·kg^−1^
N1	Artificial intestinal solution	1711.04	391.56	10424.52
N2	Artificial intestinal solution	1549.21	333.97	2543.42
N4	Artificial intestinal solution	1660.10	142.51	3541.92
N8	Artificial intestinal solution	758.09	72.58	2645.56
N9	Artificial intestinal solution	1714.65	48.26	1115.40
N10	Artificial intestinal solution	563.59	37.47	995.54
S1	Artificial intestinal solution	1162.44	79.06	1640.02
S4	Artificial intestinal solution	1204.99	80.41	1857.79
S7	Artificial intestinal solution	1657.25	132.62	3182.09
S8	Artificial intestinal solution	1442.80	76.96	2191.14
S9	Artificial intestinal solution	1138.28	48.95	1695.07
S10	Artificial intestinal solution	1020.18	109.25	2915.50
Y2	Artificial intestinal solution	5681.56	105.77	4659.66
Y3	Artificial intestinal solution	2947.59	43.25	2239.92
Y4	Artificial intestinal solution	4288.91	76.58	3380.71
N1	Artificial gastric solution	1436.83	0.00	1329.66
N2	Artificial gastric solution	3823.67	320.80	2260.08
N4	Artificial gastric solution	3299.38	45.07	2403.35
N8	Artificial gastric solution	2880.15	25.65	555.07
N9	Artificial gastric solution	1781.79	22.33	1060.42
N10	Artificial gastric solution	1638.85	23.84	1021.27
S1	Artificial gastric solution	1279.20	21.50	1002.66
S4	Artificial gastric solution	1633.49	24.16	1261.95
S7	Artificial gastric solution	1051.18	0.00	1746.22
S8	Artificial gastric solution	1567.34	0.00	2889.04
S9	Artificial gastric solution	1334.04	24.58	913.43
S10	Artificial gastric solution	1479.43	24.08	1178.38
Y2	Artificial gastric solution	3648.90	119.24	3248.82
Y3	Artificial gastric solution	2663.84	40.13	2181.91
Y4	Artificial gastric solution	3129.87	61.41	2399.28
N1	0.16% hydrochloric acid	2332.01	265.69	5682.32
N2	0.16% hydrochloric acid	2312.62	589.92	1234.15
N4	0.16% hydrochloric acid	2833.62	265.65	1802.05
N8	0.16% hydrochloric acid	1446.30	182.00	903.72
N9	0.16% hydrochloric acid	2413.91	198.25	1803.44
N10	0.16% hydrochloric acid	2224.75	339.59	1549.87
S1	0.16% hydrochloric acid	2257.11	343.16	1801.63
S4	0.16% hydrochloric acid	1814.78	251.93	1373.38
S7	0.16% hydrochloric acid	1528.67	234.34	1196.15
S8	0.16% hydrochloric acid	2131.33	229.32	1941.78
S9	0.16% hydrochloric acid	1678.76	254.85	1368.86
S10	0.16% hydrochloric acid	1897.33	183.34	1407.91
Y2	0.16% hydrochloric acid	1941.65	159.47	9828.36
Y3	0.16% hydrochloric acid	4585.09	91.31	4706.39
Y4	0.16% hydrochloric acid	6326.31	153.02	6553.61

#### 3.5.2 The effects of realgar and artificially optimized realgar on mice weight and organs

To further investigate the toxicity of realgar, KM mice were selected and randomly divided into a control group, low, medium, and high dosage groups of natural realgar or artificially optimized realgar. The mice were administered natural realgar N1 and artificially optimized realgar S1 via gavage continuously for 28 days. The evaluation indicators included the levels of ALT, AST, and BUN in the serum of mice in different administration groups, histopathological damage to liver and kidney tissues, and arsenic species content in the liver and kidneys. These indicators were used to comprehensively assess the safety of artificially optimized realgar and natural realgar, providing relevant references for clinical rational use and realgar toxicity research.

**FIGURE 5:**
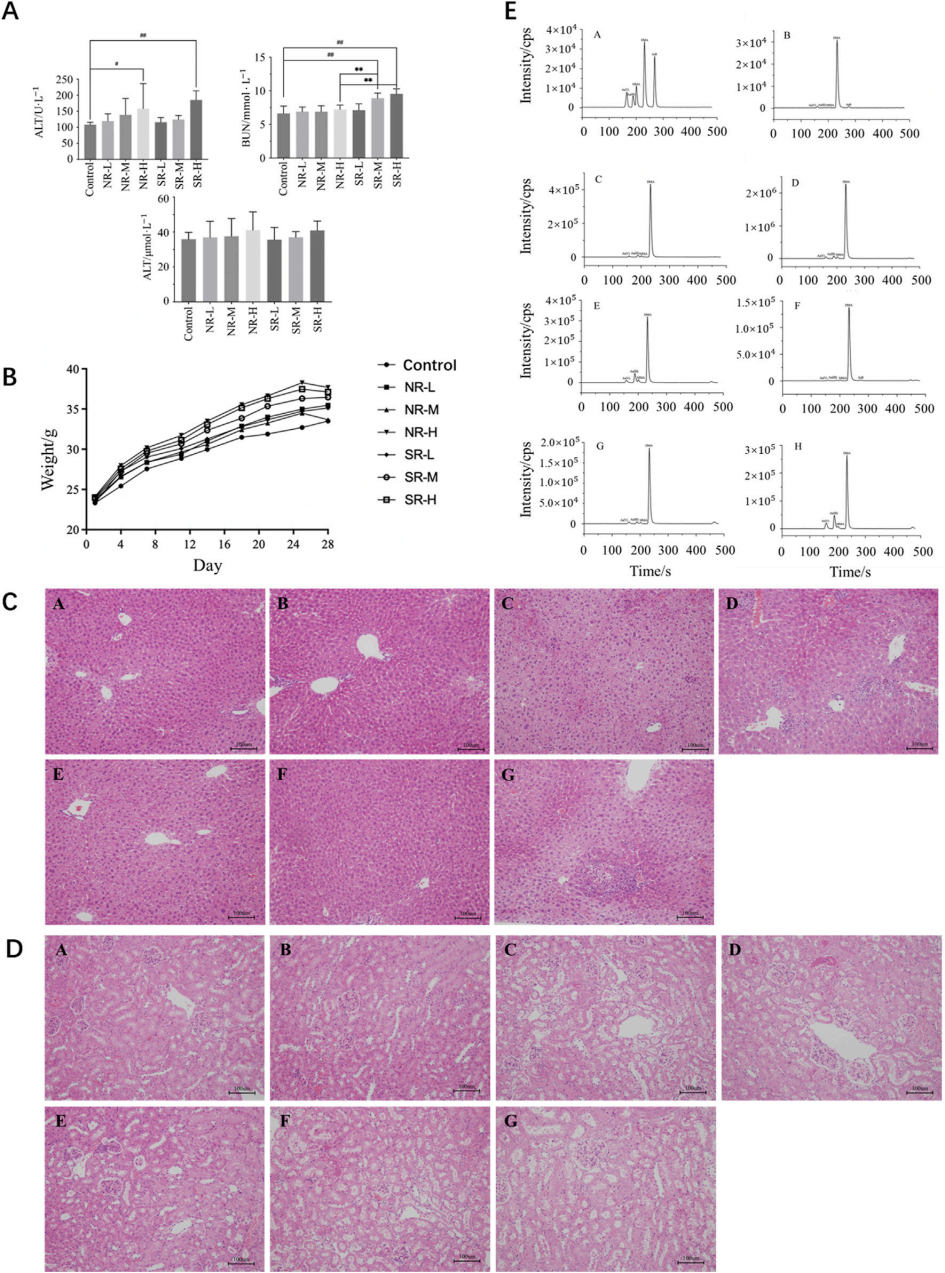
Toxicity of natural and artificially optimized realgar. **(A)** Serum levels of ALT, AST and BUN in each group of mice (n = 6), ^#^ *P* < 0.05, ^##^ *P* < 0.01, **P < 0.01; **(B)** Changes in body weight of mice in each group (n = 10); **(C)** HE staining of mice liver tissue in each group(n = 6, × 200), **(A–G)** are control group, NR-L, NR-M, NR-H, SR-L, SR-M, and SR-H (100 μm); **(D)** HE staining of mice renal tissue in each group (n = 6, × 200) (100 μm); **(E)** Comparison of arsenic valence in urine of mice in each group (n = 3), **(A–H)** are standard group, control group, NR-L group, NR-M group, NR-H group, SR-L group, SR-M group and SR-H group.

Compared to the control group, serum ALT levels in the NR-H group of mice increased (*P* < 0.05), and in the SR-H group, serum ALT levels significantly increased (*P* < 0.01). The other dosing groups showed an upward trend, but there was no statistical difference compared to the control group (*P* > 0.05). Compared to the control group, BUN levels in all dosing groups showed an increasing trend, with the SR-M and SR-H groups having significantly higher BUN levels (*P* < 0.01). Furthermore, the BUN levels in the SR-M and SR-H groups were significantly higher than those in the corresponding natural realgar dosing groups at the same dosage (*P* < 0.01). The results are shown in Tables 7, 8 and Figure 5A. For the changes in body weight of mice over 28 days of treatment, compared to the control group, there were no significant differences in the weight gain rates among the groups (see Tables 9, 10; Figure 5B). The liver and kidney indices of the mice revealed that the NR-M, NR-H, SR-L, SR-M, and SR-H groups had higher indices for both liver and kidney compared to the control group, with NR-H showing a statistically significant difference (*P* < 0.05) (see Tables 11, 12).

**TABLE 7:** Serum ALT, AST and BUN contents in male mice groups (n = 5).

Group	ALT/U·L^−1^	AST/μmol·L^−1^	BUN/mmol·L^−1^
Control group	39.19 ± 3.23	136.46 ± 37.85	10.4 ± 2.07
NR-L group	32.06 ± 3.78	96.21 ± 14.34	8.6 ± 2.61
NR-M group	49.72 ± 21.01	137.47 ± 51.16	10.4 ± 3.29
NR-H group	32.45 ± 11.69	84.55 ± 12.91	8 ± 3.46
SR-L group	36.29 ± 10.05	95.53 ± 14.32	4.8 ± 1.3
SR-M group	39.39 ± 7.63	123.72 ± 27.58	6.4 ± 1.52^##^**
SR-H group	36.5 ± 7.34^##^	132.78 ± 25	6.6 ± 0.89^##^**

Note: Compared with control group ^#^ *P* < 0.05, ^##^ *P* < 0.01, Compared with natural realgar at the same dose **P < 0.01.

**TABLE 8:** Serum ALT, AST and BUN contents in female mice groups (n = 5).

Group	ALT/U·L^−1^	AST/μmol·L^−1^	BUN/mmol·L^−1^
Control group	40.79 ± 10.1	134.32 ± 63.2	7.72 ± 1.05
NR-L group	42.93 ± 23.22	121.29 ± 15.99	7.66 ± 0.63
NR-M group	35.68 ± 13.92	118.91 ± 25.12	7.19 ± 1.37
NR-H group	58.06 ± 32.76	183.41 ± 77.01	8.75 ± 1.88
SR-L group	85.5 ± 114.85	166.44 ± 84.32	6.57 ± 0.91
SR-M group	38.08 ± 16.81	135.61 ± 16.83	7.72 ± 1.61
SR-H group	59.76 ± 56.31^##^	172.59 ± 81.38	9.31 ± 3.49

Note: Compared with control group ^#^ *P* < 0.05, ^##^ *P* < 0.01, Compared with natural realgar at the same dose **P < 0.01.

**TABLE 9:** Weight change of male mice in each group (n = 5).

Days	Control	NR-L	NR-M	NR-H	SR-L	SR-M	SR-H
Day 1	24.3 ± 1.23	24.84 ± 1.35	24.82 ± 2.38	25.52 ± 1.26	24.68 ± 1.01	24.82 ± 1.11	25.16 ± 1.37
Day 4	28.08 ± 1.68	28.76 ± 2.43	28.14 ± 4.56	31.06 ± 1.12	29.64 ± 1.4	28.64 ± 3.16	29.98 ± 2.77
Day 7	29.94 ± 2.24	31 ± 3.07	31.02 ± 5.76	34.16 ± 1.37	32.48 ± 1.46	31.92 ± 3.84	32.7 ± 3.32
Day 11	31.92 ± 2.81	33.24 ± 3.18	32.82 ± 6.28	37.24 ± 1.78	33.88 ± 1.68	33.42 ± 3.06	35 ± 4.13
Day 14	33.56 ± 3.25	35.74 ± 3.49	34.12 ± 6.67	39.78 ± 2.43	36.12 ± 2.19	35.42 ± 3.17	36.86 ± 5.31
Day 18	35.38 ± 3.73	38.06 ± 3.84	35.94 ± 7.28	42.56 ± 2.66	38.44 ± 2.21	37.64 ± 3.5	39.68 ± 5.96
Day 21	36.02 ± 3.51	39.58 ± 3.81	37.02 ± 7.76	44.12 ± 2.88	39.62 ± 2.01	39.38 ± 3.66	40.82 ± 6.22
Day 25	36.15 ± 3.78	40.62 ± 3.51	38.3 ± 8.21	45.88 ± 3	41.04 ± 1.73	41.04 ± 4	42.72 ± 6.64
Day 28	24.3 ± 1.23	24.84 ± 1.35	24.82 ± 2.38	25.52 ± 1.26	24.68 ± 1.01	24.82 ± 1.11	25.16 ± 1.37

**TABLE 10:** Weight change of female mice in each group (n = 5).

Days	Control	NR-L	NR-M	NR-H	SR-L	SR-M	SR-H
Day 1	22.36 ± 1.77	22.24 ± 2.19	22.66 ± 1.73	22.74 ± 2.08	22.08 ± 1.56	22.76 ± 1.48	22.84 ± 2.01
Day 4	22.76 ± 3.05	24.36 ± 2.76	25.16 ± 2.94	24.88 ± 2.65	24.94 ± 2.65	25.68 ± 1.81	25.18 ± 3.52
Day 7	25.152 ± 4.19	25.74 ± 4.37	25.74 ± 3.16	26.18 ± 2.98	25.58 ± 3.38	27.34 ± 2.63	26.84 ± 4.36
Day 11	25.78 ± 4.15	25.56 ± 3.18	26.5 ± 3.11	26.22 ± 3.24	26.3 ± 3.89	27.98 ± 1.97	27.3 ± 4.62
Day 14	26.38 ± 4.39	26.3 ± 3.94	27.1 ± 3.58	27.22 ± 3.81	26.42 ± 4.16	28.5 ± 2.04	29.28 ± 5.96
Day 18	27.6 ± 4.34	27.68 ± 3.95	28.92 ± 4.13	28.52 ± 4.41	27.18 ± 3.72	29.56 ± 2.25	30.62 ± 5.85
Day 21	27.78 ± 4.66	28.4 ± 3.92	29.5 ± 4.12	29.18 ± 4.24	27.74 ± 3.43	31.26 ± 2.57	31.76 ± 5.94
Day 25	28.42 ± 4.92	29.36 ± 4.38	30.68 ± 4.53	30.68 ± 4.79	28.4 ± 3.71	32.04 ± 2.73	32.22 ± 5.81
Day 28	28.64 ± 4.68	28.96 ± 3.65	29.1 ± 4.17	29.2 ± 3.74	28.34 ± 3.58	31.6 ± 3.52	31.02 ± 6.1

**TABLE 11:** Kidney and liver organ indices of male mice in each group (n = 5).

Group	Liver index/%	Kidney index/%
Control group	3.74 ± 0.486	1.50 ± 0.171
NR-L group	3.70 ± 0.227	1.55 ± 0.147
NR-M group	3.81 ± 0.123	1.48 ± 0.095
NR-H group	3.62 ± 0.240	1.47 ± 0.191
SR-L group	3.92 ± 0.290	1.55 ± 0.097
SR-M group	3.74 ± 0.250	1.51 ± 0.142
SR-H group	3.74 ± 0.080	1.40 ± 0.11

Note: Compared with control group ^#^ *P* < 0.05.

**TABLE 12:** Kidney and liver organ indices of female mice in each group (n = 5).

Group	Liver index/%	Kidney index/%
Control group	3.65 ± 0.27	1.27 ± 0.17
NR-L group	3.75 ± 0.53	1.23 ± 0.14
NR-M group	3.73 ± 0.16	1.29 ± 0.28
NR-H group	4.27 ± 0.46	1.36 ± 0.11
SR-L group	4.19 ± 0.61	1.25 ± 0.08
SR-M group	4.17 ± 0.29	1.26 ± 0.11
SR-H group	4.03 ± 0.62	1.4 ± 0.21

Note: Compared with control group ^#^ *P* < 0.05.

#### 3.5.3 HE staining and injury scoring

In the control group, the liver lobular structure was clear, with hepatocytes arranged radially around the central vein. No signs of degeneration, necrosis, or inflammatory cell infiltration in the stroma were observed. In the NR-L group, mild hepatocyte hypertrophy was noted in a few mice (1/6), with slight infiltration of inflammatory cells in the portal areas and mild proliferation of Kupffer cells. The liver tissue structure appeared normal in the remaining mice, with no significant lesions. In the NR-M group, some mice showed diffuse hepatocyte hypertrophy (3/6), with mild or moderate necrosis and neutrophil infiltration (2/6). A few mice also had mild proliferation of Kupffer cells (1/6), indicating a trend toward more severe conditions compared to the control group (*P* < 0.1). In the NR-H group, most mice exhibited mild hepatocyte hypertrophy (5/6), showing a significant difference from the control group (*P* < 0.01). Some mice had mild or moderate proliferation of Kupffer cells (3/6), indicating a trend toward worsening compared to the control group (*P* < 0.1). A few mice had moderate necrosis and mixed inflammatory cell infiltration in hepatocytes (1/6), and mild infiltration of inflammatory cells in the parenchyma/portal areas (1/6), with differences not being statistically significant compared to the control group.

In the SR-L group, occasional mice showed focal hepatocyte necrosis and mixed cell infiltration (1/6), while the remaining animals had normal liver tissue structure with no significant lesions. In the SR-M group, some mice had mild hepatocyte necrosis and neutrophil infiltration (1/6), mild hepatocyte hypertrophy (1/6), and slight infiltration of inflammatory cells in the parenchyma/portal areas (2/6). The liver tissue structure was normal in the remaining mice, with no significant lesions. In the SR-H group, most mice exhibited mild hepatocyte hypertrophy (5/6), and some had mild necrosis and neutrophil or mixed inflammatory cell infiltration (3/6), showing significant differences compared to the control group (*P* < 0.01 and *P* < 0.05). Some mice also had mild proliferation of Kupffer cells (2/6), indicating a trend toward worsening compared to the control group (*P* < 0.1). Specific results are detailed in Figure 5C; Table 13.

**TABLE 13:** Statistical table of liver histological changes of mice in each group (n = 6).

Group	Hepatocyte hypertrophy	Hepatocyte necrosis	Proliferation of kupffer cells	Inflammatory cells infiltration
Control group	17.50	21.00	23.00	21.92
NR-L group	25.33	21.00	28.50	23.33
NR-M group	31.75	34.17	27.83	31.17
NR-H group	41.25^##^	21.00	37.17	27.83
SR-L group	17.50	25.08	23.50	21.92
SR-M group	20.58	26.00	23.50	31.17
SR-H group	39.58^##^	34.17	23.50	40.42^#^

Note: Compared with control group ^#^ *P* < 0.05, ^##^ *P* < 0.01.

In the control group, no swelling, degeneration, or necrosis of renal tubular epithelial cells was observed, and there was no inflammatory cell infiltration in the stroma. The tissue structure appeared normal. In the NR-L group, renal tubular epithelial cells showed no signs of degeneration or necrosis, and there was no inflammatory cell infiltration in the stroma. The findings were comparable to the control group. In the NR-M group, some mice exhibited mild degeneration of renal tubules (3/6), which was significantly different from the control group (*P* < 0.05). Mild protein casts were also present in the tubular lumens (3/6). The remaining mice had normal kidney tissue structure with no significant lesions. In the NR-H group, some mice (2/6) showed mild degeneration of renal tubules and mild protein casts in the lumens. The rest of the mice had normal kidney tissue structure with no significant lesions.

In the SR-L group, a few mice displayed mild chronic progressive nephropathy (1/6), while the remaining mice had normal kidney tissue structure with no significant lesions. In the SR-M group, some mice exhibited mild degeneration of renal tubules (4/6), showing a significant difference from the control group (*P* < 0.05). Mild protein casts were also observed in the tubular lumens (3/6). A few mice showed mild chronic progressive nephropathy (1/6), with the remaining mice having normal kidney tissue structure and no significant lesions. In the SR-H group, all mice showed mild degeneration of renal tubules (6/6), with mild protein casts in the lumens (4/6). These findings were significantly different from the control group (*P* < 0.01 and *P* < 0.05). The remaining mice had normal kidney tissue structure with no significant lesions. Detailed results are provided in Figure 5D; Table 14.

**TABLE 14:** Statistical table of renal histological changes of mice in each group (n = 6).

Group	Degeneration of renal tubules	Protein casts
Control group	16.50	18.50
NR-L group	16.50	18.50
NR-M group	26.50^#^	31.50
NR-H group	23.50	27.17
SR-L group	16.50	18.50
SR-M group	39.17^#^	33.00
SR-H group	42.50^##^	37.33^#^

Note: Compared with control group ^#^ *P* < 0.05, ^##^ *P* < 0.01.

#### 3.5.4 Total arsenic content in liver, kidney, serum and urine

Arsenic was detectable in serum, liver, and kidney tissues across all dosage groups, with the highest concentration found in the blood, followed by the kidneys, and the lowest in the liver (see Tables 15, 16). Compared to the control group, the NR-M, NR-H, SR-M, and SR-H groups exhibited a significant increase in arsenic levels in both blood and liver (*P* < 0.01). Among these, the SR-H group showed a notable increase in arsenic levels in both blood and liver compared to the same dose of natural realgar (*P* < 0.01). Additionally, the NR-H and SR-H groups showed a significant rise in arsenic levels in kidney tissues compared to the control group (*P* < 0.01). These results indicate that high doses of realgar lead to arsenic accumulation in liver and kidney tissues, with higher arsenic levels observed in the blood, liver, and kidneys of the artificially optimized realgar groups compared to the natural realgar groups.

**TABLE 15:** Total arsenic content in liver and kidney of mice in each group (n = 5).

Group	As in liver/mg·kg^−1^	As in kidney/mg·kg^−1^
Control group	—	—
NR-L group	0.20 ± 0.02	0.15 ± 0.09
NR-M group	0.45 ± 0.13^##^	0.34 ± 0.08
NR-H group	0.68 ± 0.20^##^	1.28 ± 0.89^##^
SR-L group	0.20 ± 0.17	0.09 ± 0.05
SR-M group	0.74 ± 0 .26^##^	0.71 ± 0.39
SR-H group	1.45 ± 0.56^##^**	1.89 ± 1.40^##^

Note: Compared with control Group ^##^ *P* < 0.01, Compared with natural realgar at the same dose **P < 0.01.

**TABLE 16:** Total arsenic levels in serum of mice in each group (n = 3).

Group	As in blood/mg·kg^−1^
Control group	1.19 ± 0.32
NR-L group	9.39 ± 0.95
NR-M group	31.62 ± 6.89^##^
NR-H group	31.66 ± 10.23^##^
SR-L group	8.98 ± 6.43
SR-M group	24.60 ± 7.05^##^
SR-H group	76.26 ± 20.76^##^**

Note: Compared with control Group^##^ *P* < 0.01, Compared with natural realgar at the same dose **P < 0.01.

In the urine of all mouse groups, As (V), As (III), MMA, and DMA were detected, while AsB was found in the control and SR-L groups, as shown in Figure 5E. Compared to the control group, the four arsenic species in the urine of the other groups showed a dose-dependent relationship. In the NR-H and SR-H groups, the levels of various arsenic species significantly increased (*P* < 0.01). Compared to the NR-H group, the SR-H group showed increased levels of pentavalent and trivalent arsenic (*P* < 0.05) and a significant decrease in DMA levels (*P* < 0.01). Compared to the control group, the NR-L group had significantly increased levels of MMA and DMA (*P* < 0.01), the NR-M group had significantly elevated levels of pentavalent arsenic, MMA, and DMA (*P* < 0.01), and the SR-M group had significantly increased levels of MMA and DMA (*P* < 0.01), with levels lower than those in the NR-M group (*P* < 0.05), as shown in Table 17. These results suggest that the main form of arsenic in the urine of mice metabolized from realgar is DMA, and high doses of realgar can easily lead to chronic arsenic poisoning.

**TABLE 17:** Urinary valence arsenic levels in mice by group (n = 3).

Group	As (V)/μg·L^−1^	As (III)/μg·L^−1^	MMA/μg·L^−1^	DMA/μg·L^−1^
Control group	4.10 ± 0.64	1.58 ± 0.65	1.54 ± 0.38	209.15 ± 41.80
NR-L group	228.40 ± 25.10	88.57 ± 91.45	231.81 ± 28.16^##^	13846.53 ± 2335.76^##^
NR-M group	417.49 ± 101.96^##^	700.64 ± 207.35	504.73 ± 72.39^##^	22449.34 ± 4194.67^##^
NR-H group	713.87 ± 268.61^##^	2625.43 ± 1216.56^##^	670.24 ± 29.29^##^	23448.33 ± 2031.63^##^
SR-L group	7.33 ± 1.44	12.09 ± 2.53	7.56 ± 1.06**	911.80 ± 264.02**
SR-M group	153.11 ± 25.80	265.81 ± 145.17	168.13 ± 140.72^#^**	7381.63 ± 346.24^##^**
SR-H group	1292.50 ± 455.29^##^*	4037.72 ± 1010.06^##^*	716.50 ± 114.65^##^	13855.35 ± 1357.97^##^**

Note: Compared with control Group ^#^ *P* < 0.05, ^##^ *P* < 0.01, Compared with natural realgar at the same dose *P < 0.05, **P < 0.01.

## 4 Discussion

Realgar, a traditional Chinese medicine with a history of use spanning thousands of years, has been found to be effective in treating malignant diseases through extensive pharmacological studies. However, historical unregulated mining of realgar resources and a lack of ecological awareness have led to environmental pollution around realgar mines, posing health risks to nearby residents. Consequently, realgar mining remains prohibited. The medicinal market has seen the emergence of artificially optimized realgar. There are several ways being proposed to generate artificially optimized realgar. One of them involves first crushing the tailing realgar deposit, then pulp preparation and flotation. Chemical impurity removal is carried out under the conditions of 45°C, 2 mol·L^−1^ hydrochloric acid concentration, a liquid-to-solid ratio of 2.5:1, a reaction time of 90 min, and a grinding time of 30 min. After impurity removal, the solution is filtered, and the filtered product is ground and vacuum-dried to obtain medicinal realgar. This method has been granted a patent (Sun, 2005; Hao, 2016), indicating its feasibility for large-scale industrial production. Therefore, we speculate that the artificially optimized realgar sold on the market may have been prepared using similar method.

Artificially optimized realgar differs significantly in properties from natural realgar. However, there are few studies on this topic. Therefore, it is crucial to identify distinctive features of both natural and artificially optimized realgar for quality control (Baláž and Sedlák, 2010). Additionally, adverse reactions to realgar and its compound formulations have raised safety concerns. Regulatory bodies in Sweden and the United Kingdom have reported high arsenic levels in Niu Huang Jie Du Pian, warning the public against its use. The US FDA has also banned its import. Therefore, a comprehensive safety evaluation of synthetic realgar is necessary. This study focuses on identifying features and evaluating the safety of natural and artificially optimized realgar, summarized as follows:

### 4.1 Identification features

Natural realgar stones and artificially optimized realgar stones exhibit significant differences in appearance, making them easily distinguishable. However, realgar is commonly used in powdered form, making it difficult to distinguish between natural and artificially optimized realgar powders (Jakubikova et al., 2013). In this study, microscopic identification combined with XRD technology and Raman spectroscopy effectively differentiates natural and artificially optimized realgar. In terms of microscopic identification, both natural realgar and artificially optimized realgar appear as orange-yellow crystals under a polarized light microscope, but natural realgar contains more white crystals. XRD results show natural realgar primarily consists of realgar, with some samples containing three impurities, consistent with microscopic identification. Artificially optimized realgar mostly consists of pararealgar, with most samples containing only one impurity. In terms of Raman spectral peaks, natural realgar has strong characteristic absorption peaks at 356, 345, 221, 194, 185, 171, and 146 cm^−1^, while artificially optimized realgar peaks at 362, 345, 273, 217, 186, 166, and 146 cm^−1^. Therefore, microscopic identification combined with XRD technology and Raman spectroscopy provides a quick and efficient way to distinguish the natural realgar and artificially optimized realgar. Additionally, by using PCA combined with Raman spectroscopy technology and integrating XRD analysis results, it is possible to generate a predictive model to identify natural realgar medicinal materials and artificially optimized realgar. However, due to the limited sample size collected in this study, the accuracy of the predictive model cannot be guaranteed. Nevertheless, the results indicate that developing a system to differentiate between natural and artificially optimized realgar has a certain level of feasibility.

### 4.2 Inorganic element content

Realgar is rich in various inorganic elements, some of which are involved in metabolic processes and biological effects while others have toxic effects. Studies have shown that elements, especially heavy metal, can interact with proteins and disrupt their functions. For example, calcium and cadmium have been shown to interact with estrogen receptor and androgen receptor and disrupt downstream signaling pathways (Psaltis et al., 2024; Cyrus et al., 2021; Sharawi et al., 2023). Using ICP-MS and electron probe technology, the content and distribution of inorganic elements in natural and artificially optimized realgar were measured. Natural realgar showed higher average levels of common elements like Ca, Mg, K, Na, and P, and lower levels of harmful elements like Pb. OPLS-DA analysis identified six differential components (Se, Eu, Tb, Al, Ge, Gd) with VIP > 1 and *P* < 0.05. These different inorganic element content may affect the efficacy of artificially optimized realgar, which requires further investigations. Electron probe results indicated significant S element distribution in both natural and artificially optimized realgar but no As_2_O_3_. Electron probe was found to provide a faster and more feasible way to detect the presence of the toxic component arsenic trioxide, enabling a more effective quality assessment of realgar.

### 4.3 Soluble arsenic content

The forms of soluble arsenic can be classified into four main categories: inorganic arsenic, such as arsenite (AsO₃³^−^, III) and arsenate (AsO₄^3−^, V); small organic arsenic molecules, such as monomethylarsonic acid (MMA, V), monomethylarsonous acid (MMA, III), dimethylarsinic acid (DMA, V), dimethylarsinous acid (DMA, III), trimethylarsine oxide (TMAO), and tetramethylarsonium ion (TMAs⁺); organic arsenic compounds, such as arsenobetaine (AsB), trimethylarsine lactate, arsenocholine (AsC), arsenolipids (AsL), arsenosugars, dimethylarsinoyl ethanol, dimethylarsinoyl nucleosides, dimethylarsinoyl nucleoside sulfate esters, and O-phosphoryl trimethylarsinoyl lactate; arsenic-containing biological macromolecules, such as arsenic (III or V) complexes with transferrin or hemoglobin. Different forms of arsenic vary significantly in their absorption, distribution, metabolism, and toxicity in the body. Inorganic arsenic is more toxic than organic arsenic, and trivalent arsenic is more toxic than pentavalent arsenic. Therefore, the typical order of arsenic toxicity is: inorganic arsenic (III) > inorganic arsenic (V) > organic arsenic (III) > organic arsenic (V) (Chen et al., 2022; Liu et al., 2023). Soluble arsenic, both the active and toxic component of realgar, was measured in simulated gastric and intestinal fluids, and 0.16% hydrochloric acid. Results showed that trivalent arsenic had higher average solubility than pentavalent arsenic in all media, with no other arsenic species detected. Natural realgar had higher soluble arsenic content in all media compared to artificially optimized realgar. Safety evaluation experiments using KM mice involved continuous oral administration for 28 days, observing liver and kidney damage. Results indicated renal tubule degeneration and protein deposition in the SR-H group, with more severe damage compared to the NR-H group. As was also detected in control group suggesting that potential widespread of contamination of As. Arsenic accumulation in the liver and kidneys was significantly higher in the SR-H group. Urine analysis detected trivalent arsenic, pentavalent arsenic, monomethylarsinic acid, and dimethylarsinic acid in all groups, with higher trivalent and pentavalent arsenic levels in the NR-H group, suggesting that high-dose, long-term use of synthetic realgar can cause more severe liver and kidney damage than natural realgar. Therefore, whether artificially optimized realgar can replace natural realgar remains open to debate. In addition, no significant difference was found between female and male mice.

This study systematically analyzed the identification features of synthetic and natural realgar using modern technology, and preliminarily evaluated their safety *in vitro* and *in vivo*. However, some limitations remain. For example, we purchased the natural realgar and artificially optimized realgar from different resources and tried our best to ensure the label (natural realgar VS artificially optimized realgar) were correct. However, there is still a chance that labels from commercial market were mislabeled. Future studies should systematically analyze processed natural and synthetic realgar and conduct more in-depth toxicological research on the mechanisms of their toxicity differences.

## Data Availability

The original contributions presented in the study are included in the article/Supplementary Material, further inquiries can be directed to the corresponding author.

## Abbreviations

As, Arsenic; As (III), Arsenous acid^+^; As (V), Arsenic acid^+^; AsB, Arsenic betaine; ALT, Alanine transaminase; AST, Aspartate transaminase; ATO, Arsenic trioxide; BUN, Blood urea nitrogen; CMC-Na, Sodium carboxyl methyl cellulose; DMA, Dimethyl arsenic acid; EPMA, Electron probe microanalyzer; HE, Hematoxylin-eosin; HPLC-ICP-MS, High performance liquid chromatography–inductively coupled plasma mass spectrometry; ICP-MS, Inductively coupled plasma mass spectrometry; iAs, Inorganic arsenic; MMA, Monome thylarsenic acid; RIF, Realgar-indigonaturalis formula; TAs, Total arsenic; XRD, X-ray diffraction; MMA, Methylmalonic acidemia; DMA, Urinary dimethylamine.
