# Supplementary figures and images for: Characterization and comparation of toxicity between natural realgar and artificially optimized realgar
Source: Front Pharmacol. 2024 Oct 28;15:1476139. doi: 10.3389/fphar.2024.1476139 (PMC11550961; doi:10.3389/fphar.2024.1476139)

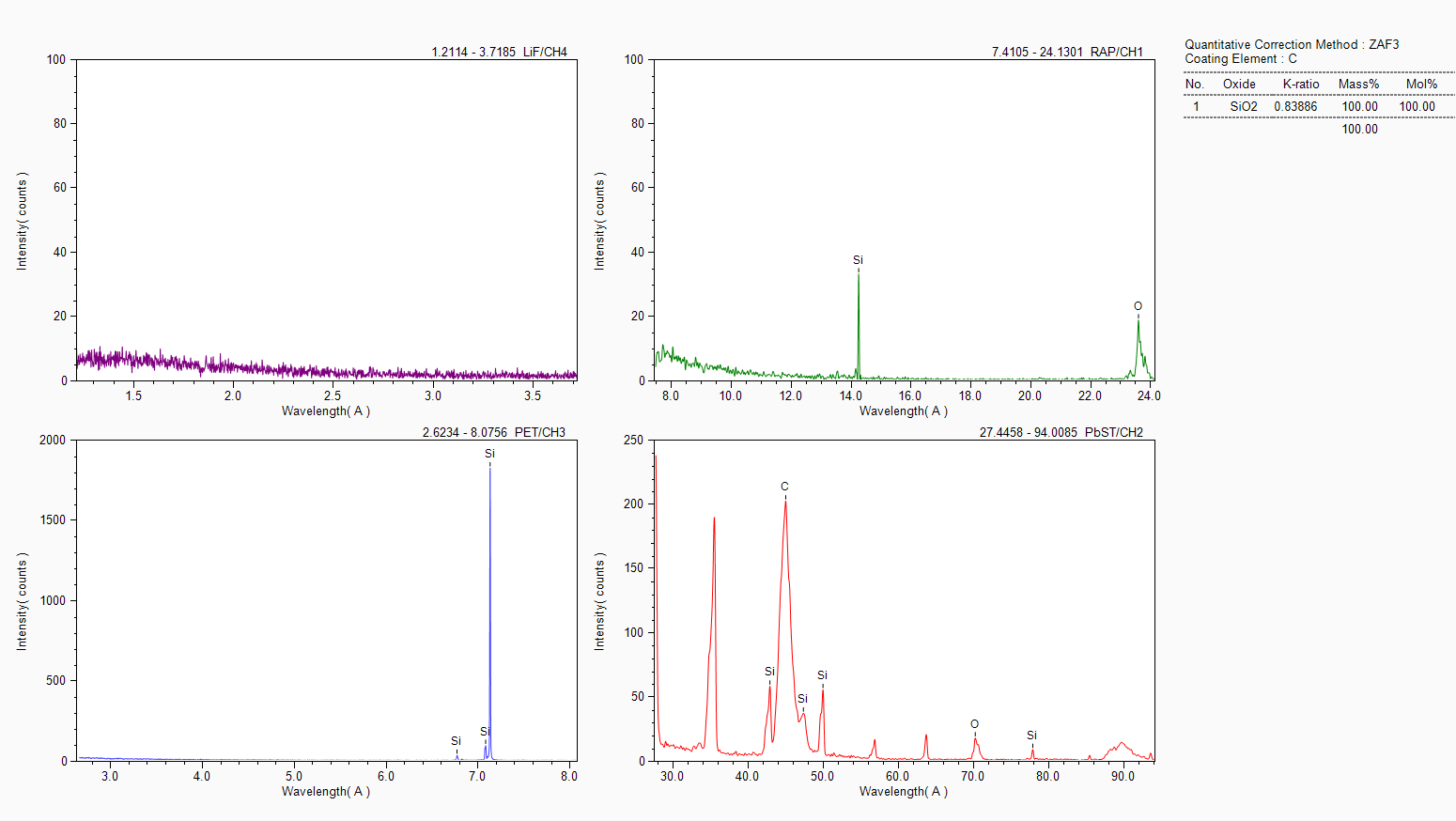

Supplement: Supplementary file 1 [file DataSheet2.zip › Electron Scans/xxy11-24/XH-24-2-1qual-1.bmp]

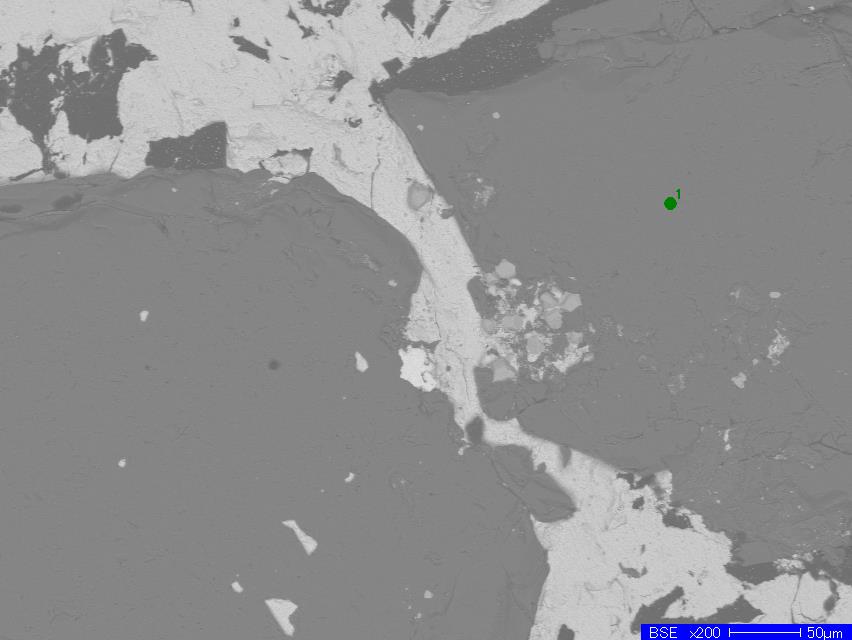

Supplement: Supplementary file 1 [file DataSheet2.zip › Electron Scans/xxy11-24/XH-13qual.jpeg]

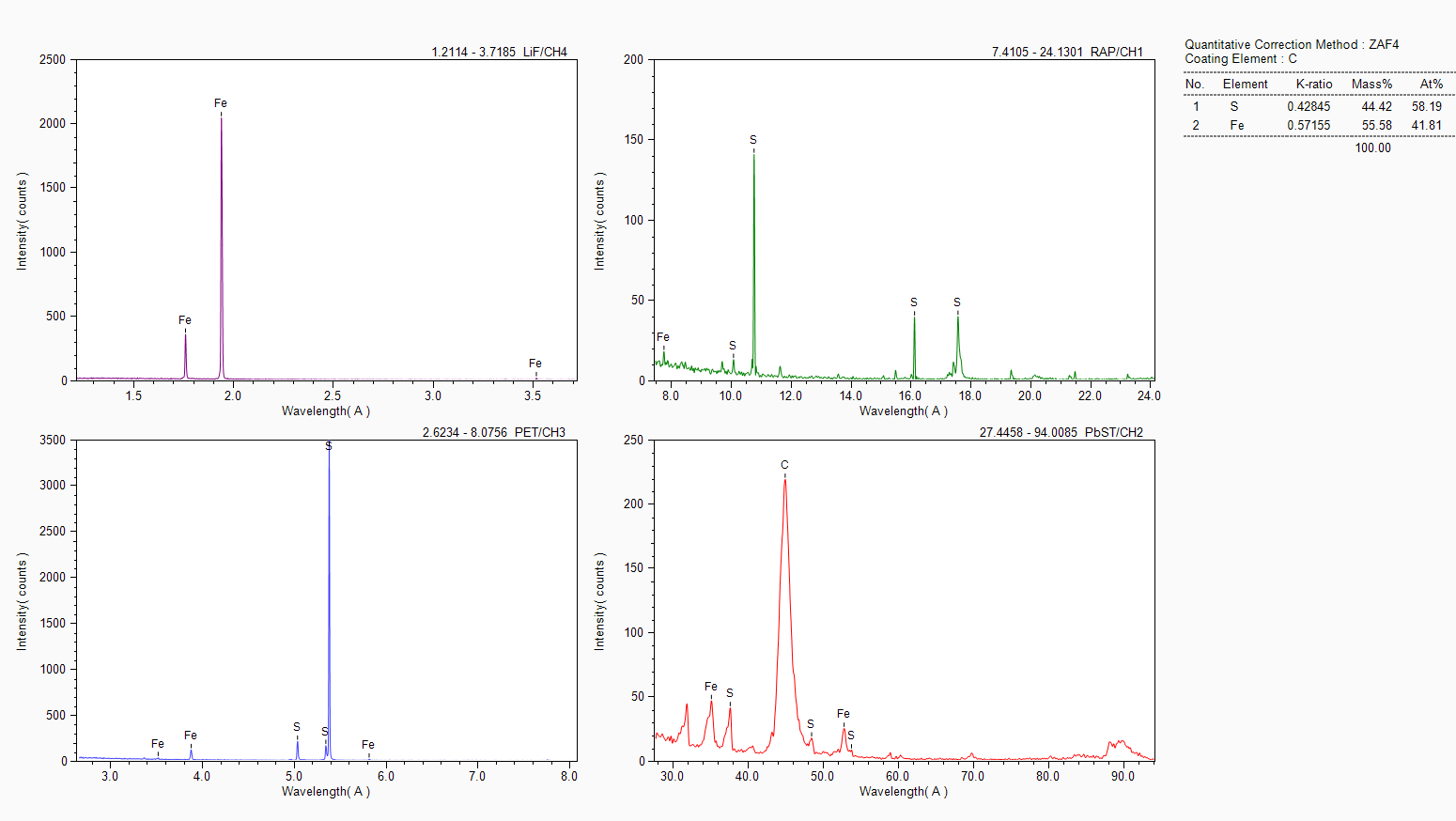

Supplement: Supplementary file 1 [file DataSheet2.zip › Electron Scans/xxy11-24/XH-24-2-1qual-2.bmp]

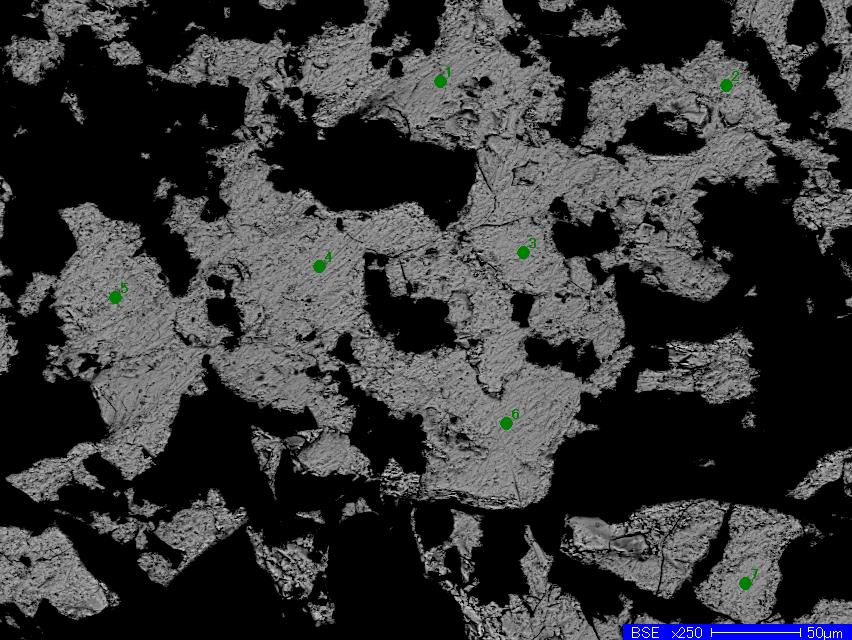

Supplement: Supplementary file 1 [file DataSheet2.zip › Electron Scans/xxy11-24/XH-15-2quant.jpeg]

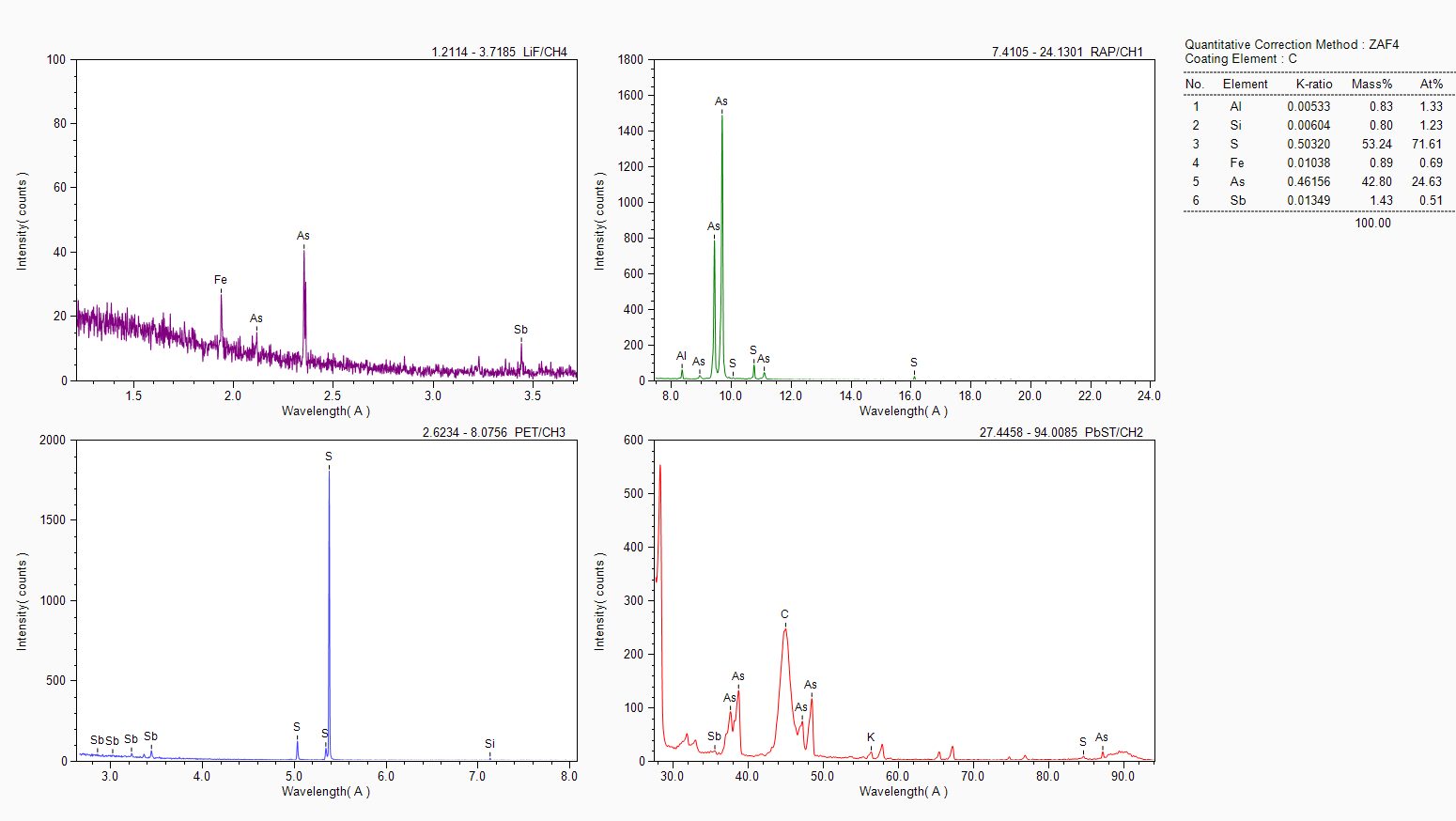

Supplement: Supplementary file 1 [file DataSheet2.zip › Electron Scans/xxy11-24/XH-24-2-1qual-3.bmp]

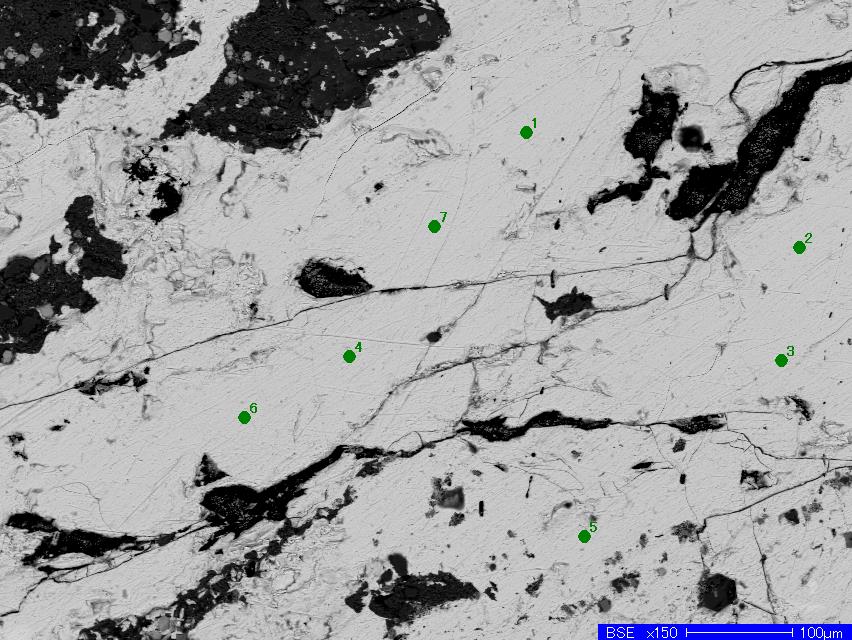

Supplement: Supplementary file 1 [file DataSheet2.zip › Electron Scans/xxy11-24/XH-11quant.jpeg]

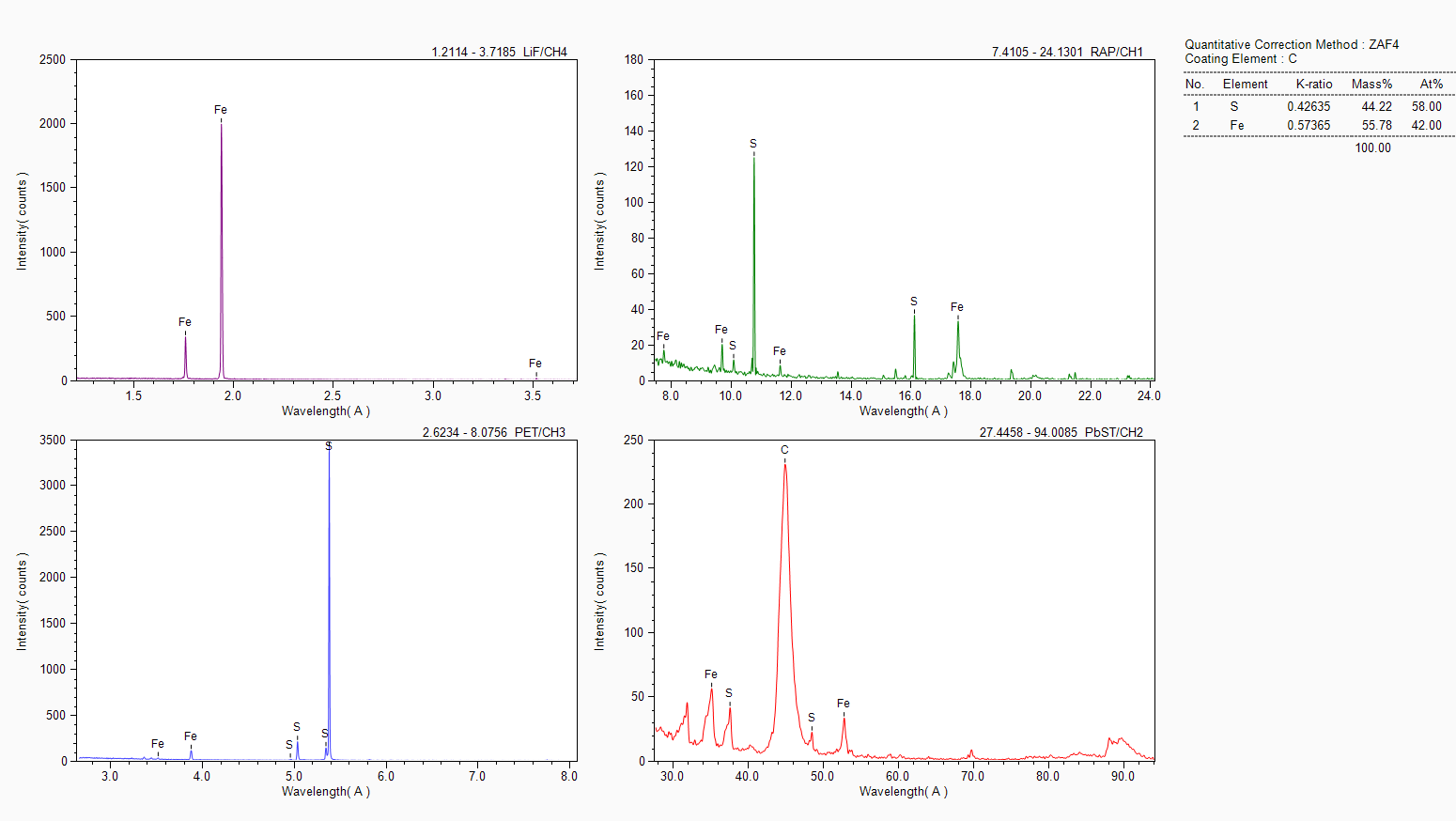

Supplement: Supplementary file 1 [file DataSheet2.zip › Electron Scans/xxy11-24/XH-24-2-1qual-4.bmp]

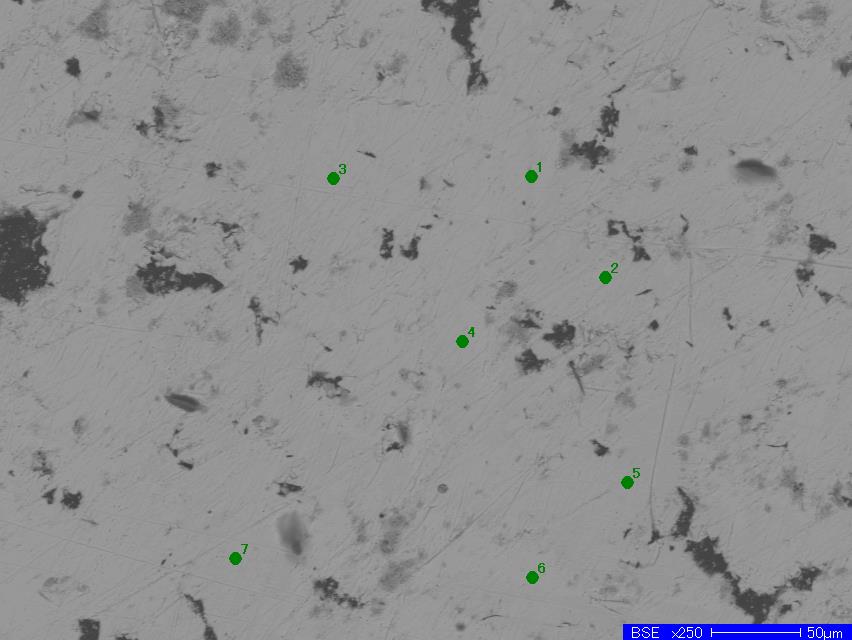

Supplement: Supplementary file 1 [file DataSheet2.zip › Electron Scans/xxy11-24/XH-17quant.jpeg]

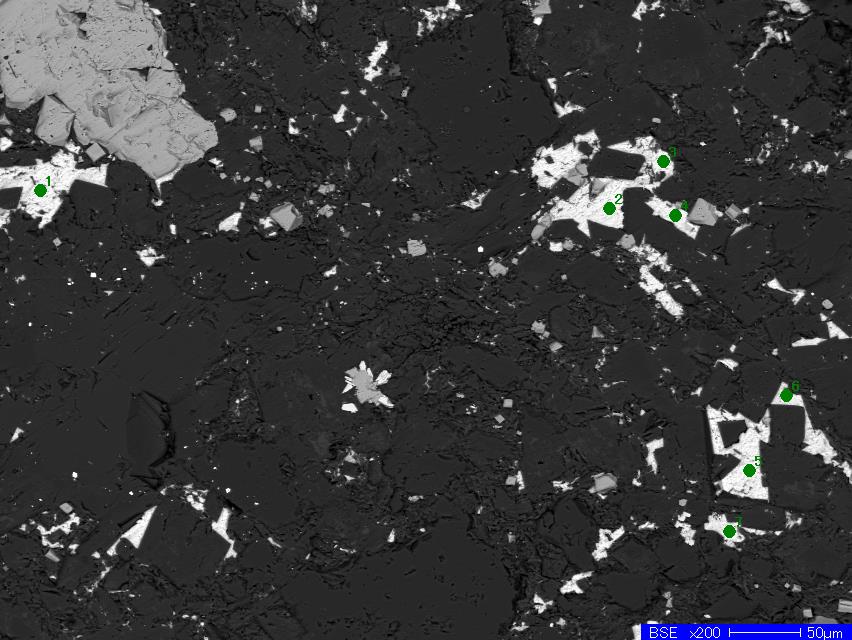

Supplement: Supplementary file 1 [file DataSheet2.zip › Electron Scans/xxy11-24/XH-24-2-1quant.jpeg]

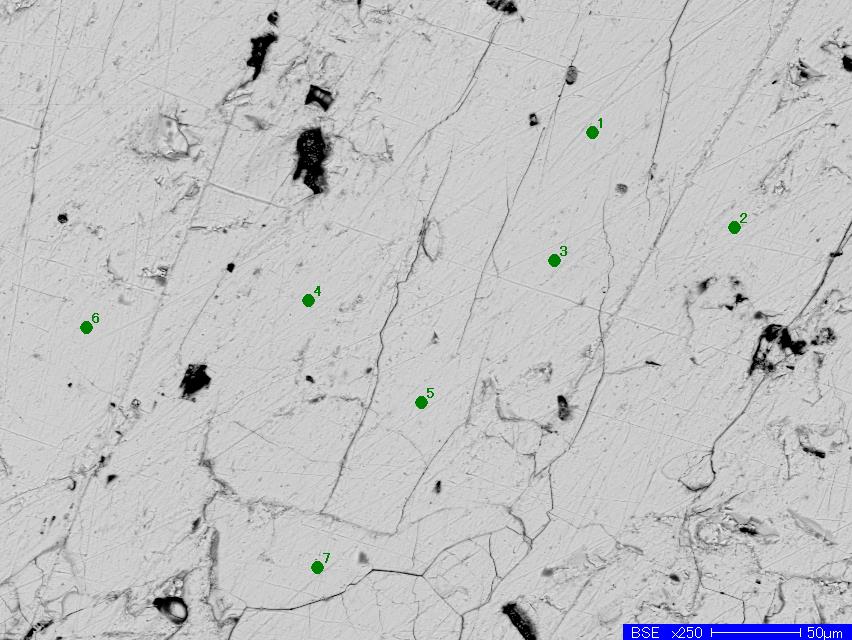

Supplement: Supplementary file 1 [file DataSheet2.zip › Electron Scans/xxy11-24/XH-22quant.jpeg]

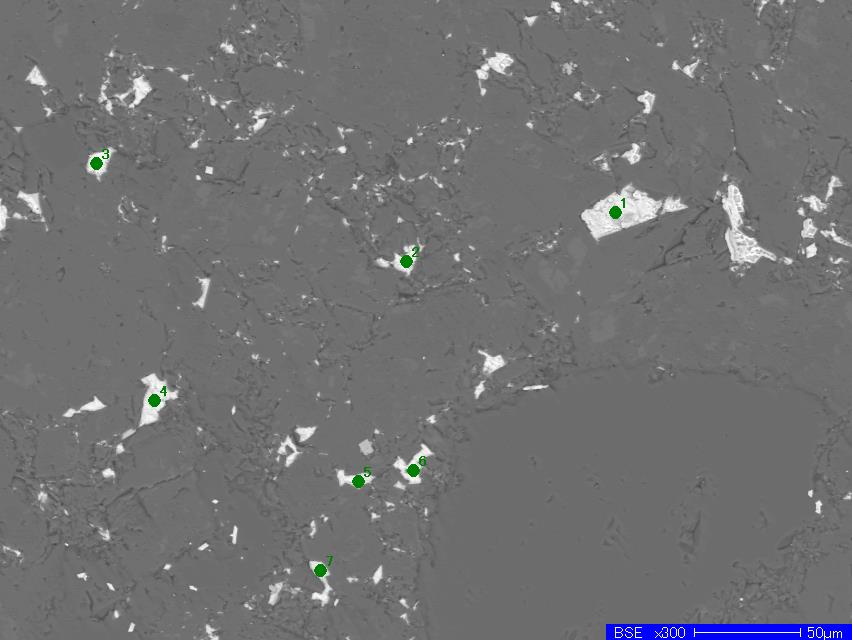

Supplement: Supplementary file 1 [file DataSheet2.zip › Electron Scans/xxy11-24/XH-24-1-1quant.jpeg]

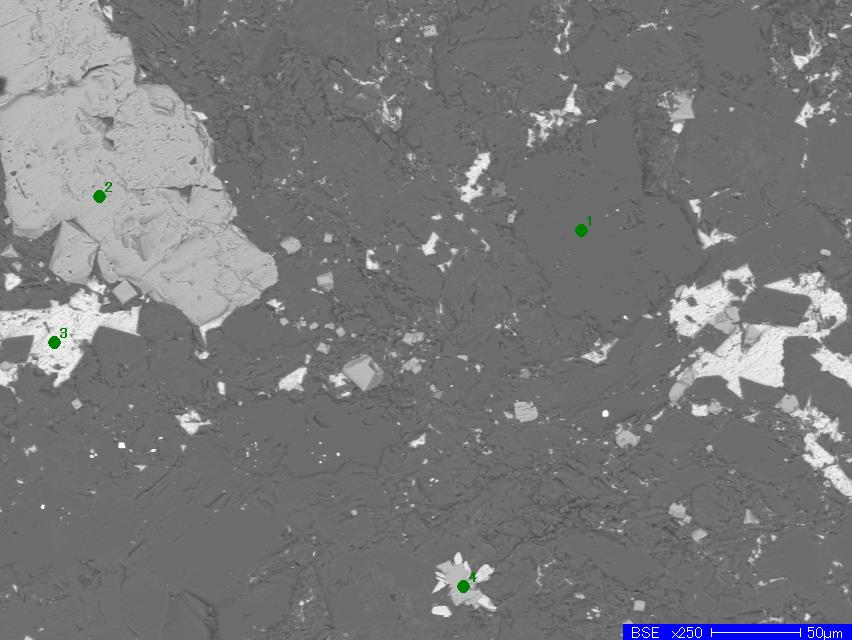

Supplement: Supplementary file 1 [file DataSheet2.zip › Electron Scans/xxy11-24/XH-24-2-1qual.jpeg]

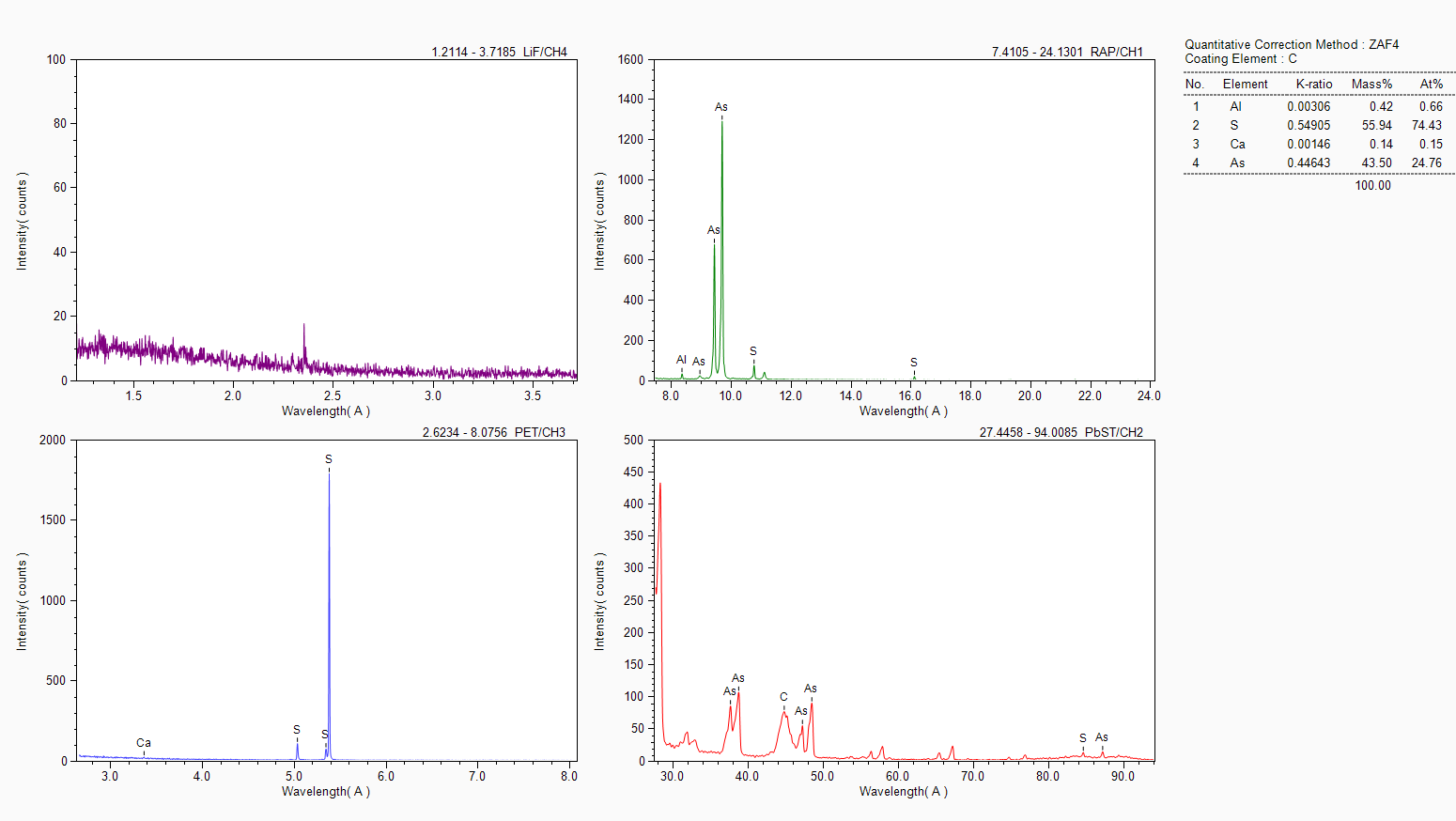

Supplement: Supplementary file 1 [file DataSheet2.zip › Electron Scans/xxy11-24/XH-23qual.bmp]

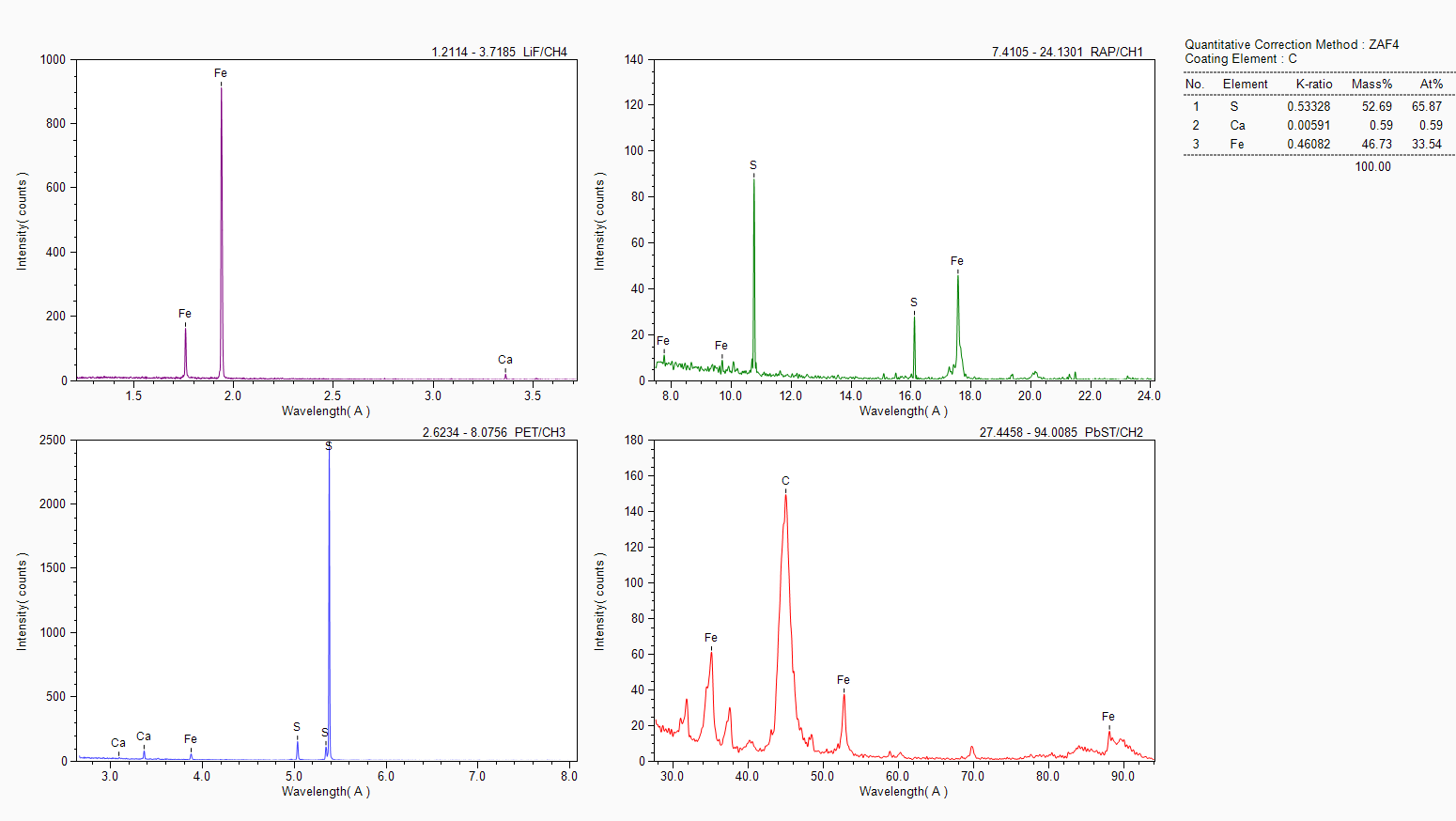

Supplement: Supplementary file 1 [file DataSheet2.zip › Electron Scans/xxy11-24/XH-24-1-1qual-4.bmp]

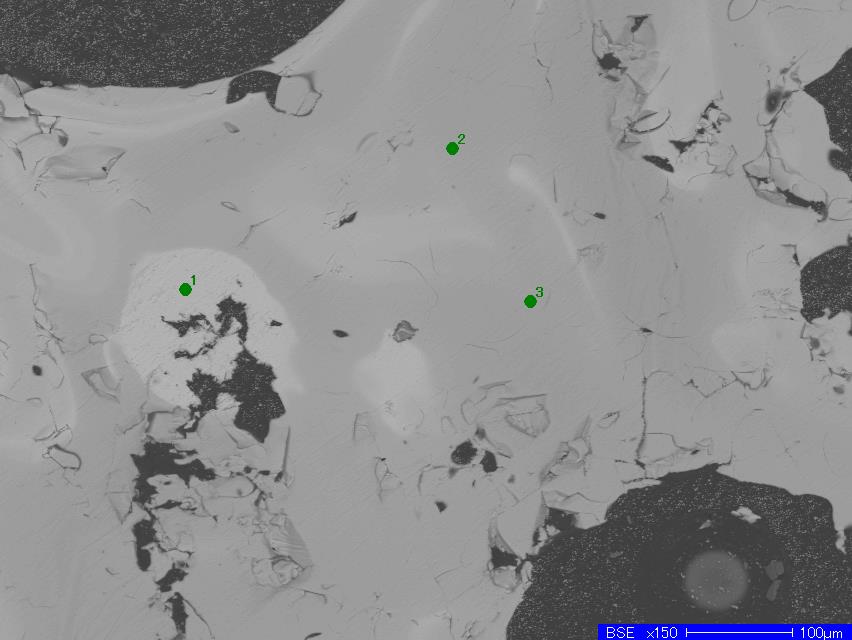

Supplement: Supplementary file 1 [file DataSheet2.zip › Electron Scans/xxy11-24/XH-21-2quant.jpeg]

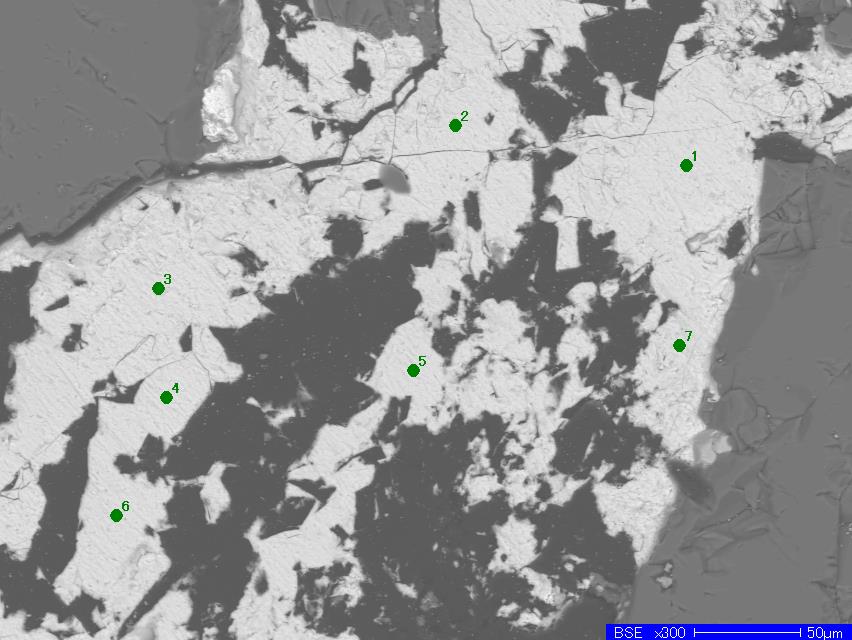

Supplement: Supplementary file 1 [file DataSheet2.zip › Electron Scans/xxy11-24/XH-13-2quant.jpeg]

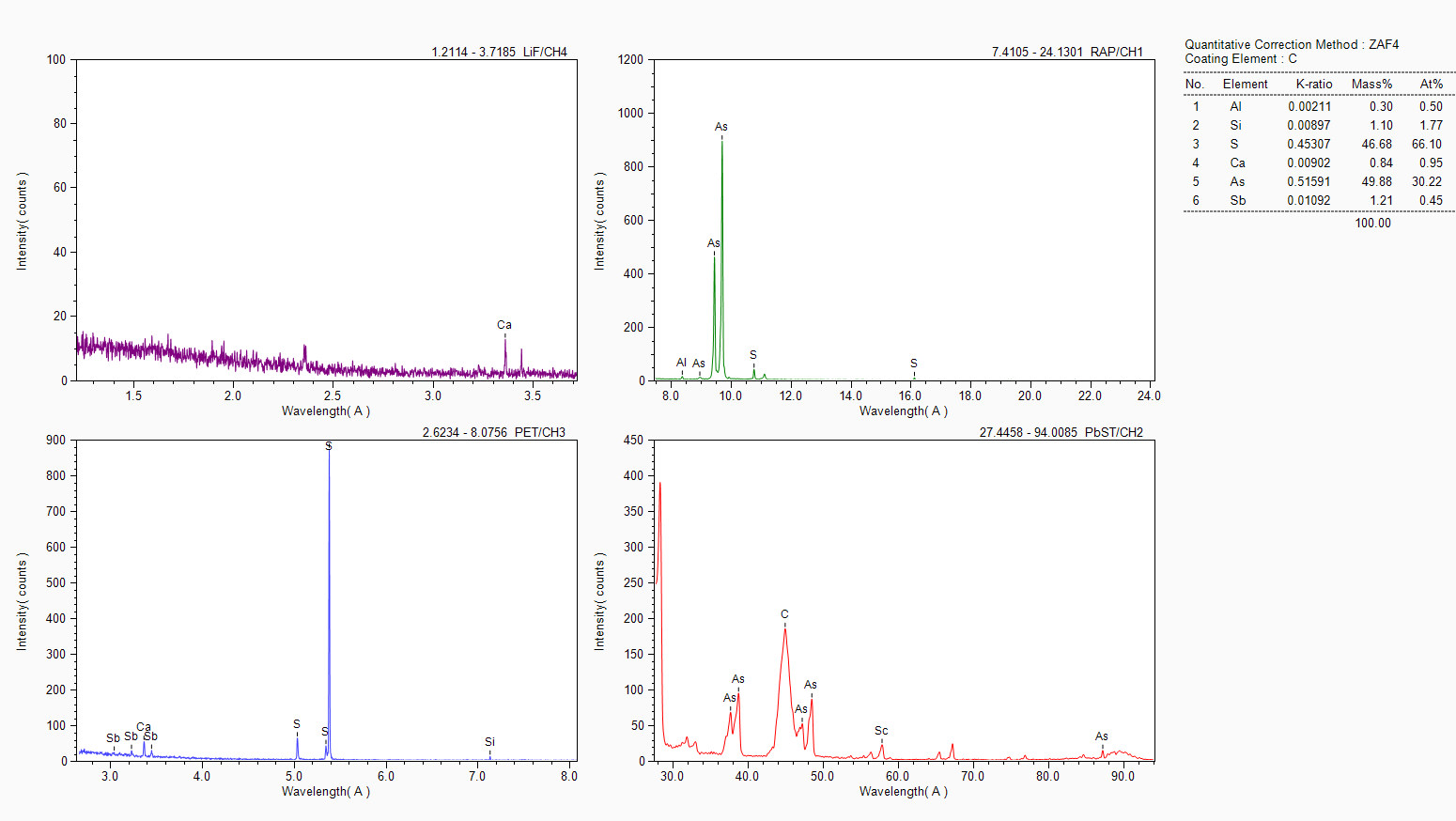

Supplement: Supplementary file 1 [file DataSheet2.zip › Electron Scans/xxy11-24/XH-24-1-1qual-3.bmp]

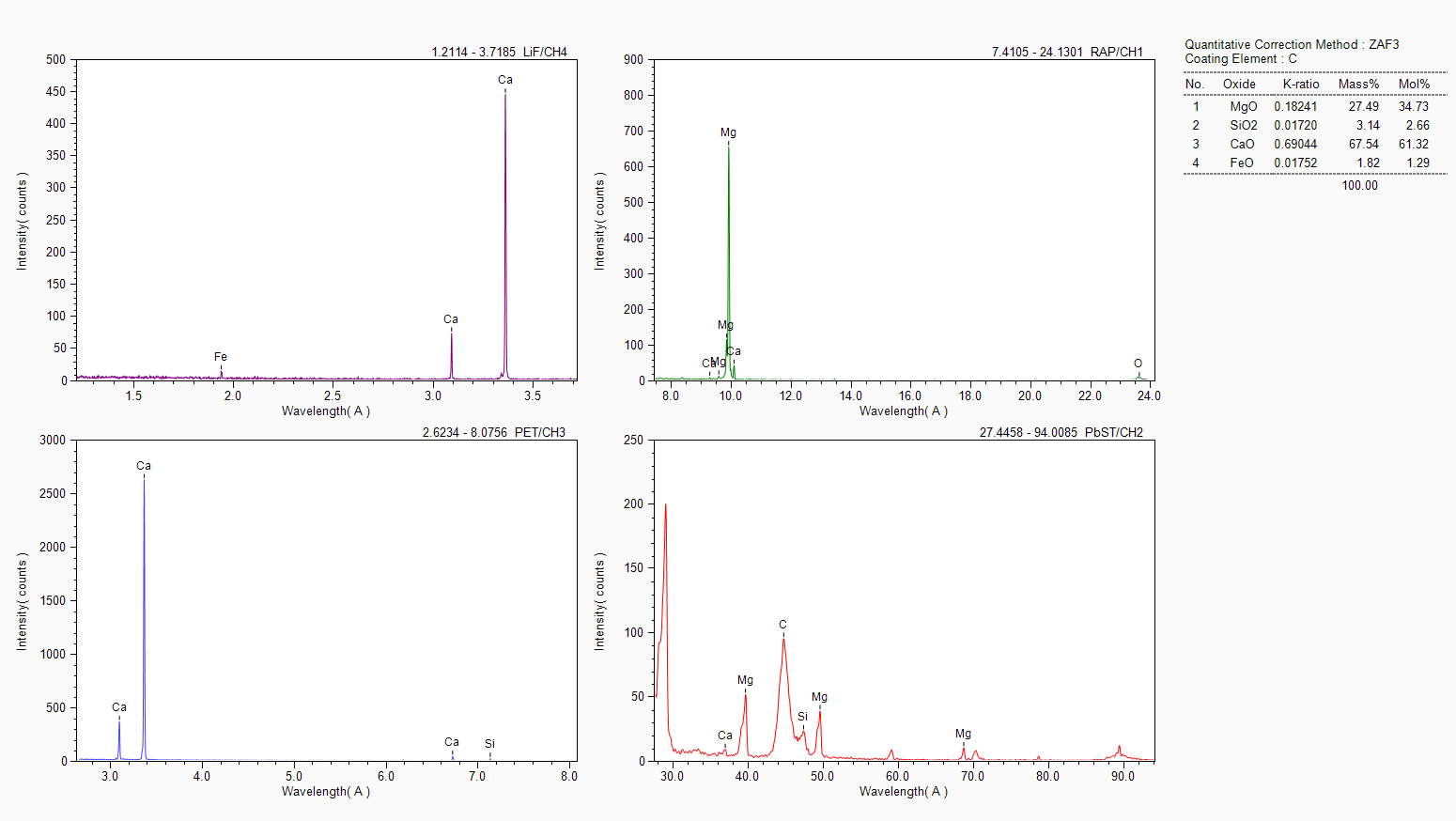

Supplement: Supplementary file 1 [file DataSheet2.zip › Electron Scans/xxy11-24/XH-24-1-1qual-2.bmp]

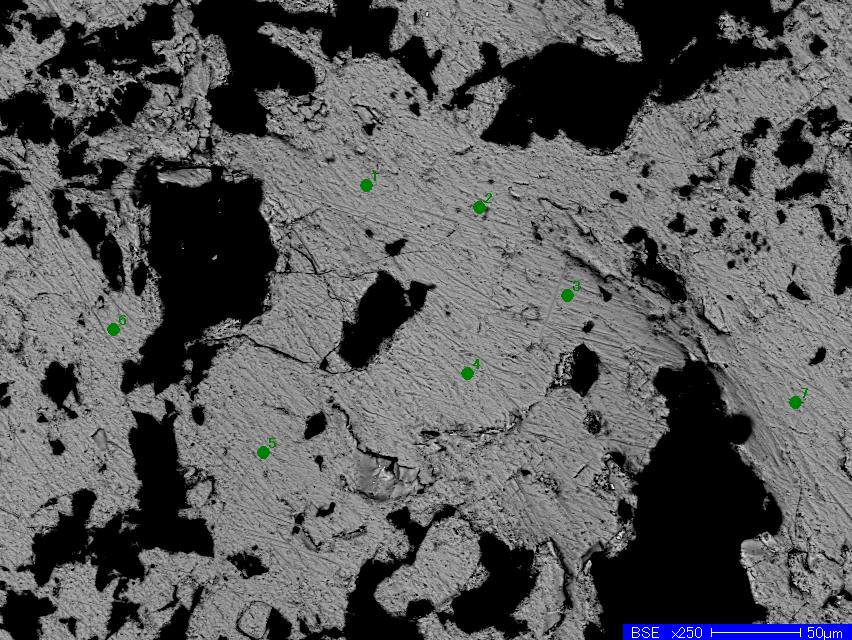

Supplement: Supplementary file 1 [file DataSheet2.zip › Electron Scans/xxy11-24/XH-16quant.jpeg]

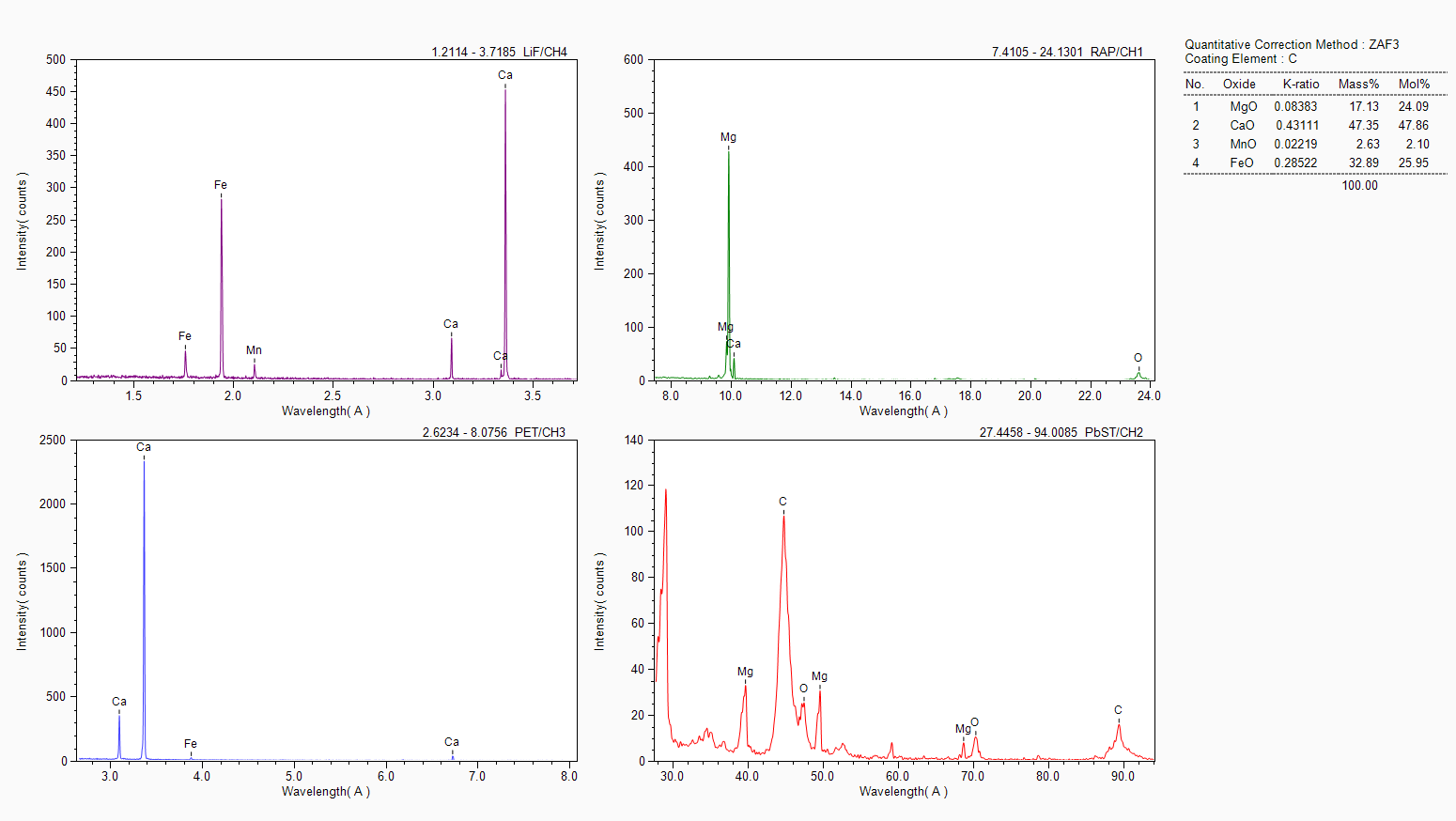

Supplement: Supplementary file 1 [file DataSheet2.zip › Electron Scans/xxy11-24/XH-24-1-1qual-1.bmp]

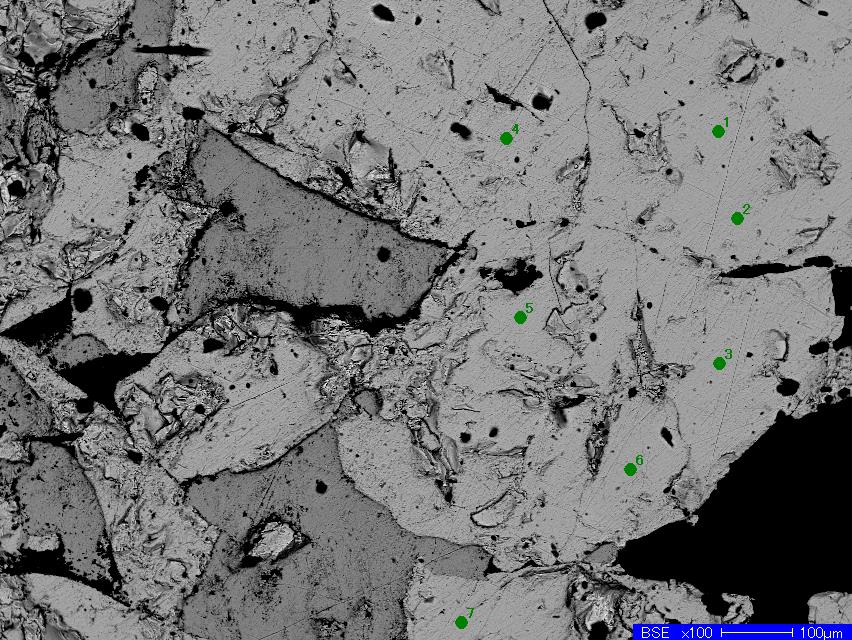

Supplement: Supplementary file 1 [file DataSheet2.zip › Electron Scans/xxy11-24/XH-23quant.jpeg]

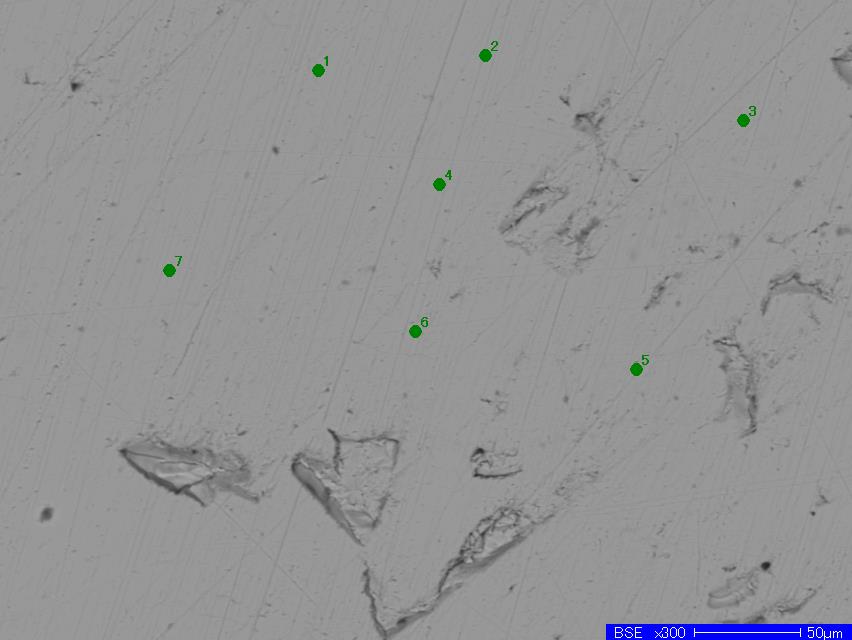

Supplement: Supplementary file 1 [file DataSheet2.zip › Electron Scans/xxy11-24/XH-10-2-1quant.jpeg]

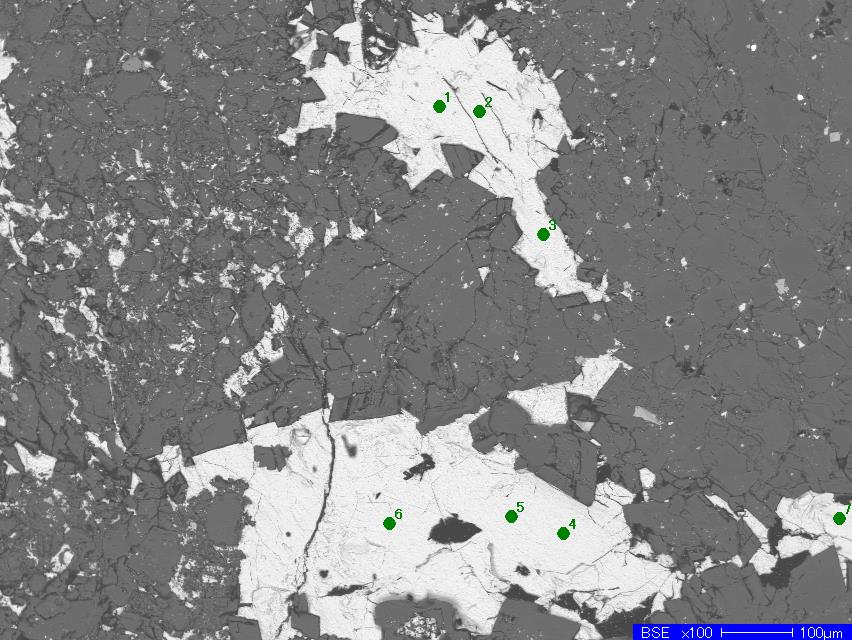

Supplement: Supplementary file 1 [file DataSheet2.zip › Electron Scans/xxy11-24/XH-24-1-2quant.jpeg]

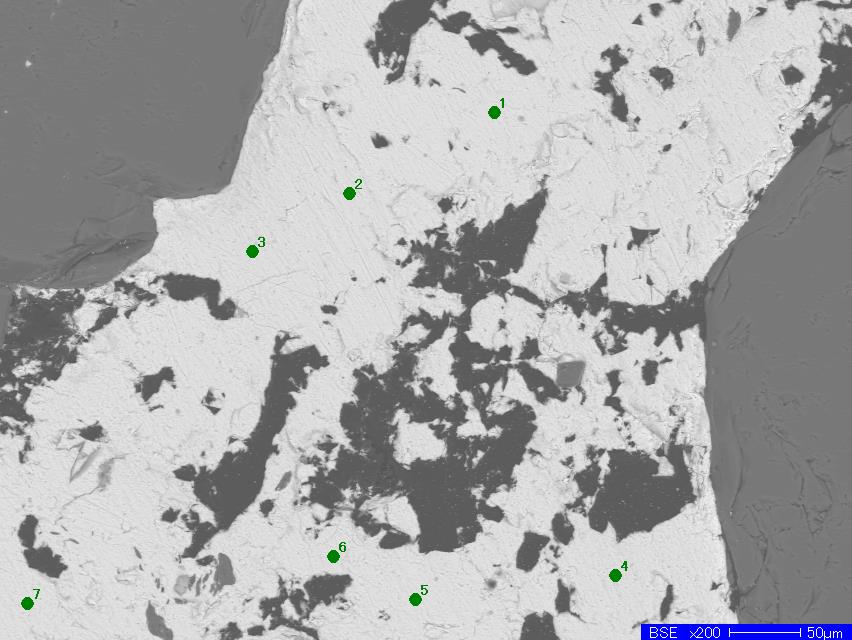

Supplement: Supplementary file 1 [file DataSheet2.zip › Electron Scans/xxy11-24/XH-13-1quant.jpeg]

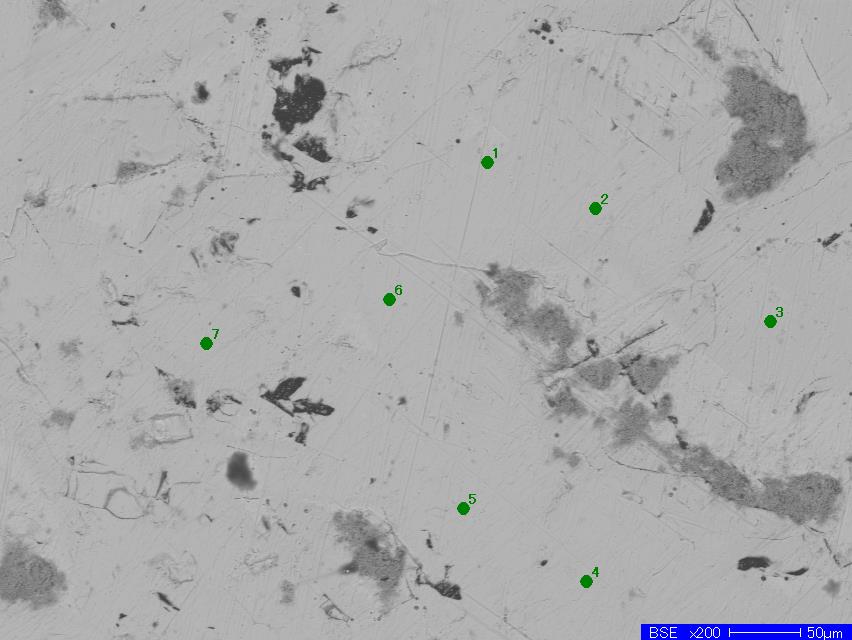

Supplement: Supplementary file 1 [file DataSheet2.zip › Electron Scans/xxy11-24/XH-21-1quant.jpeg]

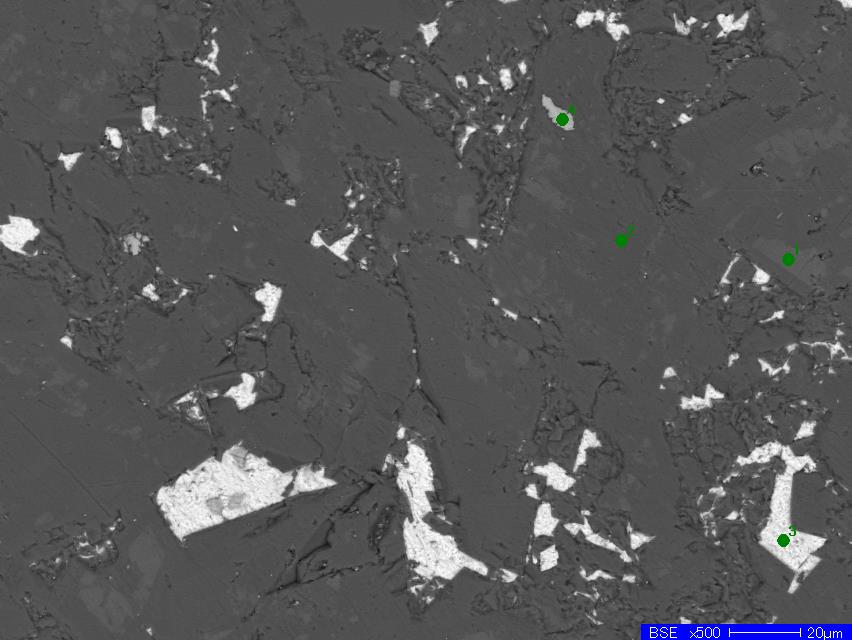

Supplement: Supplementary file 1 [file DataSheet2.zip › Electron Scans/xxy11-24/XH-24-1-1qual.jpeg]

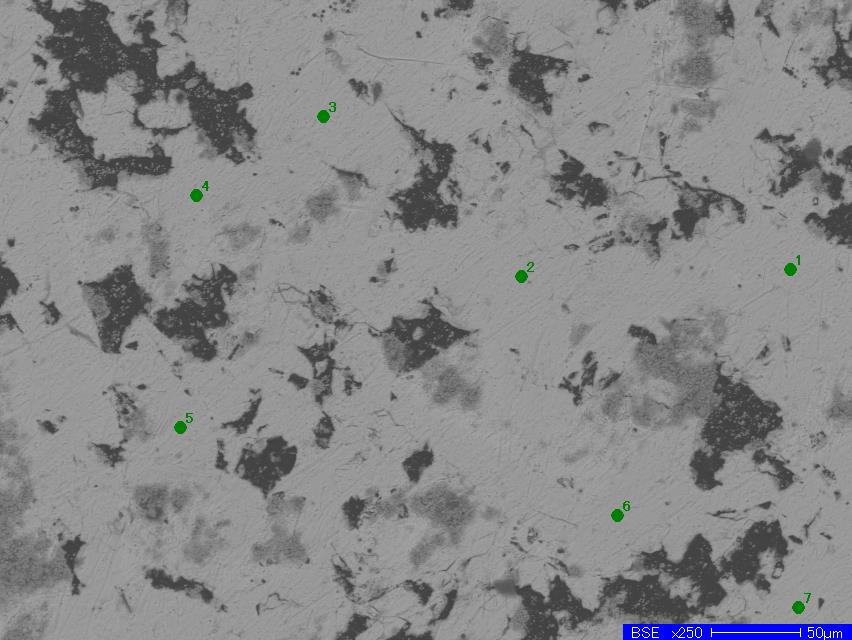

Supplement: Supplementary file 1 [file DataSheet2.zip › Electron Scans/xxy11-24/XH-18quant.jpeg]

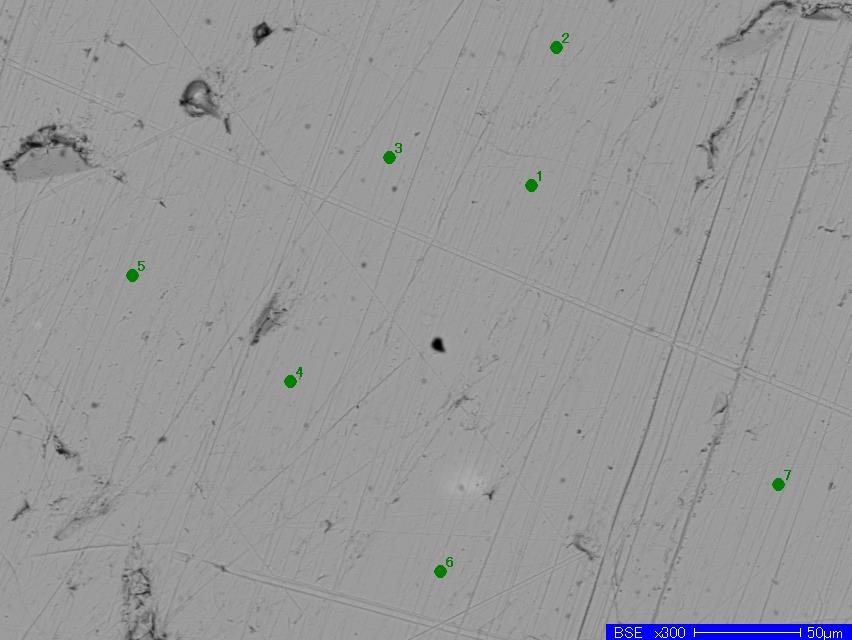

Supplement: Supplementary file 1 [file DataSheet2.zip › Electron Scans/xxy11-24/XH-10-2-2quant.jpeg]

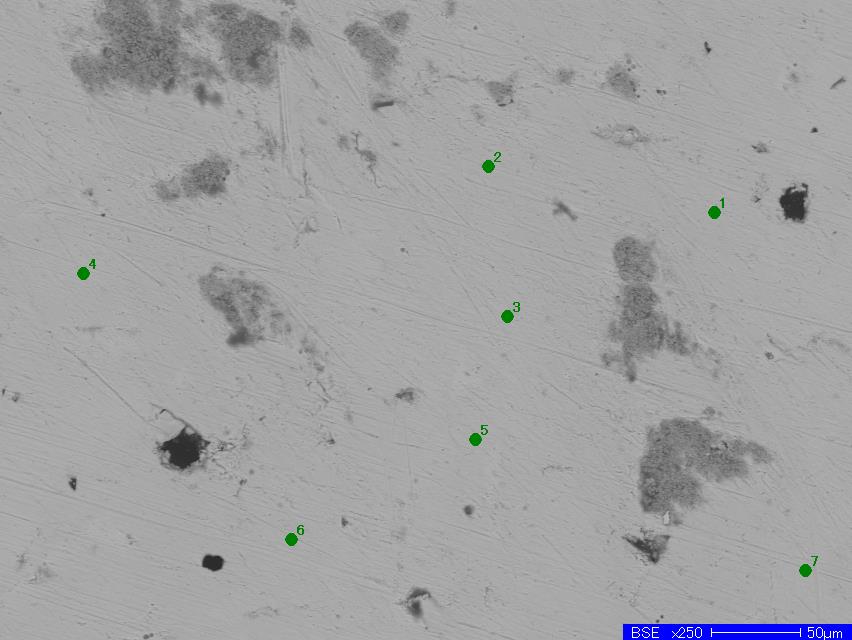

Supplement: Supplementary file 1 [file DataSheet2.zip › Electron Scans/xxy11-24/XH-20quant.jpeg]

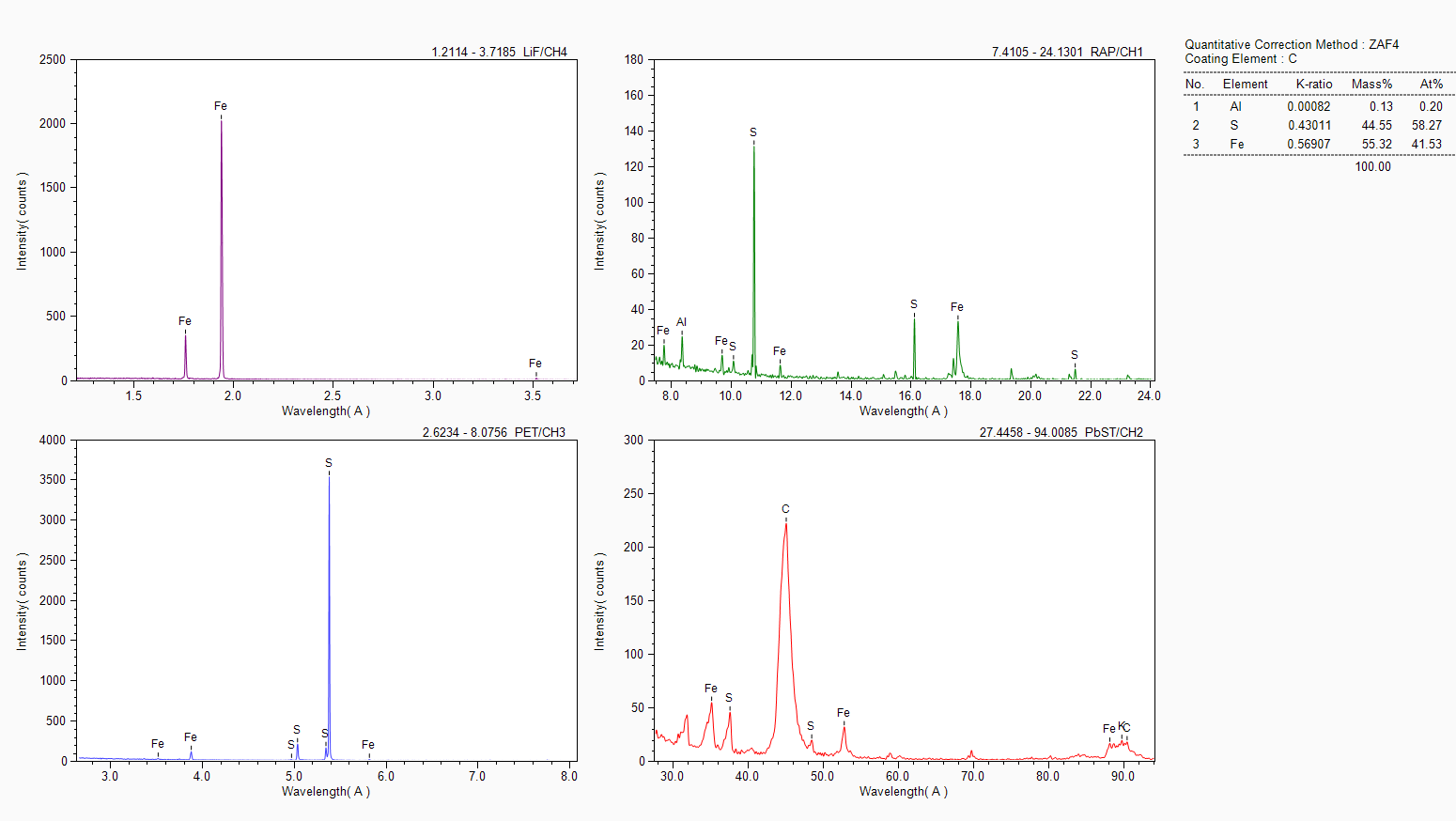

Supplement: Supplementary file 1 [file DataSheet2.zip › Electron Scans/xxy11-24/XH-24-2-2qual-1.bmp]

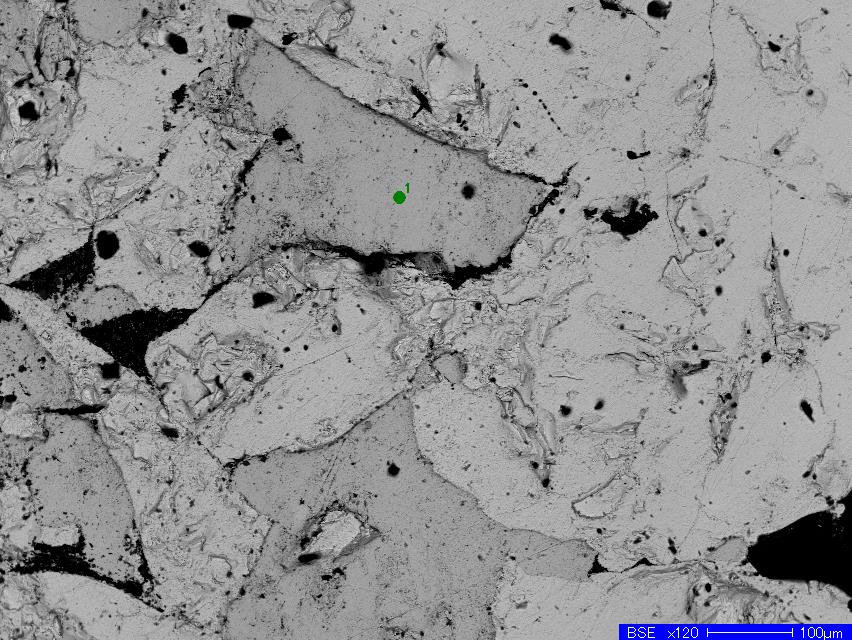

Supplement: Supplementary file 1 [file DataSheet2.zip › Electron Scans/xxy11-24/XH-23qual.jpeg]

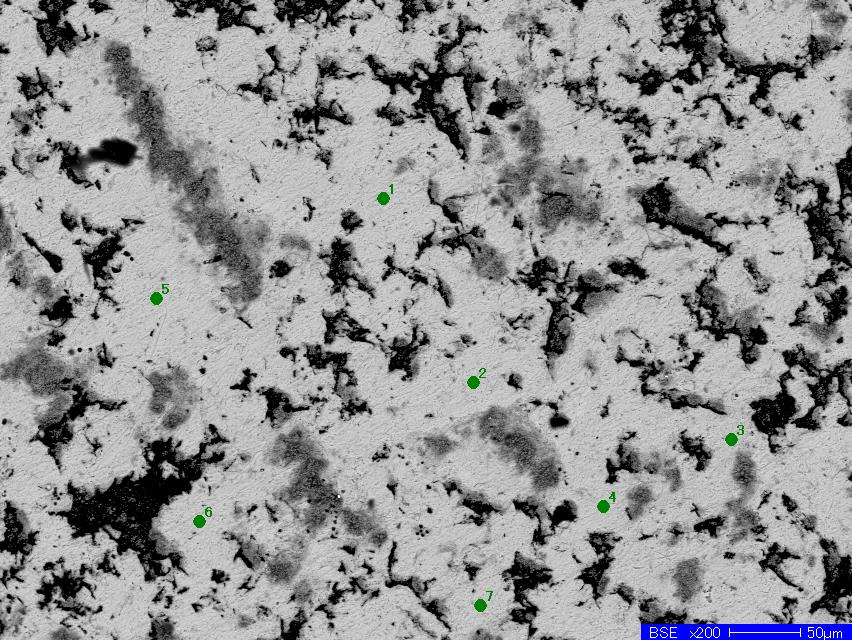

Supplement: Supplementary file 1 [file DataSheet2.zip › Electron Scans/xxy11-24/XH-12quant.jpeg]

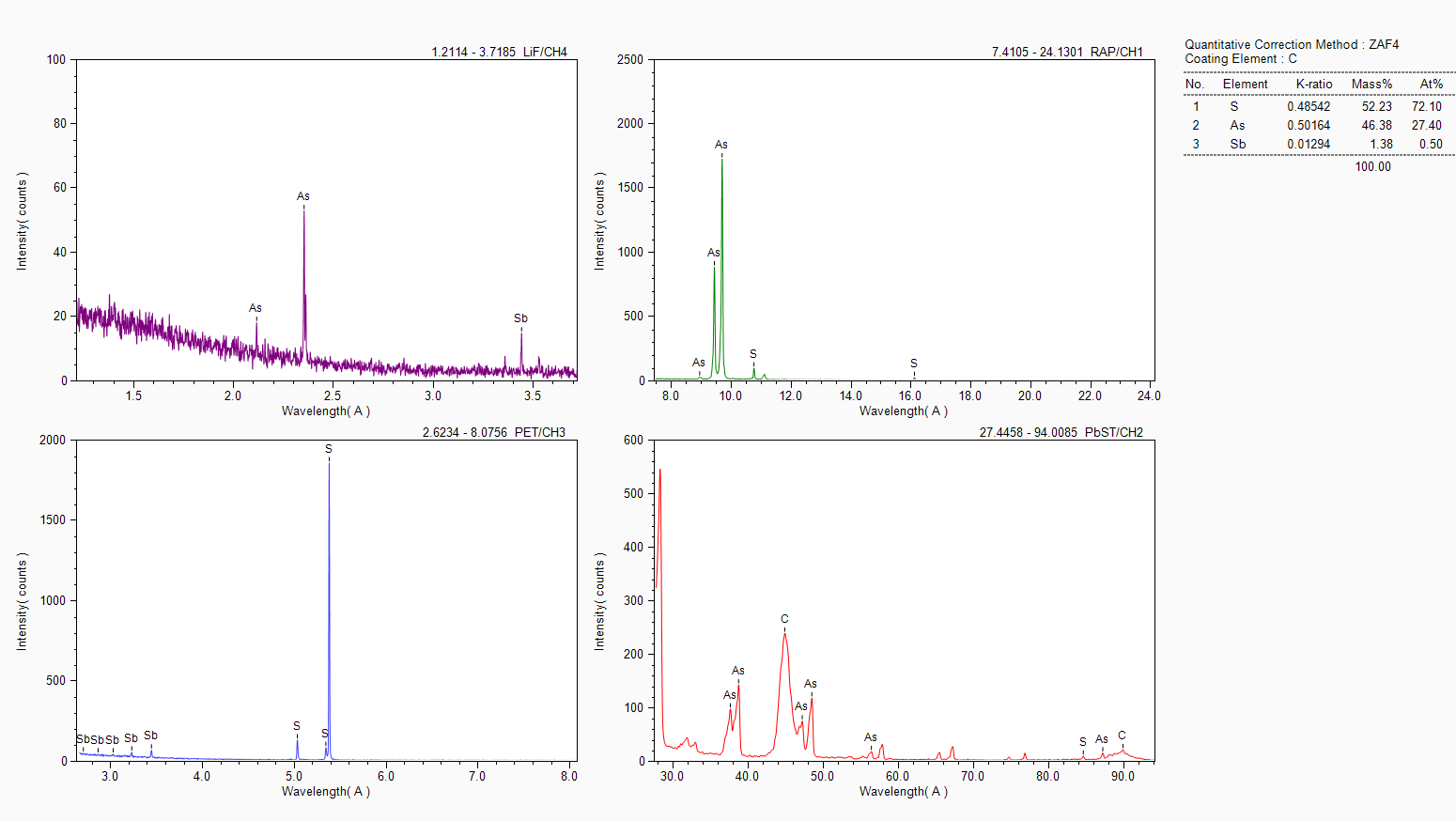

Supplement: Supplementary file 1 [file DataSheet2.zip › Electron Scans/xxy11-24/XH-24-2-2qual-3.bmp]

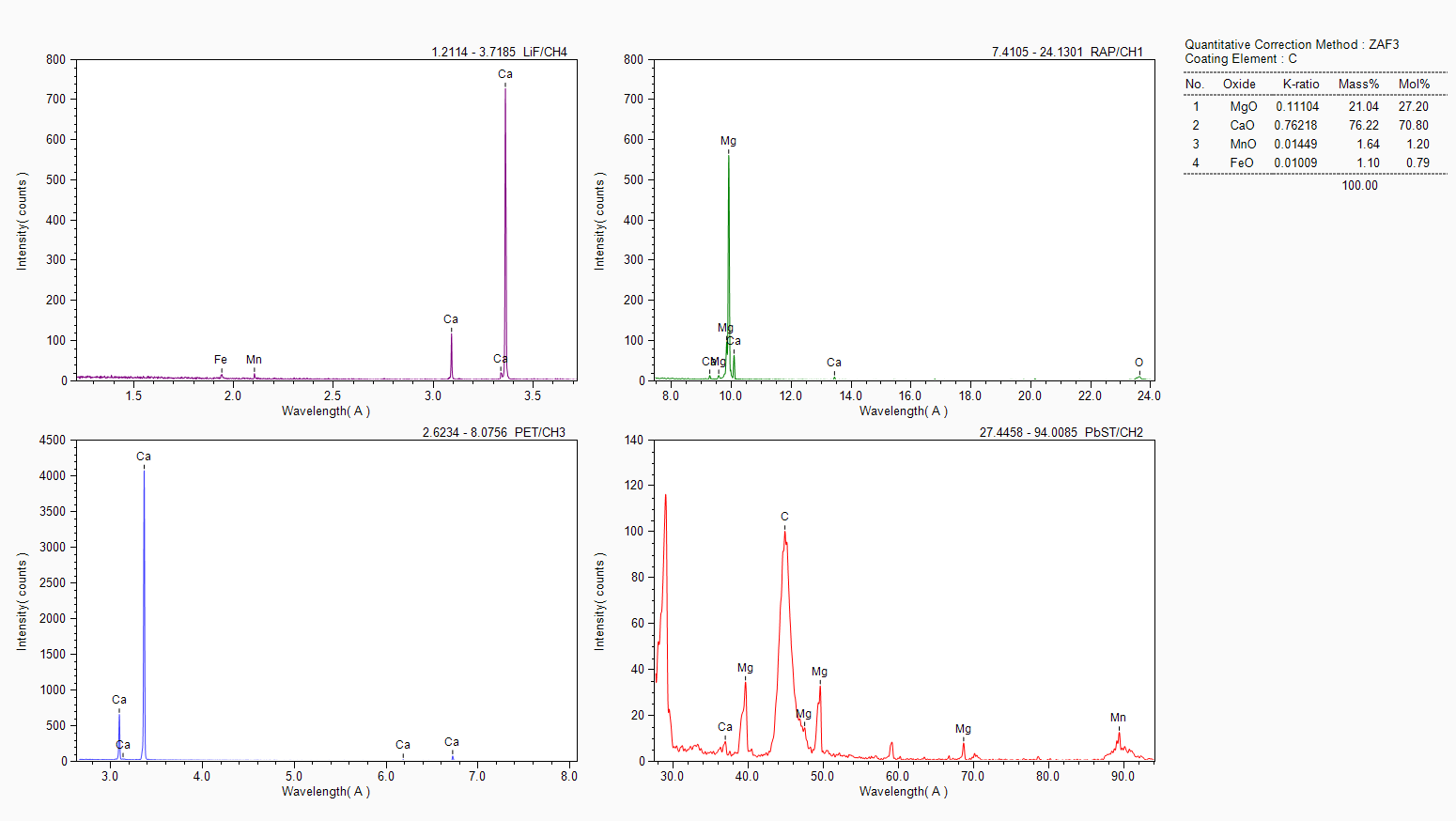

Supplement: Supplementary file 1 [file DataSheet2.zip › Electron Scans/xxy11-24/XH-24-2-2qual-2.bmp]

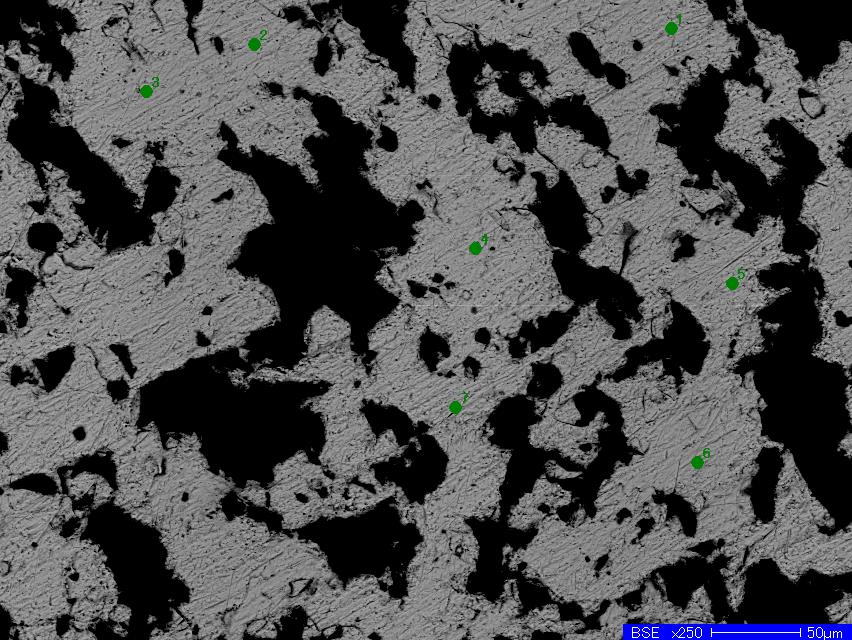

Supplement: Supplementary file 1 [file DataSheet2.zip › Electron Scans/xxy11-24/XH-15-1quant.jpeg]

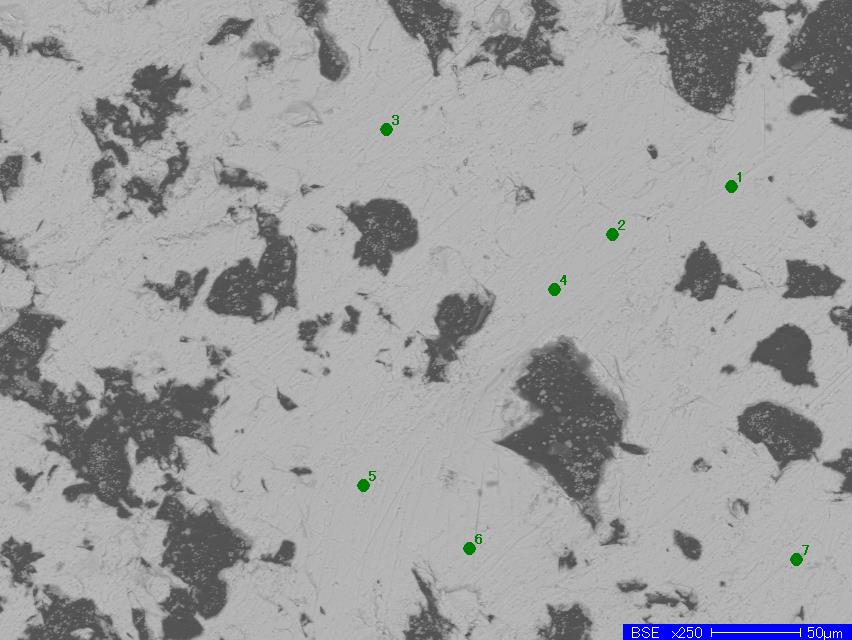

Supplement: Supplementary file 1 [file DataSheet2.zip › Electron Scans/xxy11-24/XH-19quant.jpeg]

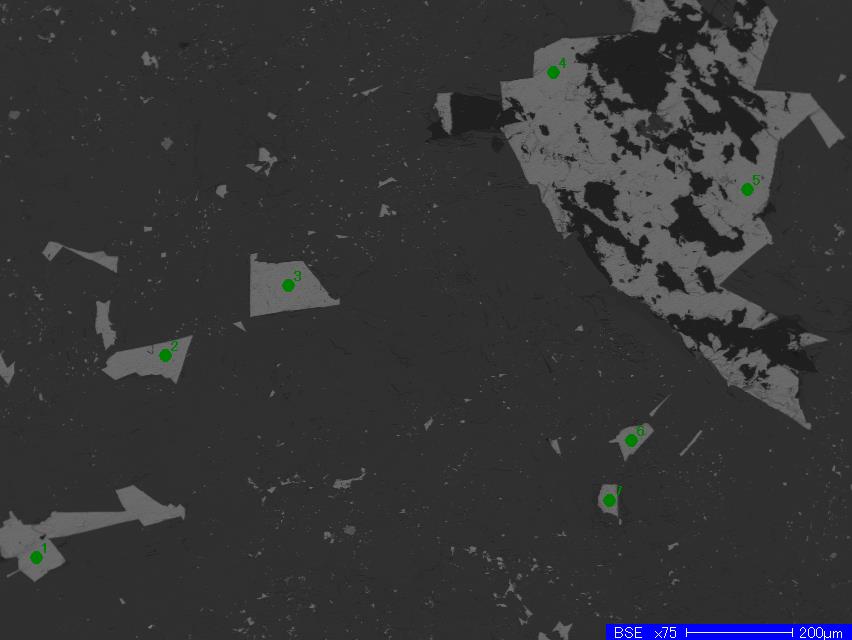

Supplement: Supplementary file 1 [file DataSheet2.zip › Electron Scans/xxy11-24/XH-24-2-2quant.jpeg]

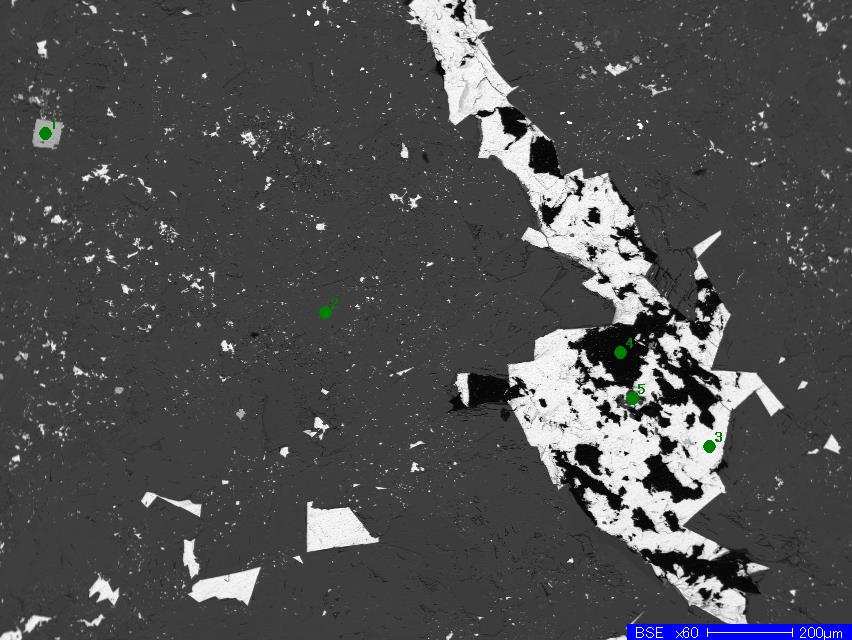

Supplement: Supplementary file 1 [file DataSheet2.zip › Electron Scans/xxy11-24/XH-24-2-2qual.jpeg]

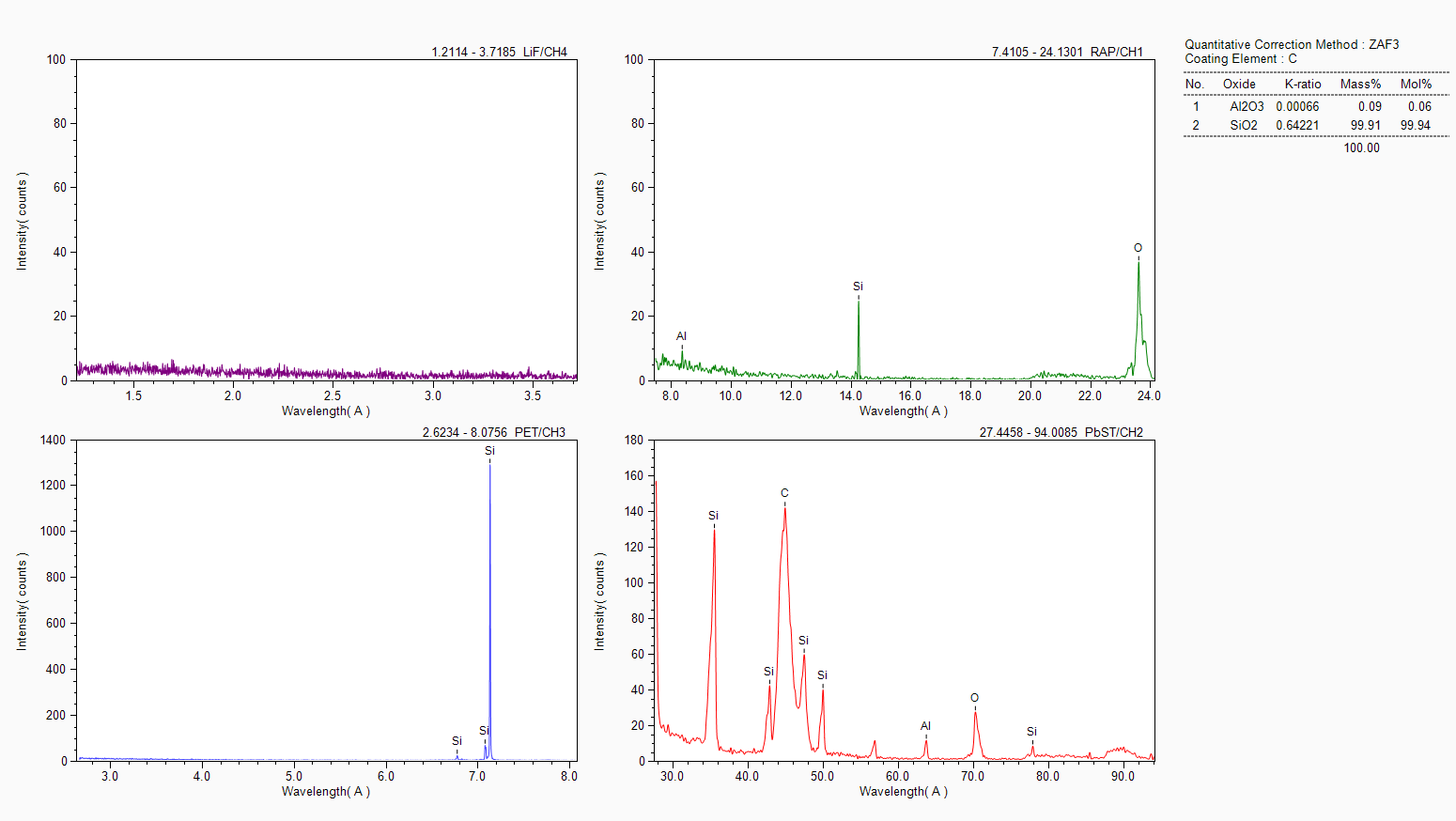

Supplement: Supplementary file 1 [file DataSheet2.zip › Electron Scans/xxy11-24/XH-13qual.bmp]

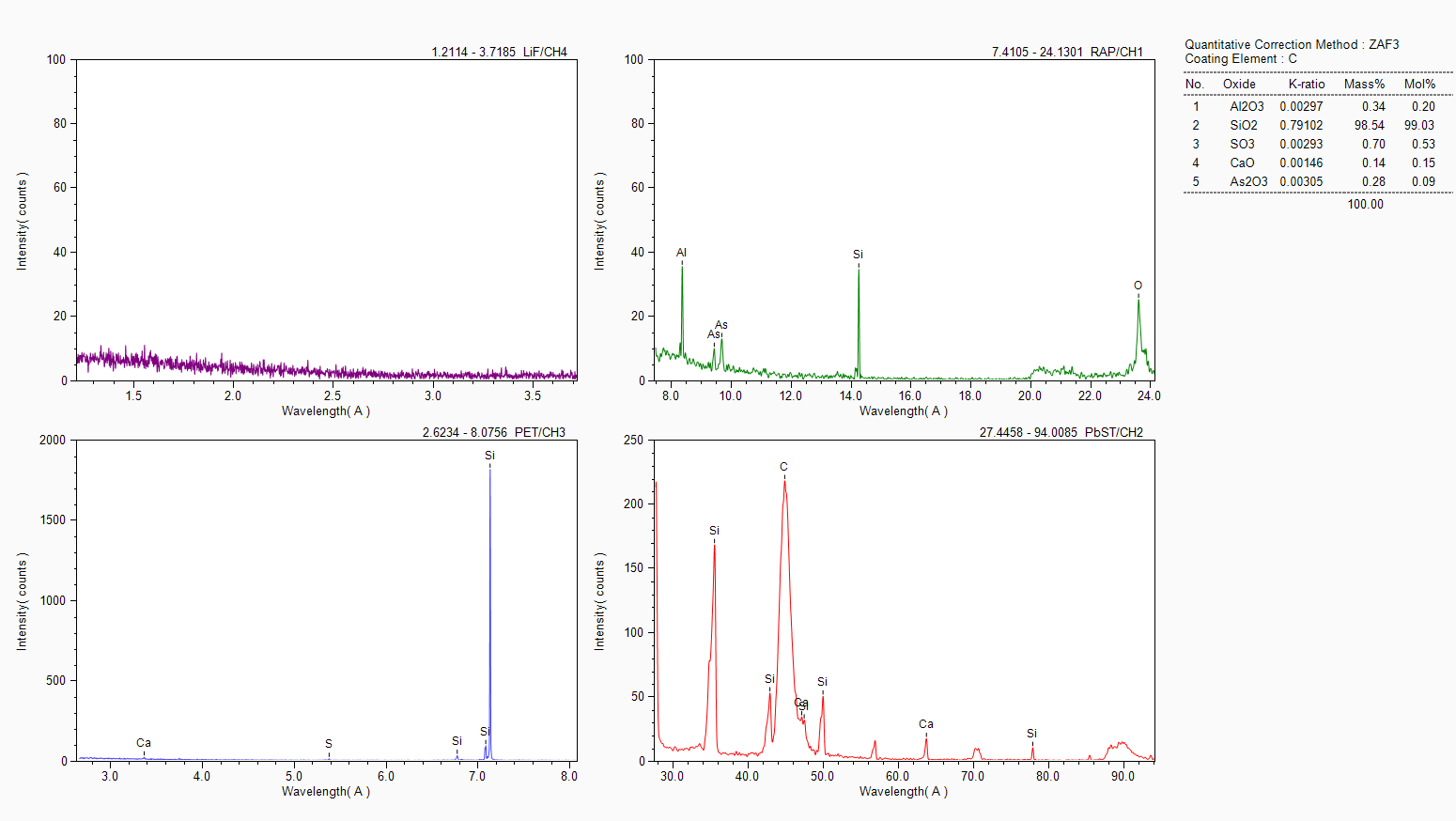

Supplement: Supplementary file 1 [file DataSheet2.zip › Electron Scans/xxy11-24/XH-24-2-2qual-5.bmp]

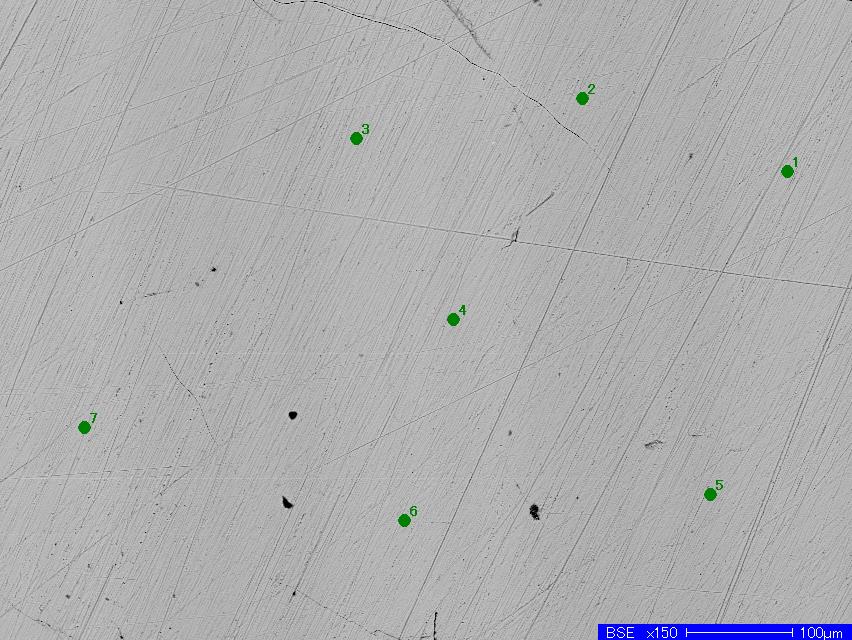

Supplement: Supplementary file 1 [file DataSheet2.zip › Electron Scans/xxy11-24/XH-14quant.jpeg]

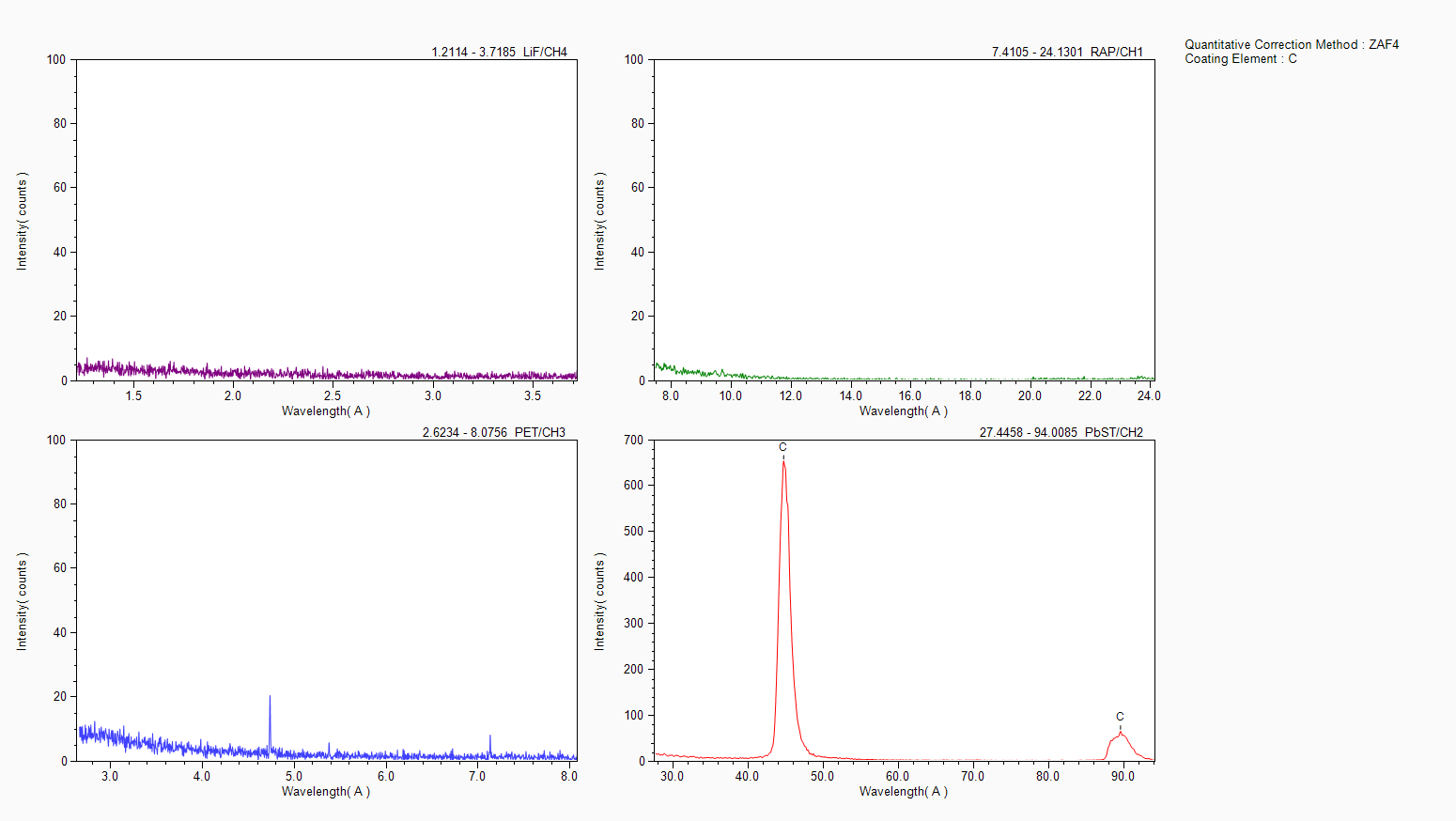

Supplement: Supplementary file 1 [file DataSheet2.zip › Electron Scans/xxy11-24/XH-24-2-2qual-4.bmp]

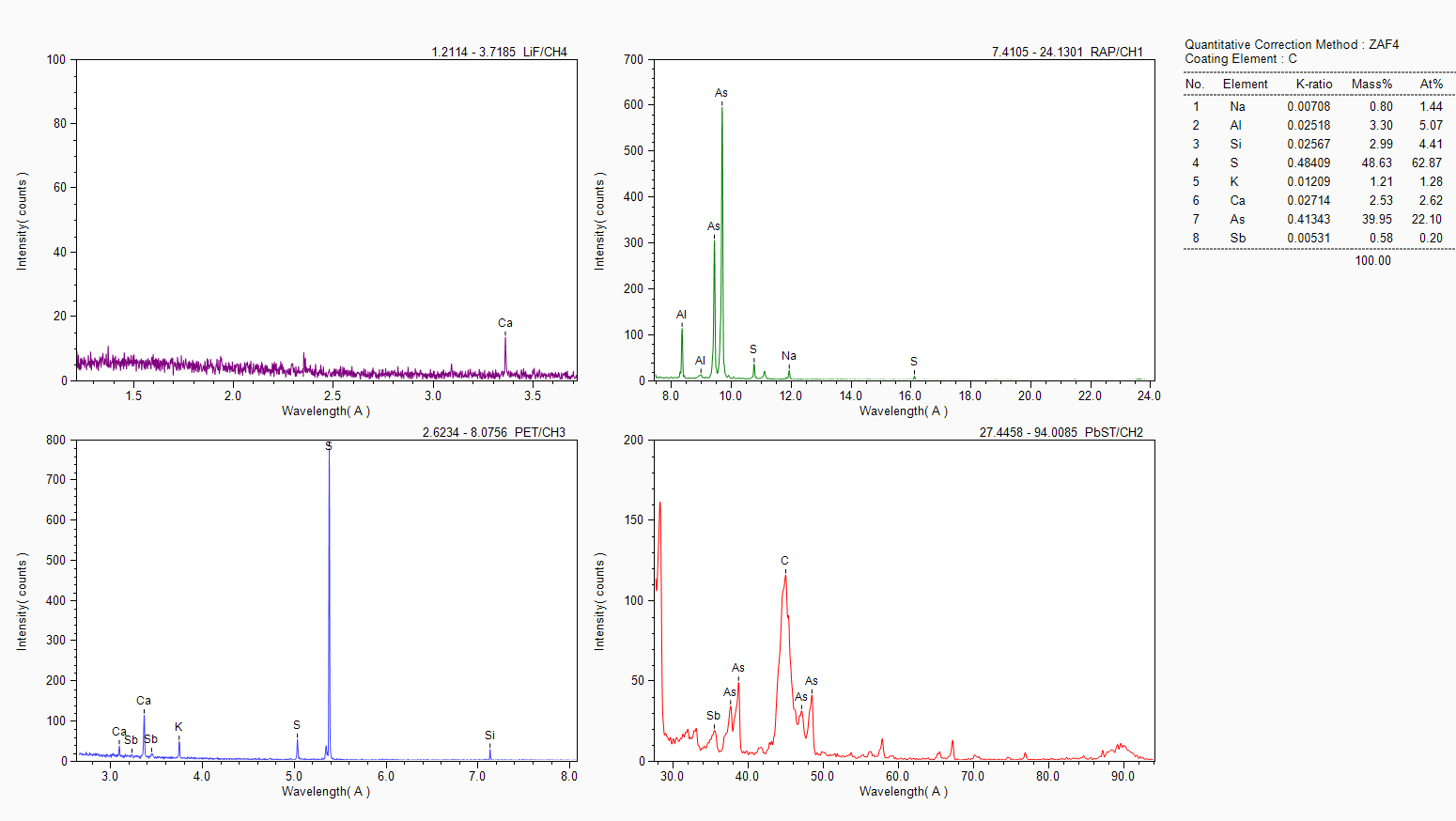

Supplement: Supplementary file 1 [file DataSheet2.zip › Electron Scans/xxy1-10/XH-4qual-3.bmp]

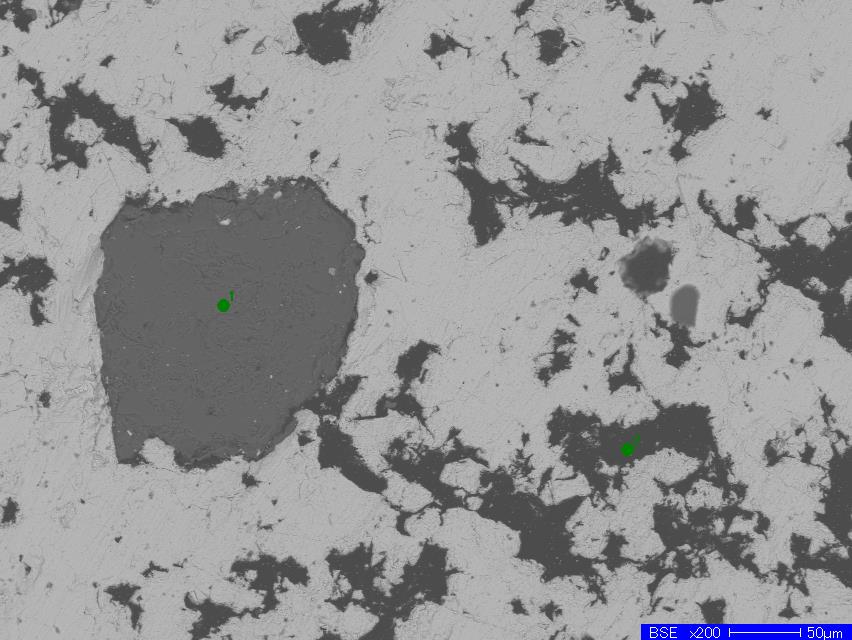

Supplement: Supplementary file 1 [file DataSheet2.zip › Electron Scans/xxy1-10/XH-8qual.jpeg]

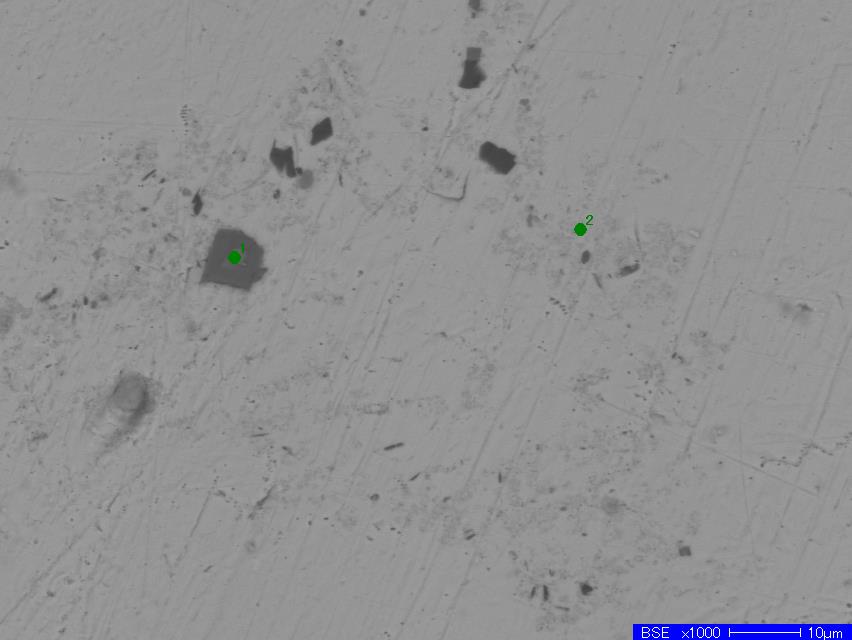

Supplement: Supplementary file 1 [file DataSheet2.zip › Electron Scans/xxy1-10/XH-9qual.jpeg]

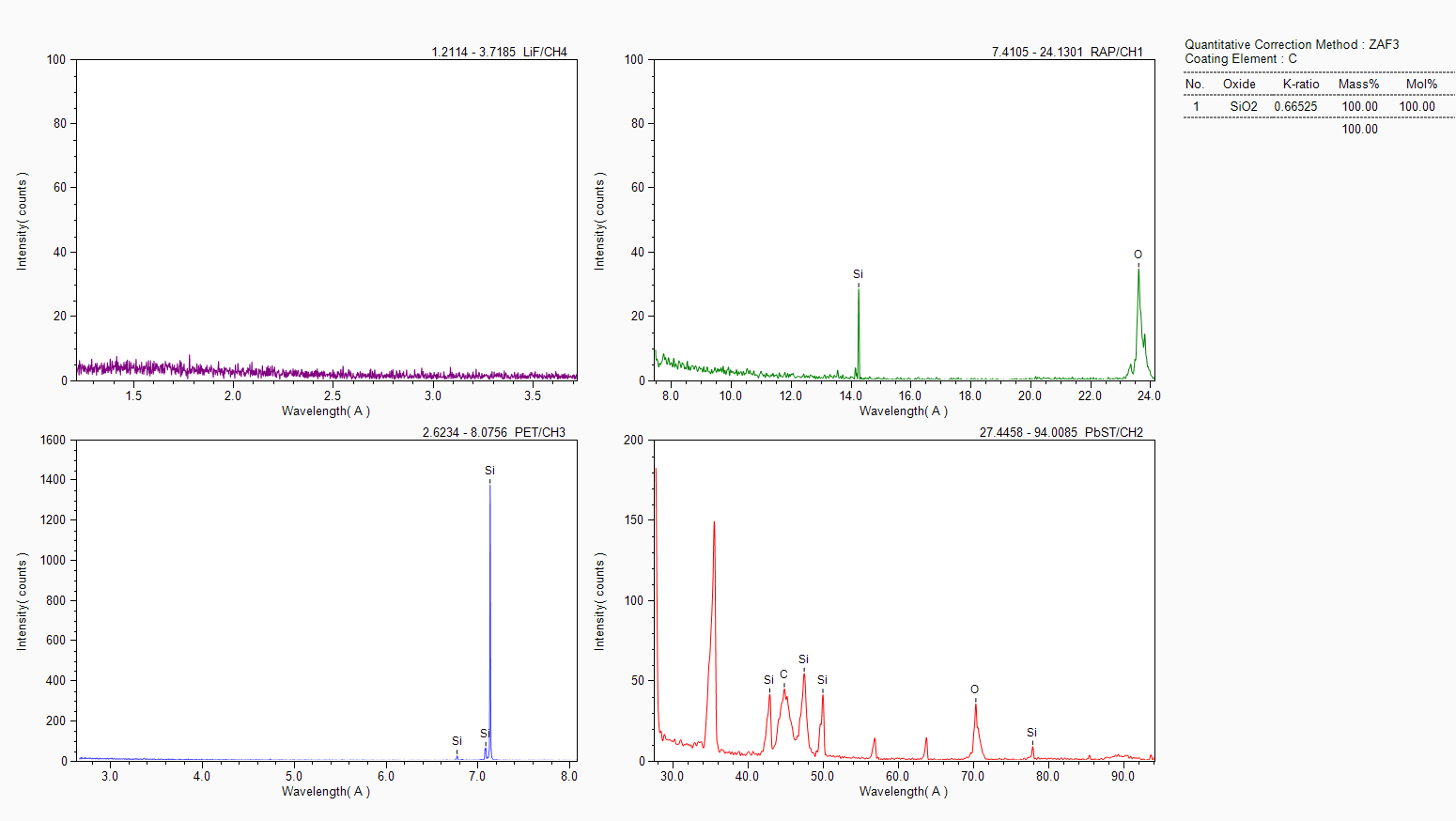

Supplement: Supplementary file 1 [file DataSheet2.zip › Electron Scans/xxy1-10/XH-4qual-2.bmp]

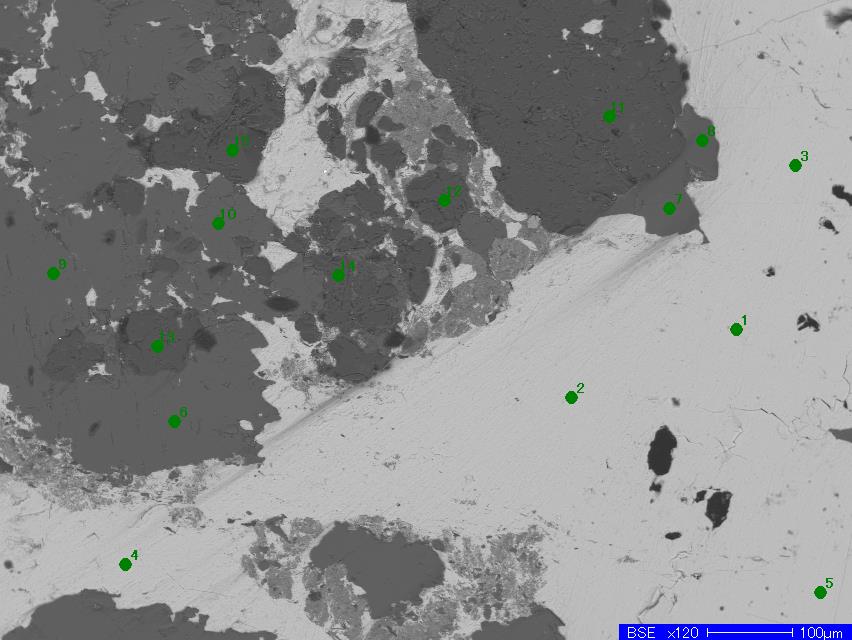

Supplement: Supplementary file 1 [file DataSheet2.zip › Electron Scans/xxy1-10/XH-4-1quant.jpeg]

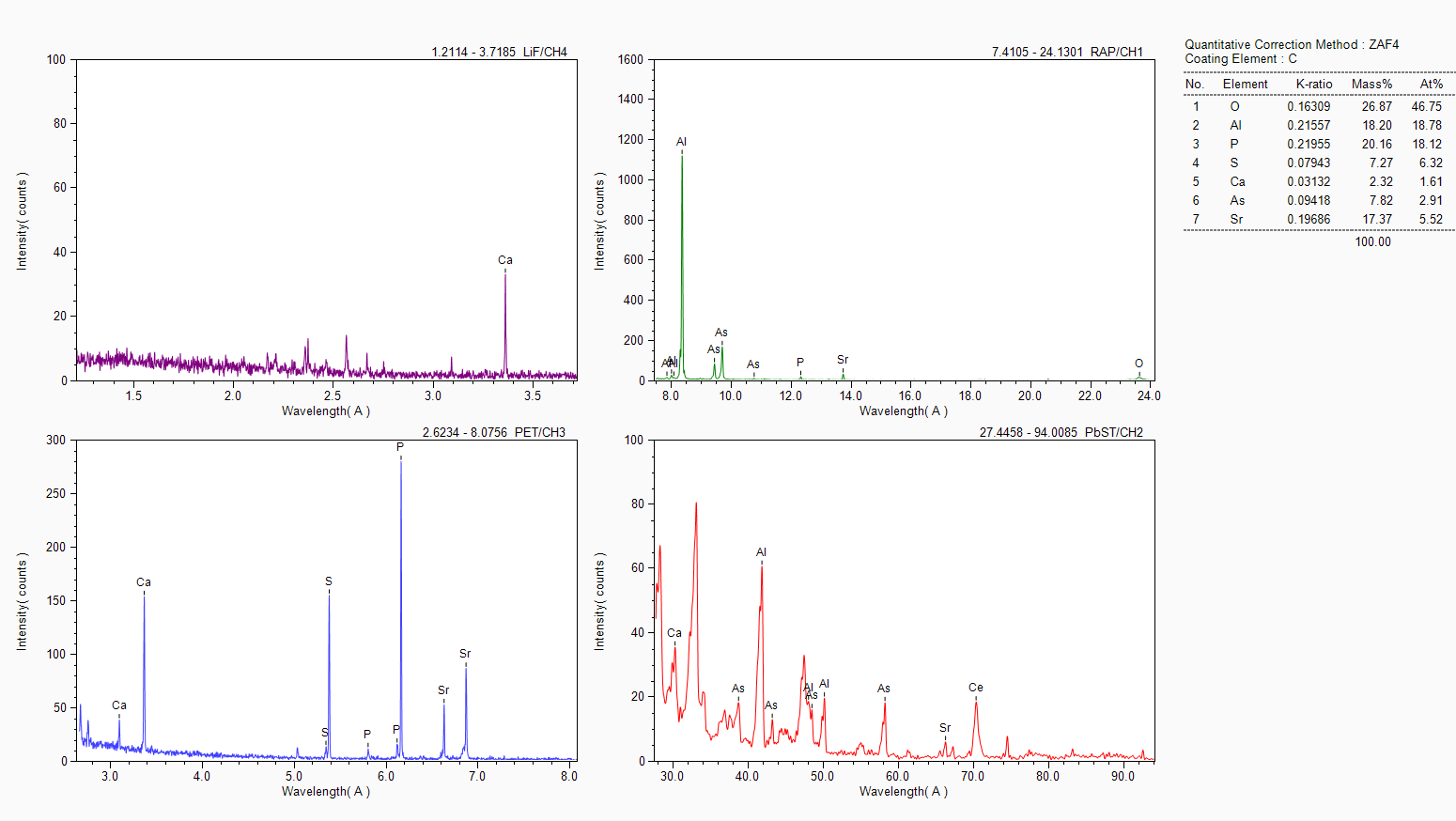

Supplement: Supplementary file 1 [file DataSheet2.zip › Electron Scans/xxy1-10/XH-9qual-1.bmp]

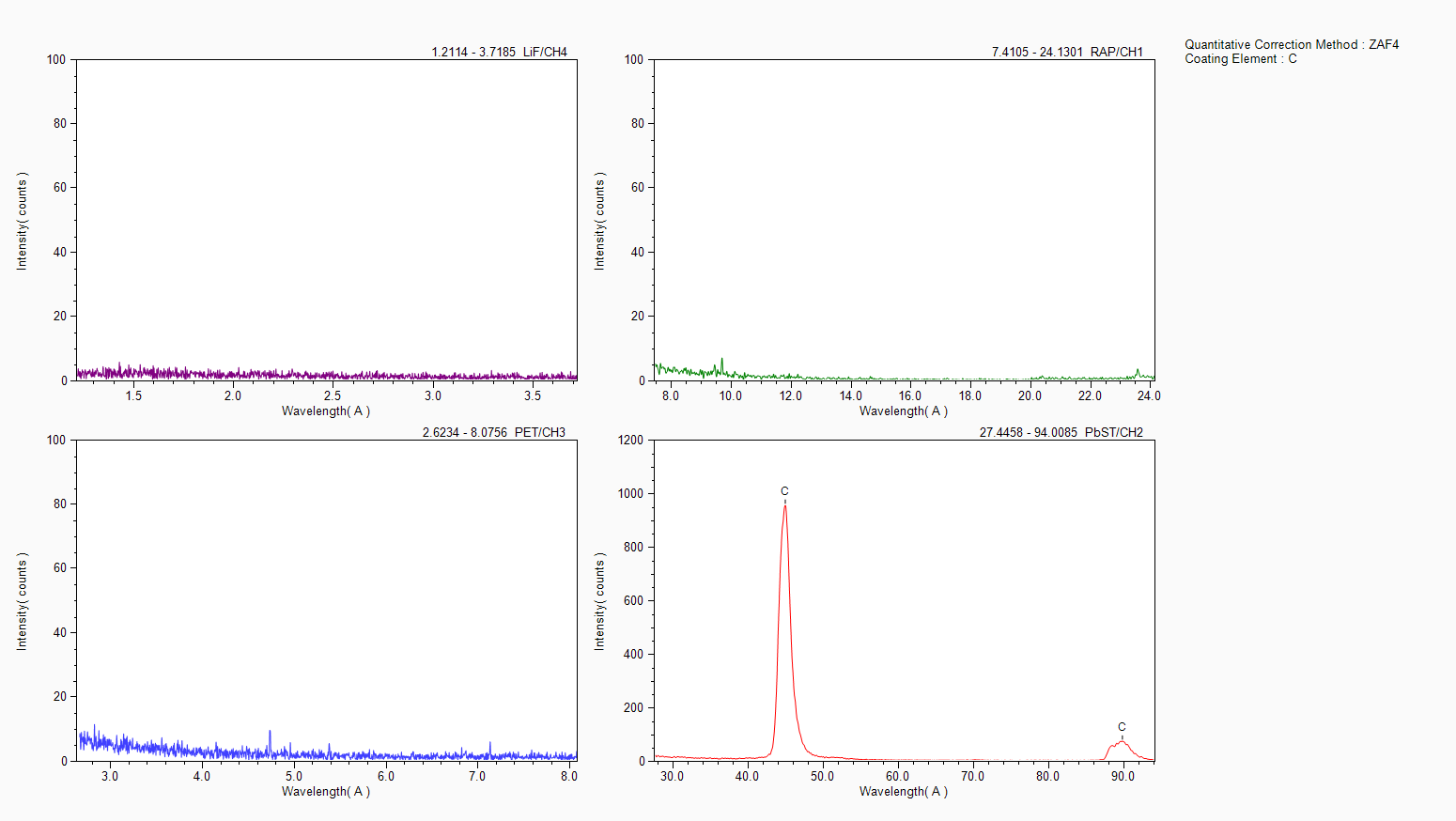

Supplement: Supplementary file 1 [file DataSheet2.zip › Electron Scans/xxy1-10/XH-7qual.bmp]

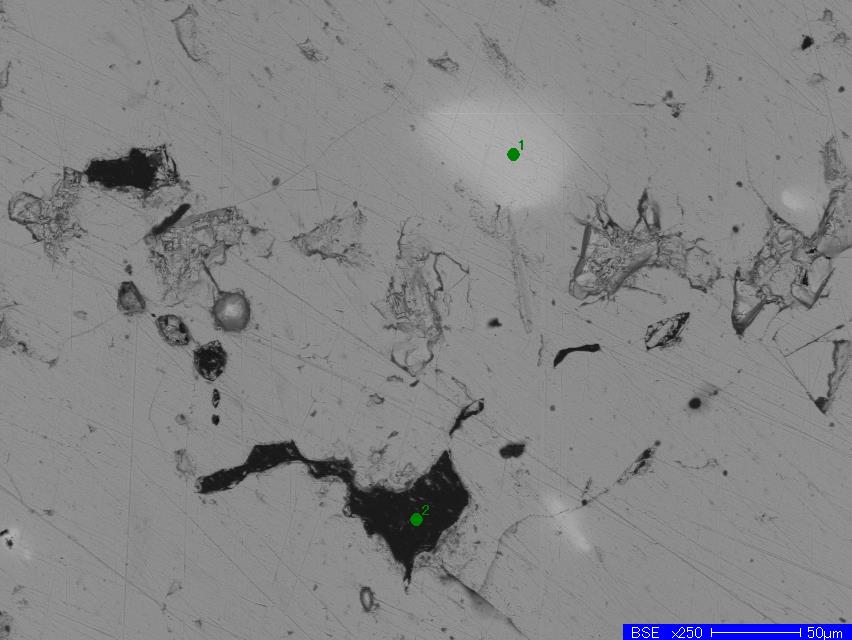

Supplement: Supplementary file 1 [file DataSheet2.zip › Electron Scans/xxy1-10/XH-10-1qual.jpeg]

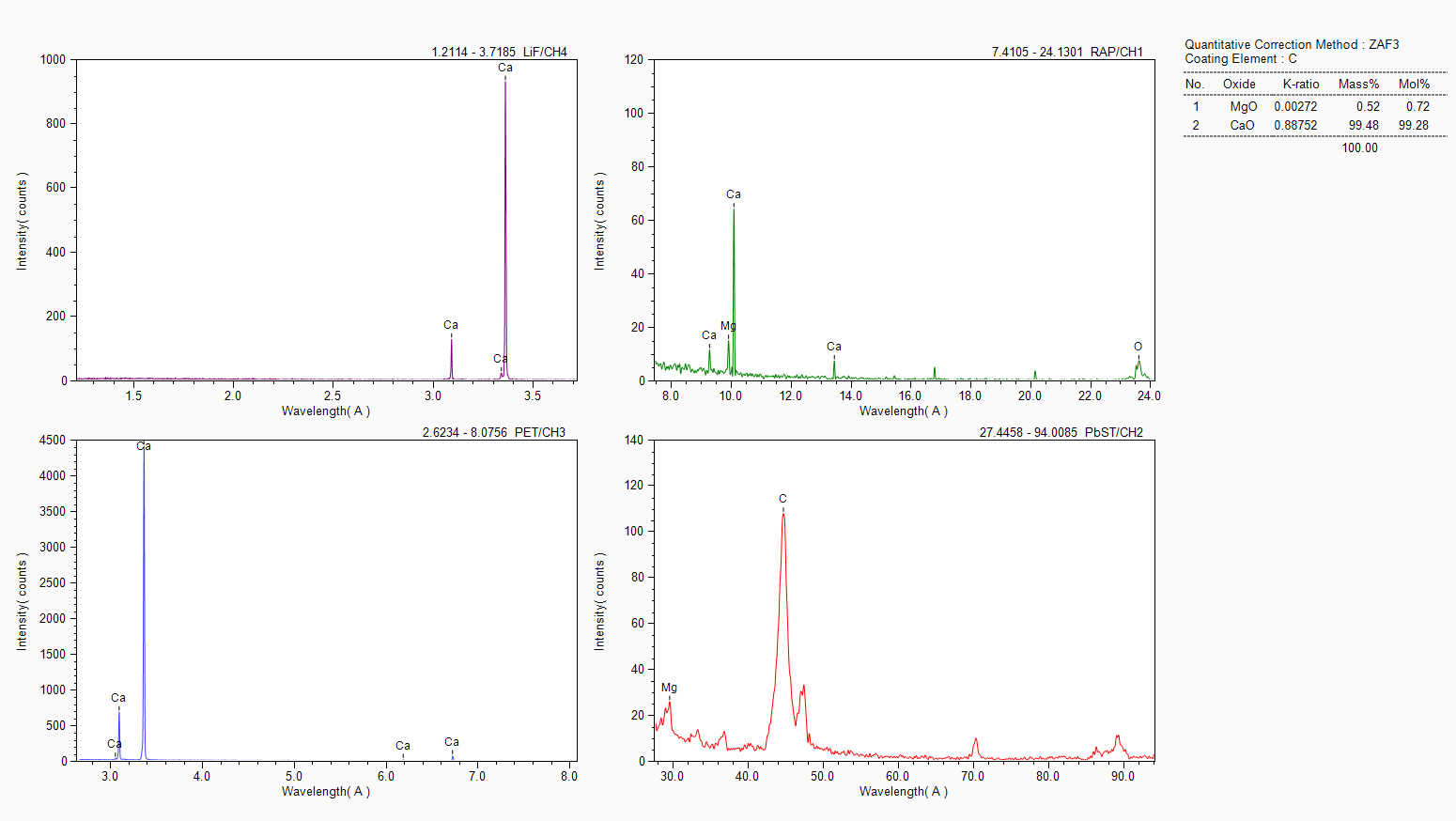

Supplement: Supplementary file 1 [file DataSheet2.zip › Electron Scans/xxy1-10/XH-4qual-1.bmp]

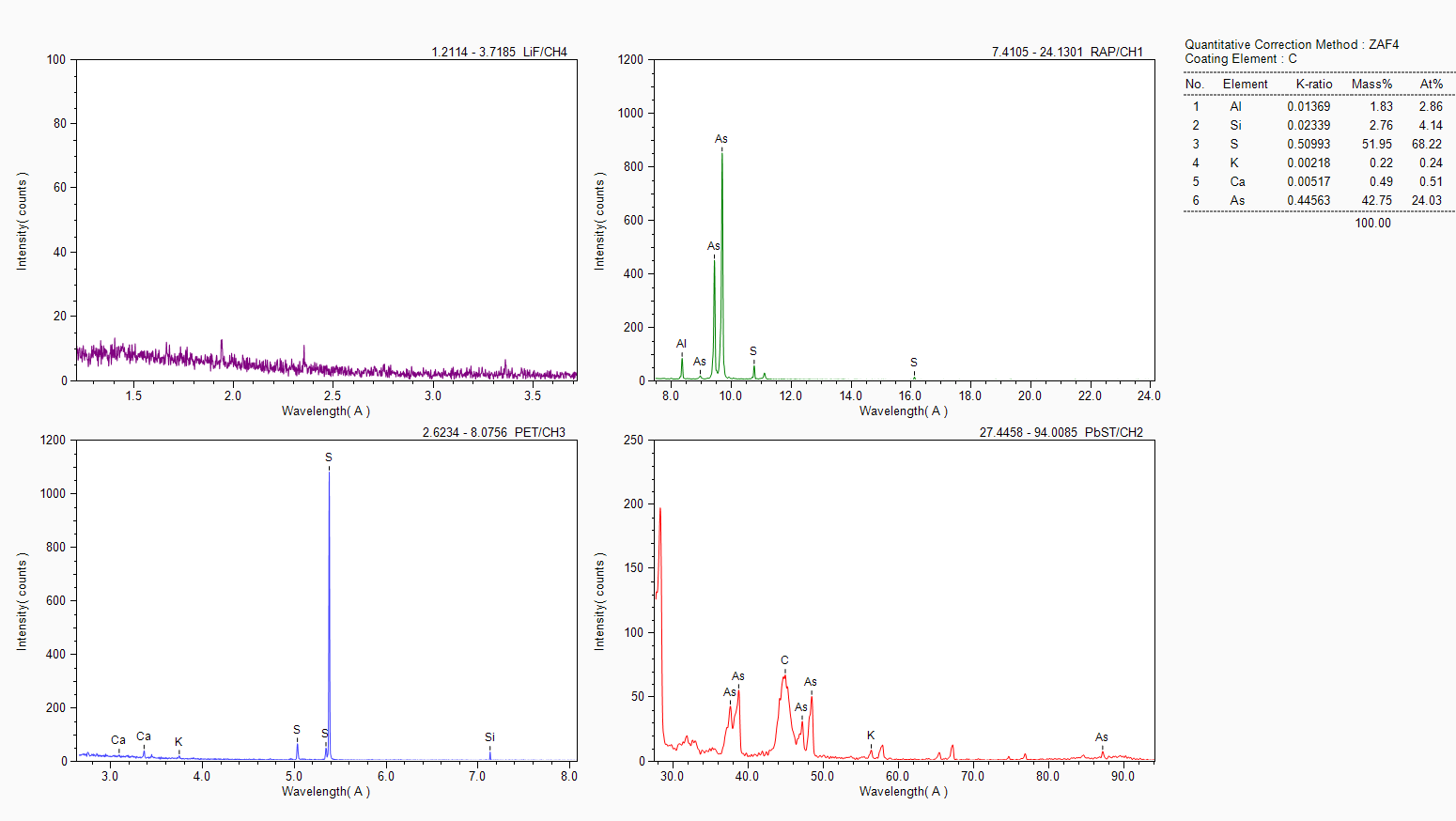

Supplement: Supplementary file 1 [file DataSheet2.zip › Electron Scans/xxy1-10/XH-9qual-2.bmp]

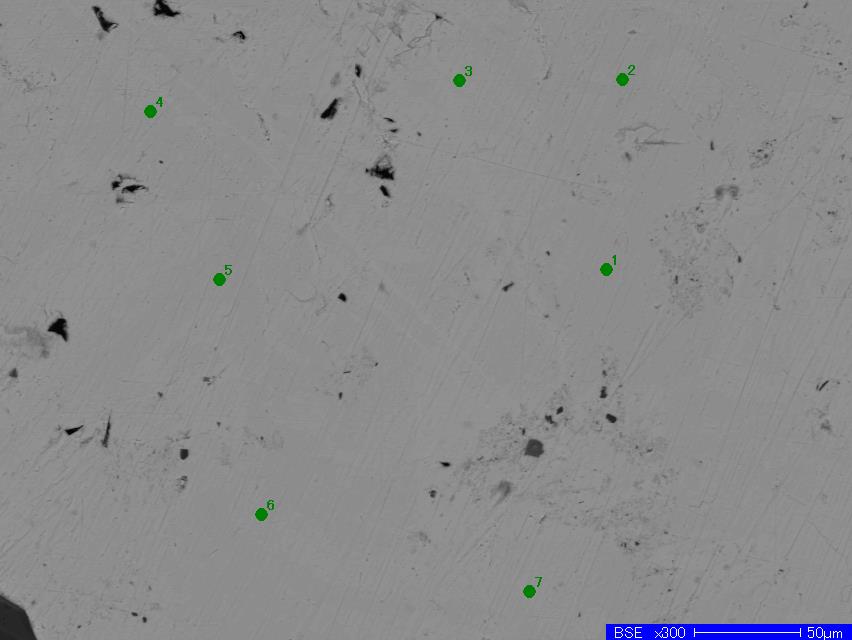

Supplement: Supplementary file 1 [file DataSheet2.zip › Electron Scans/xxy1-10/XH-9quant.jpeg]

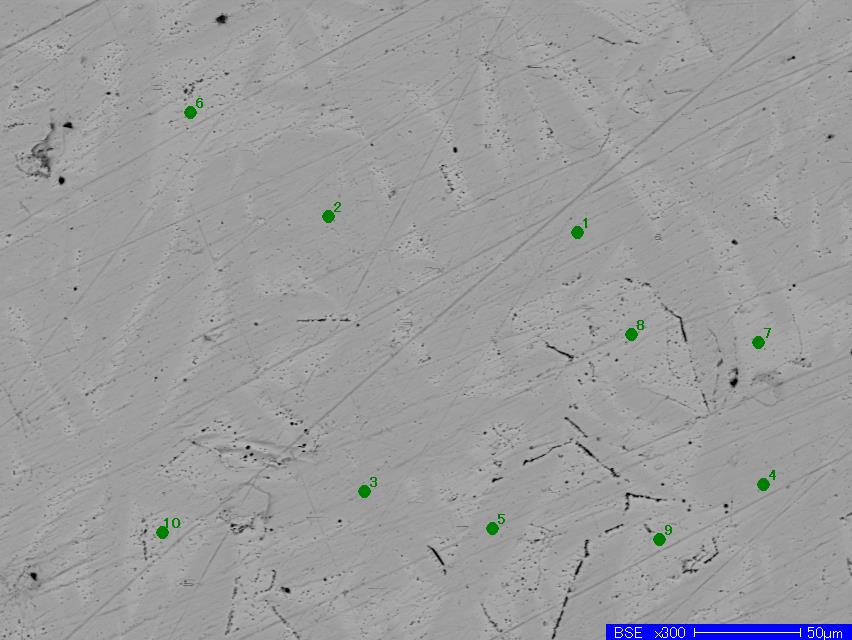

Supplement: Supplementary file 1 [file DataSheet2.zip › Electron Scans/xxy1-10/XH-2-1quant.jpeg]

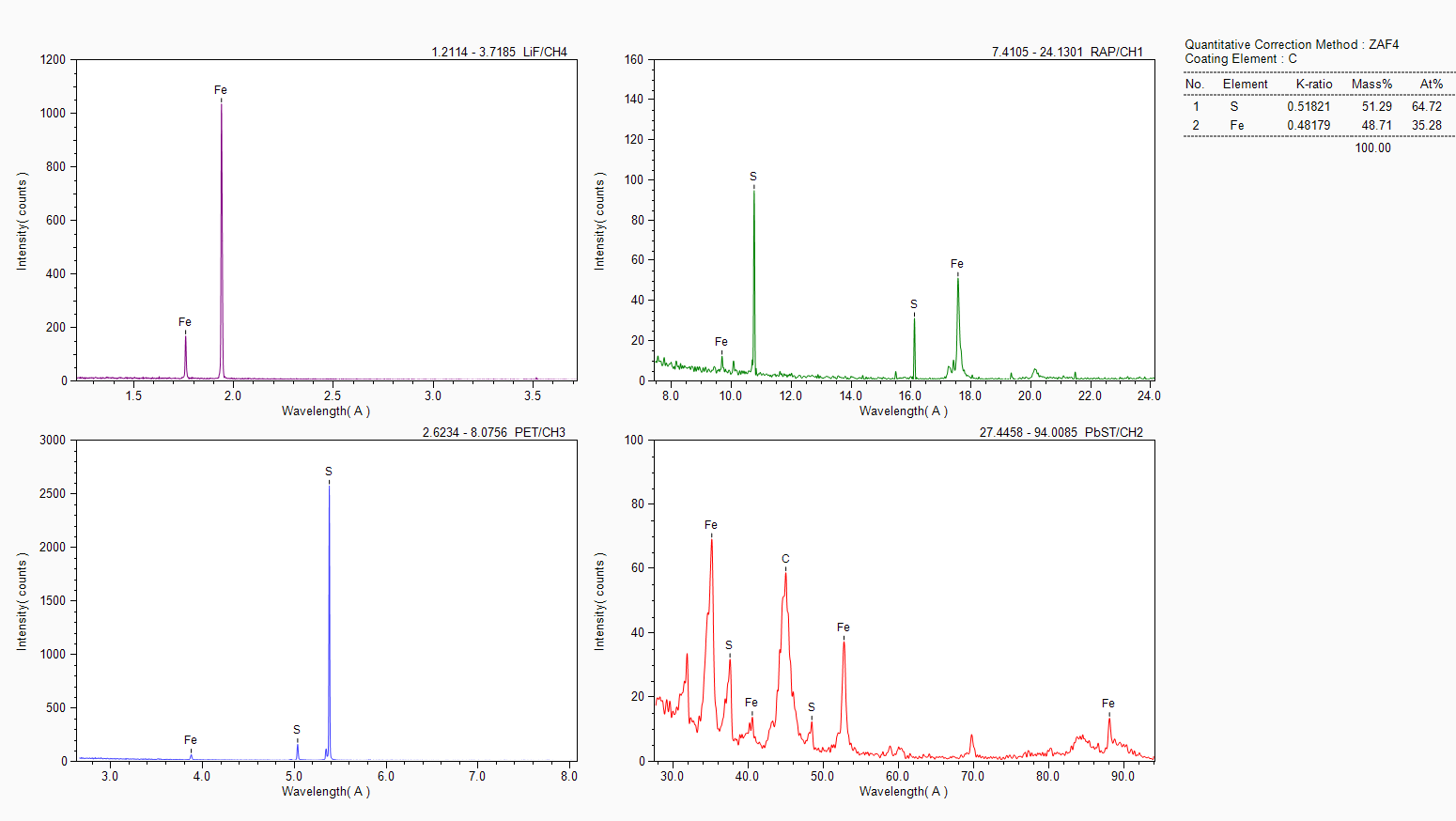

Supplement: Supplementary file 1 [file DataSheet2.zip › Electron Scans/xxy1-10/XH-5-2qual-1.bmp]

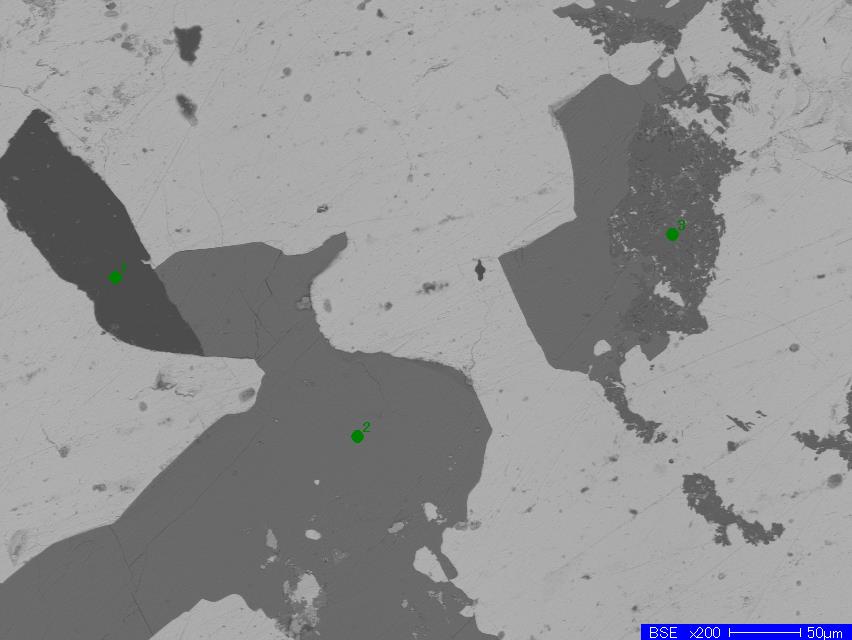

Supplement: Supplementary file 1 [file DataSheet2.zip › Electron Scans/xxy1-10/XH-5-1qual.jpeg]

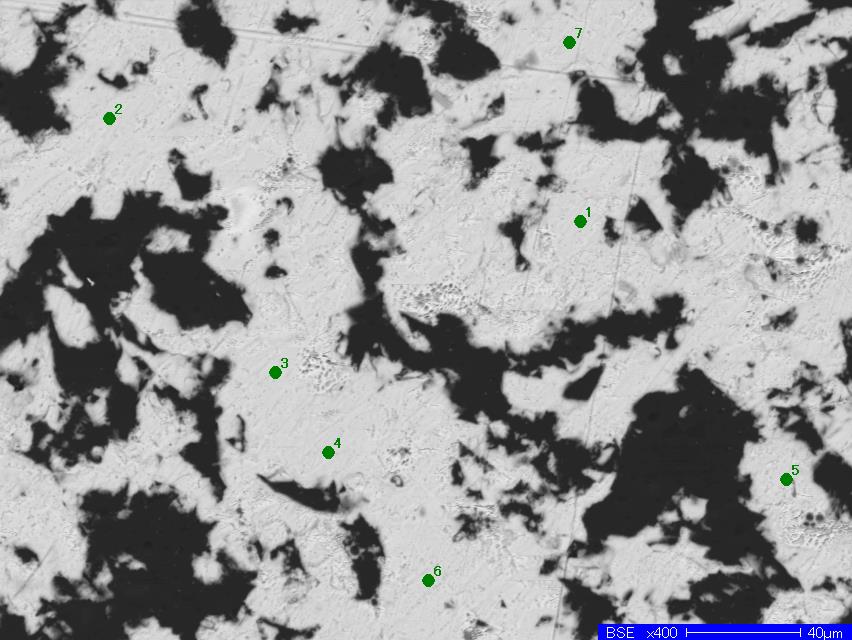

Supplement: Supplementary file 1 [file DataSheet2.zip › Electron Scans/xxy1-10/XH-3quant.jpeg]

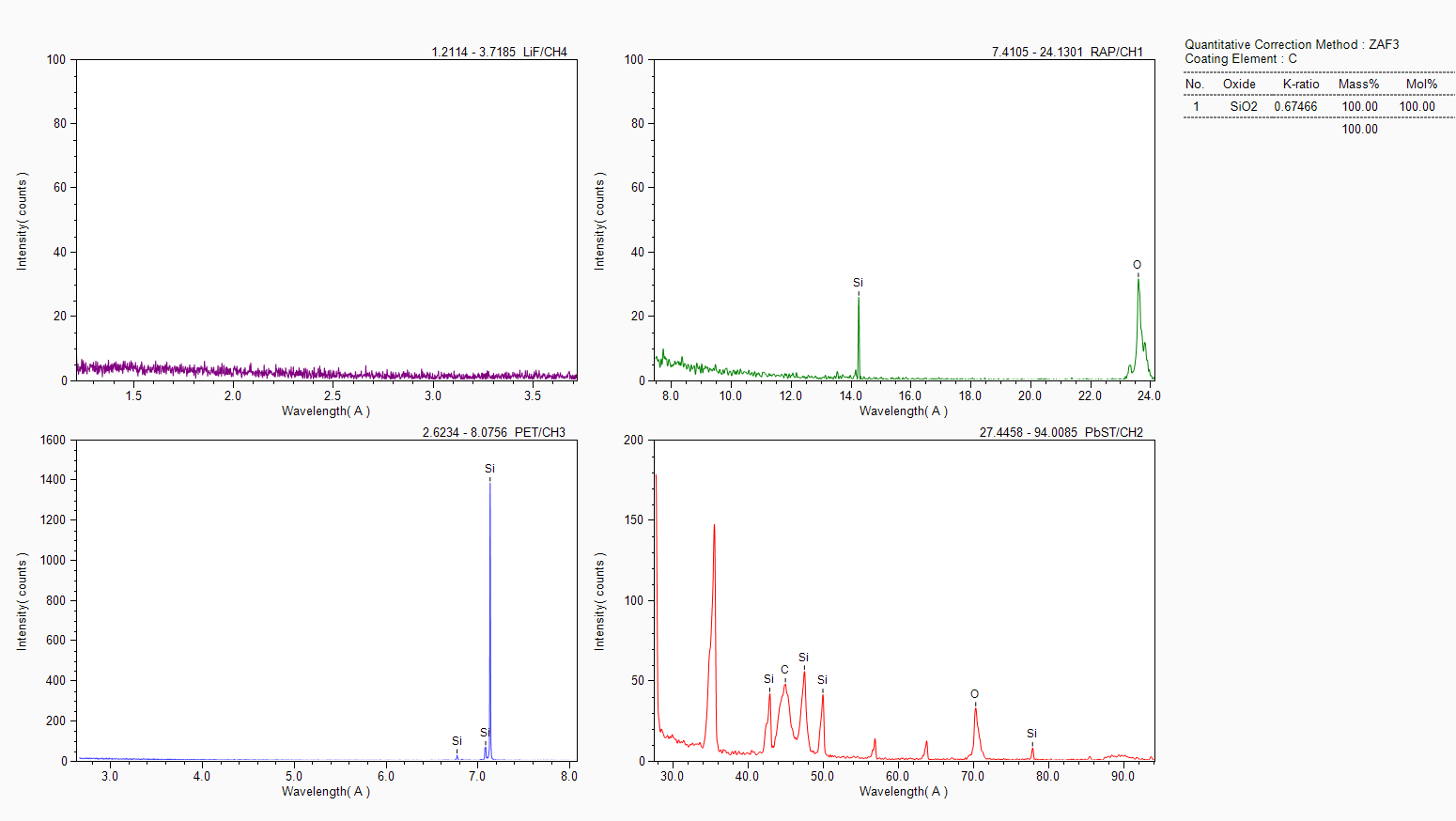

Supplement: Supplementary file 1 [file DataSheet2.zip › Electron Scans/xxy1-10/XH-8qual-1.bmp]

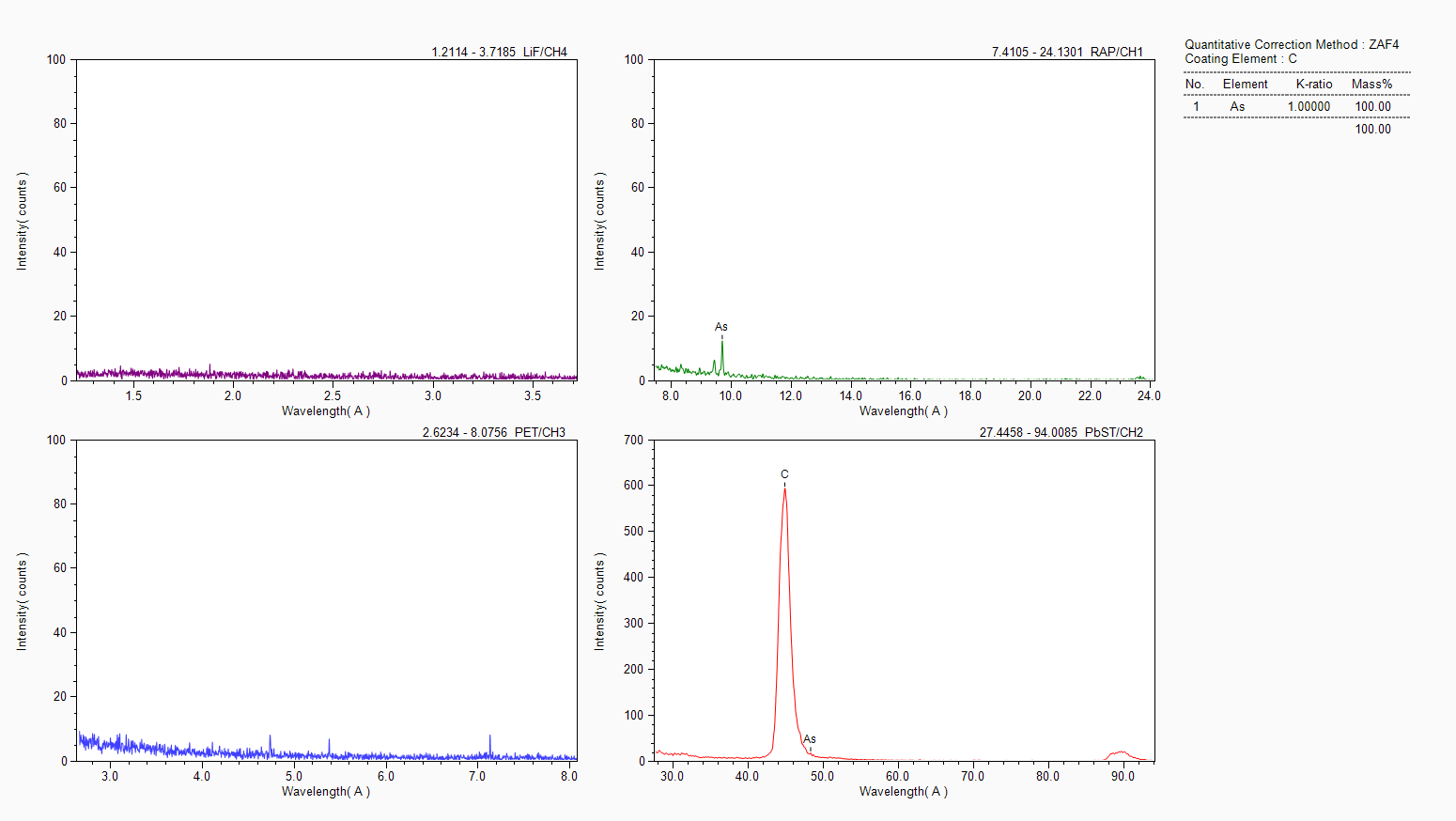

Supplement: Supplementary file 1 [file DataSheet2.zip › Electron Scans/xxy1-10/XH-8qual-2.bmp]

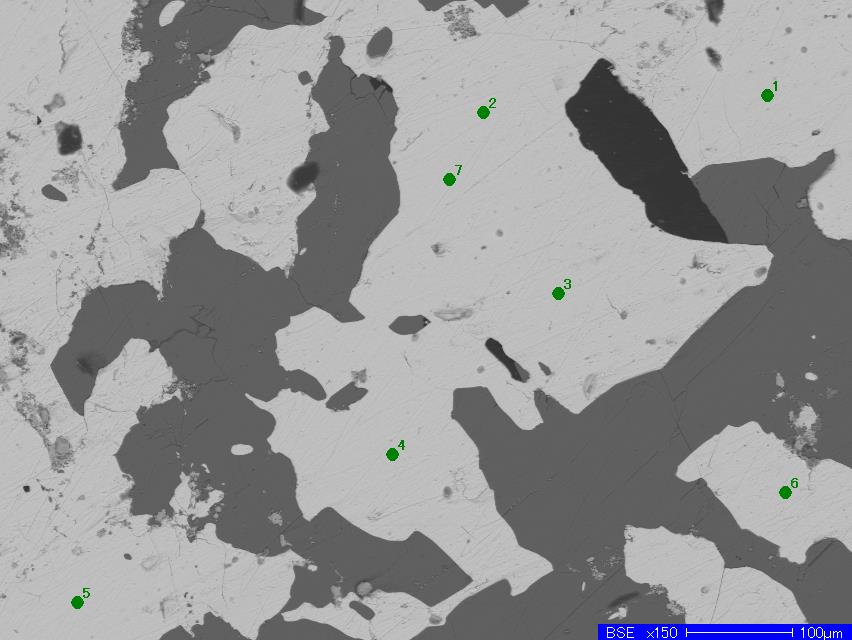

Supplement: Supplementary file 1 [file DataSheet2.zip › Electron Scans/xxy1-10/XH-5quant.jpeg]

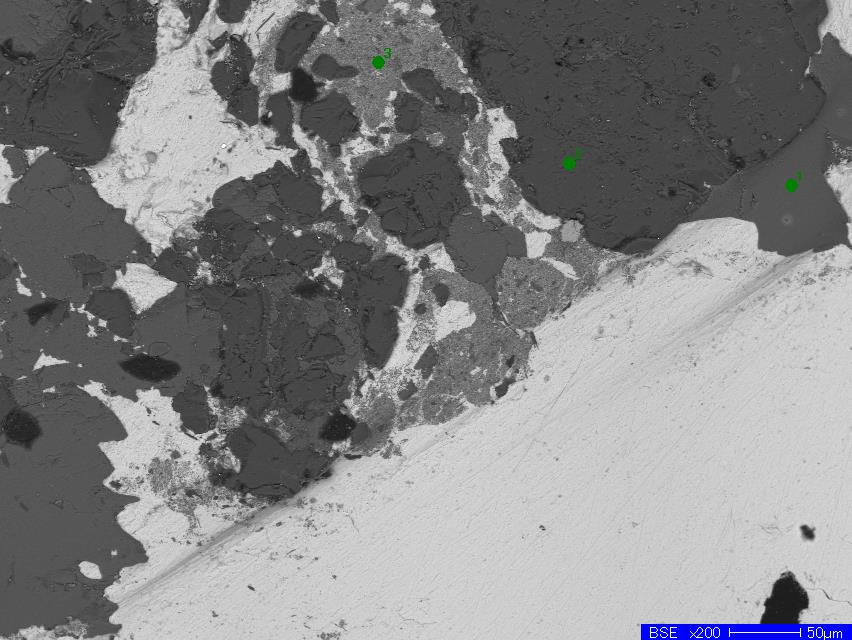

Supplement: Supplementary file 1 [file DataSheet2.zip › Electron Scans/xxy1-10/XH-4qual.jpeg]

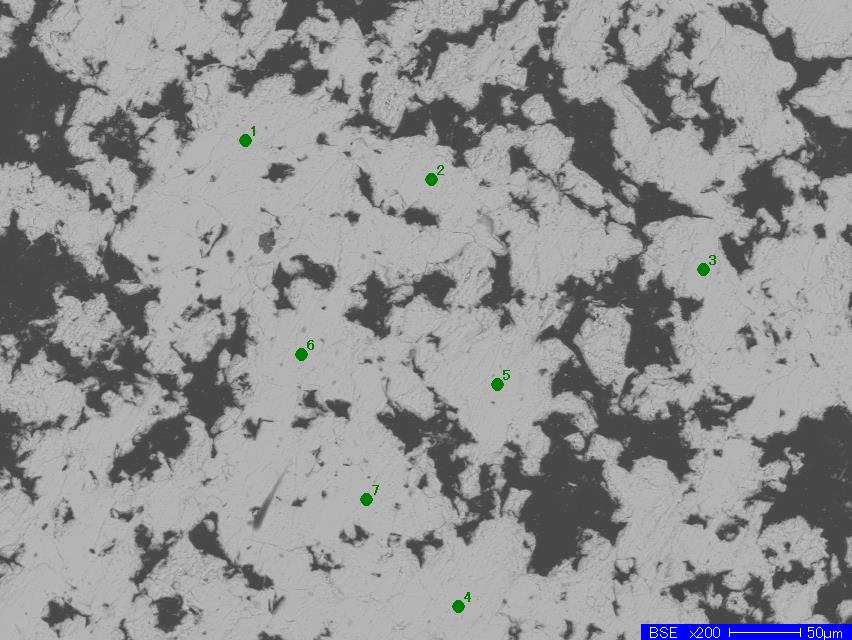

Supplement: Supplementary file 1 [file DataSheet2.zip › Electron Scans/xxy1-10/XH-8quant.jpeg]

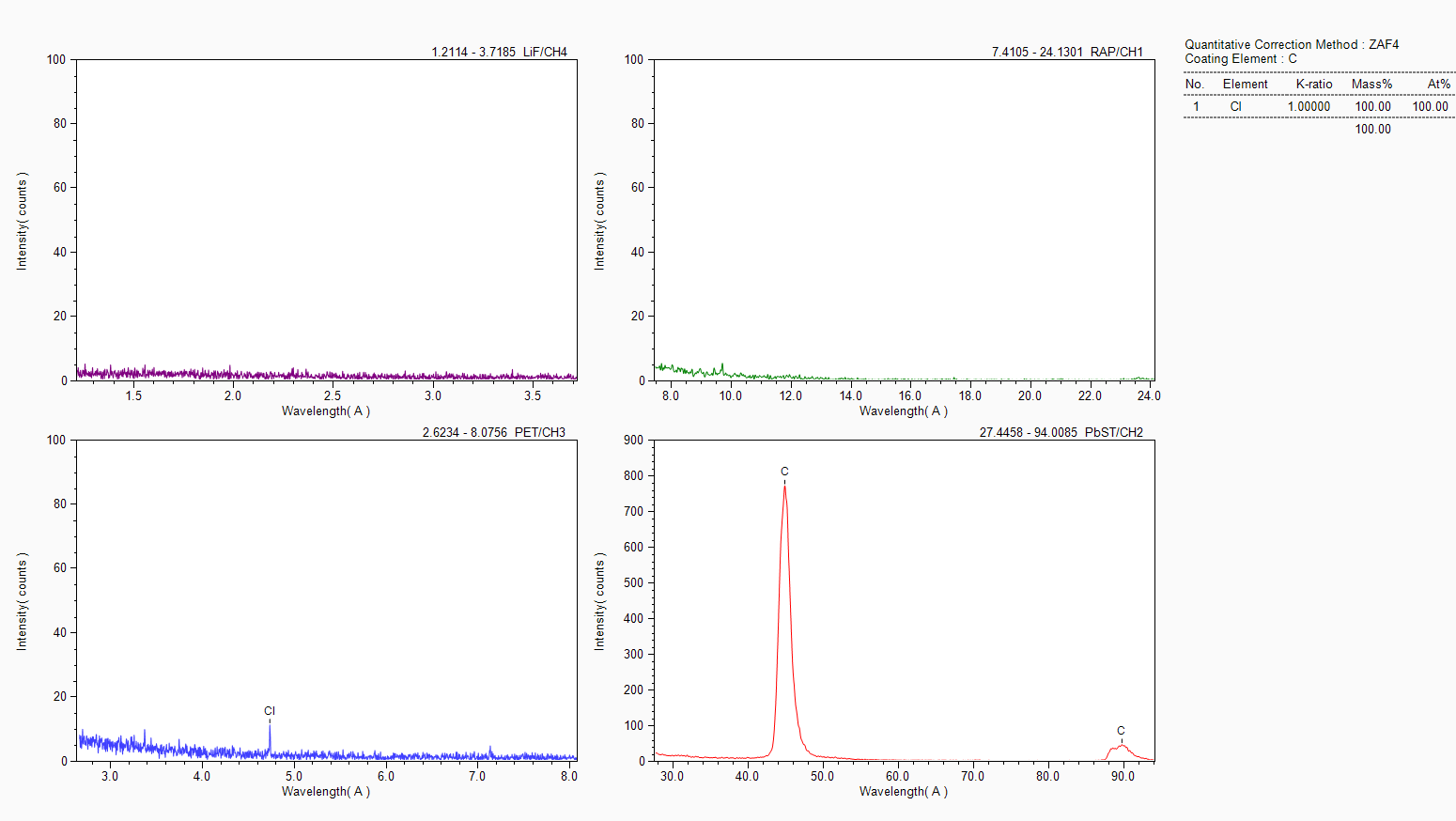

Supplement: Supplementary file 1 [file DataSheet2.zip › Electron Scans/xxy1-10/XH-5-1qual-1.bmp]

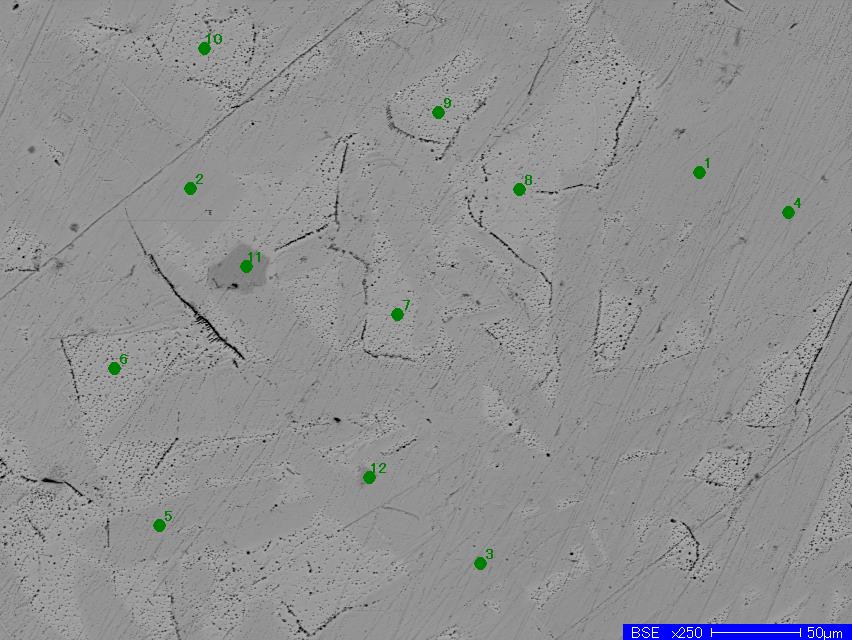

Supplement: Supplementary file 1 [file DataSheet2.zip › Electron Scans/xxy1-10/XH-2-2quant.jpeg]

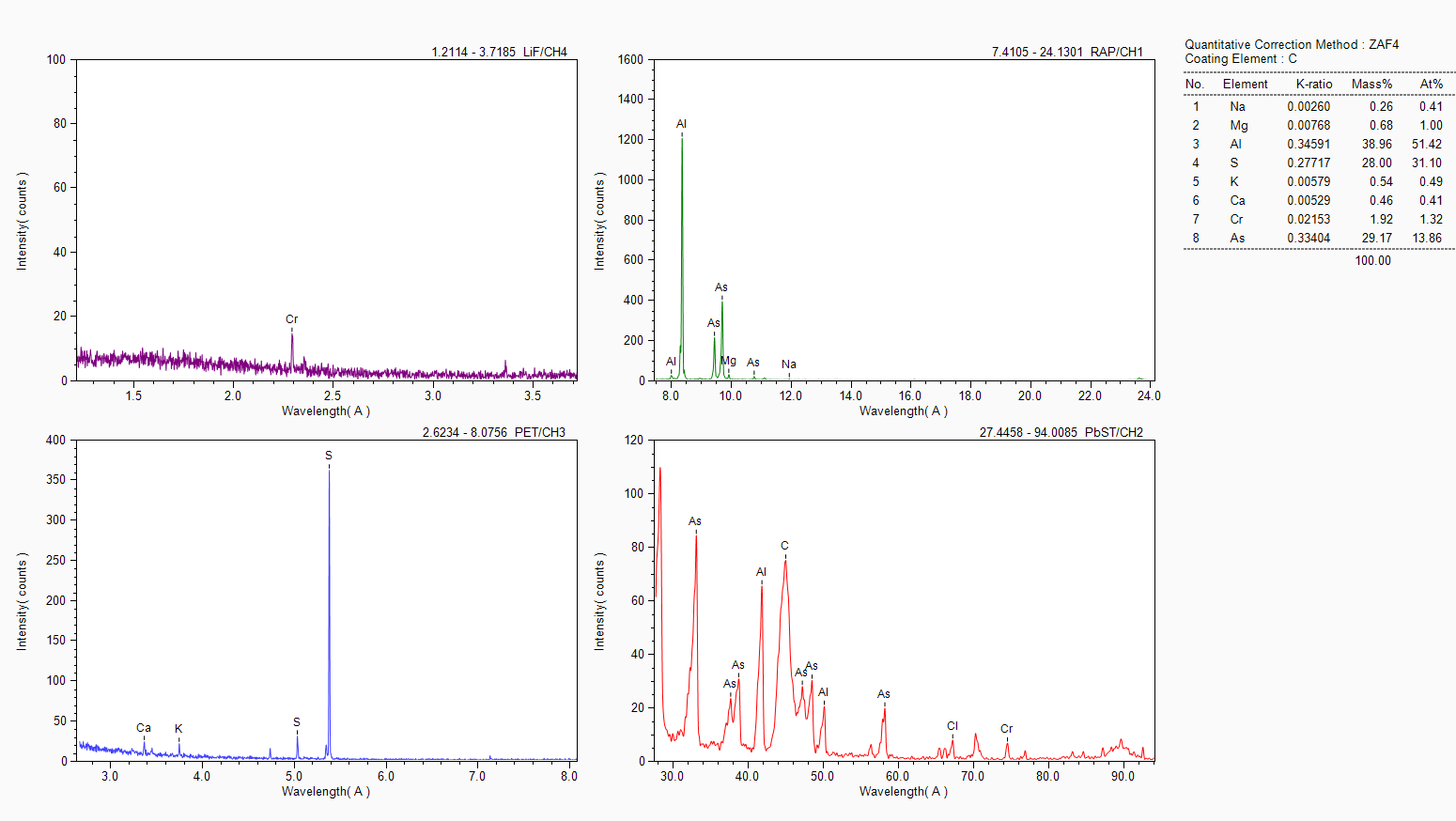

Supplement: Supplementary file 1 [file DataSheet2.zip › Electron Scans/xxy1-10/XH-6qual-1.bmp]

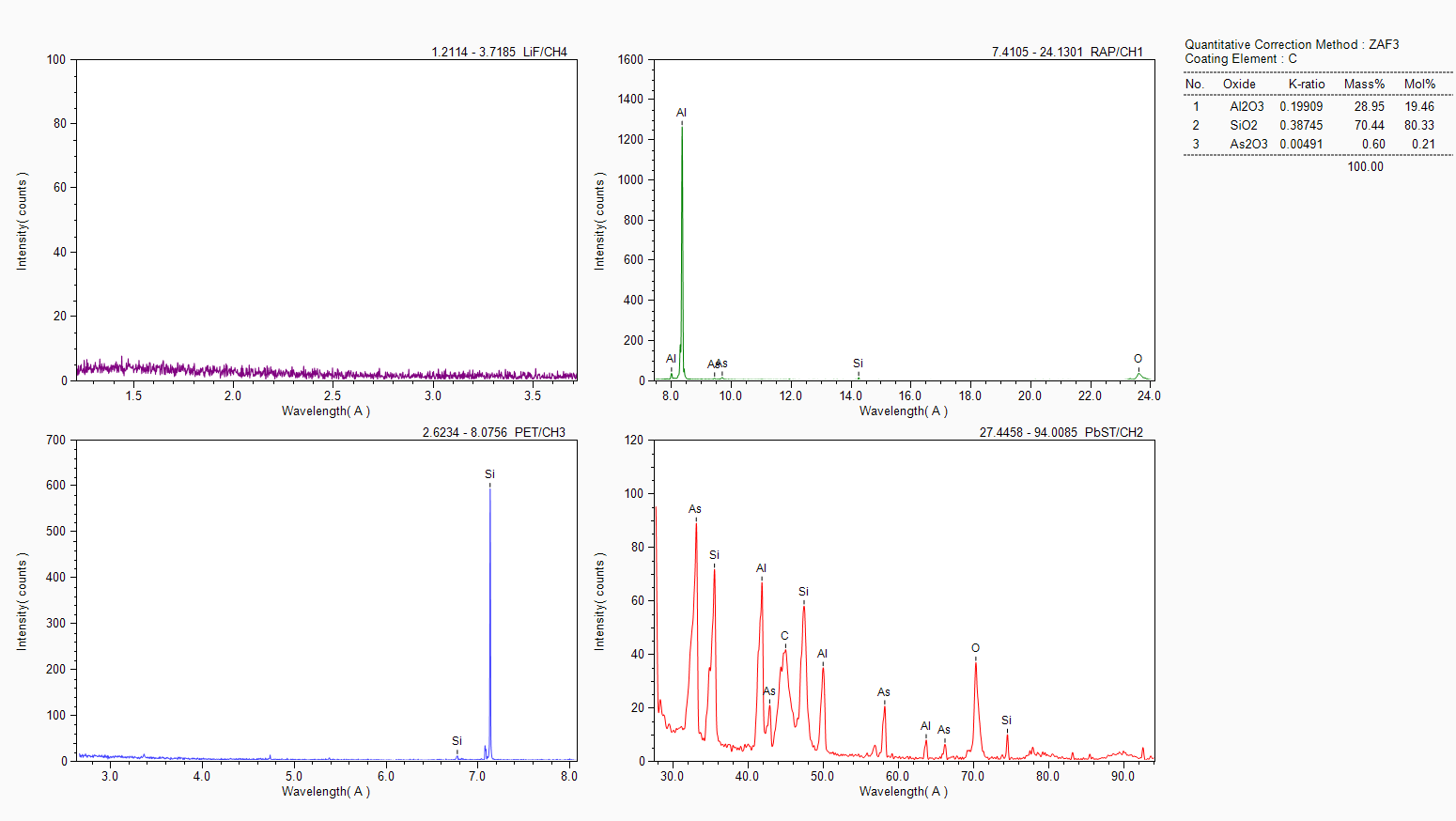

Supplement: Supplementary file 1 [file DataSheet2.zip › Electron Scans/xxy1-10/XH-5-1qual-3.bmp]

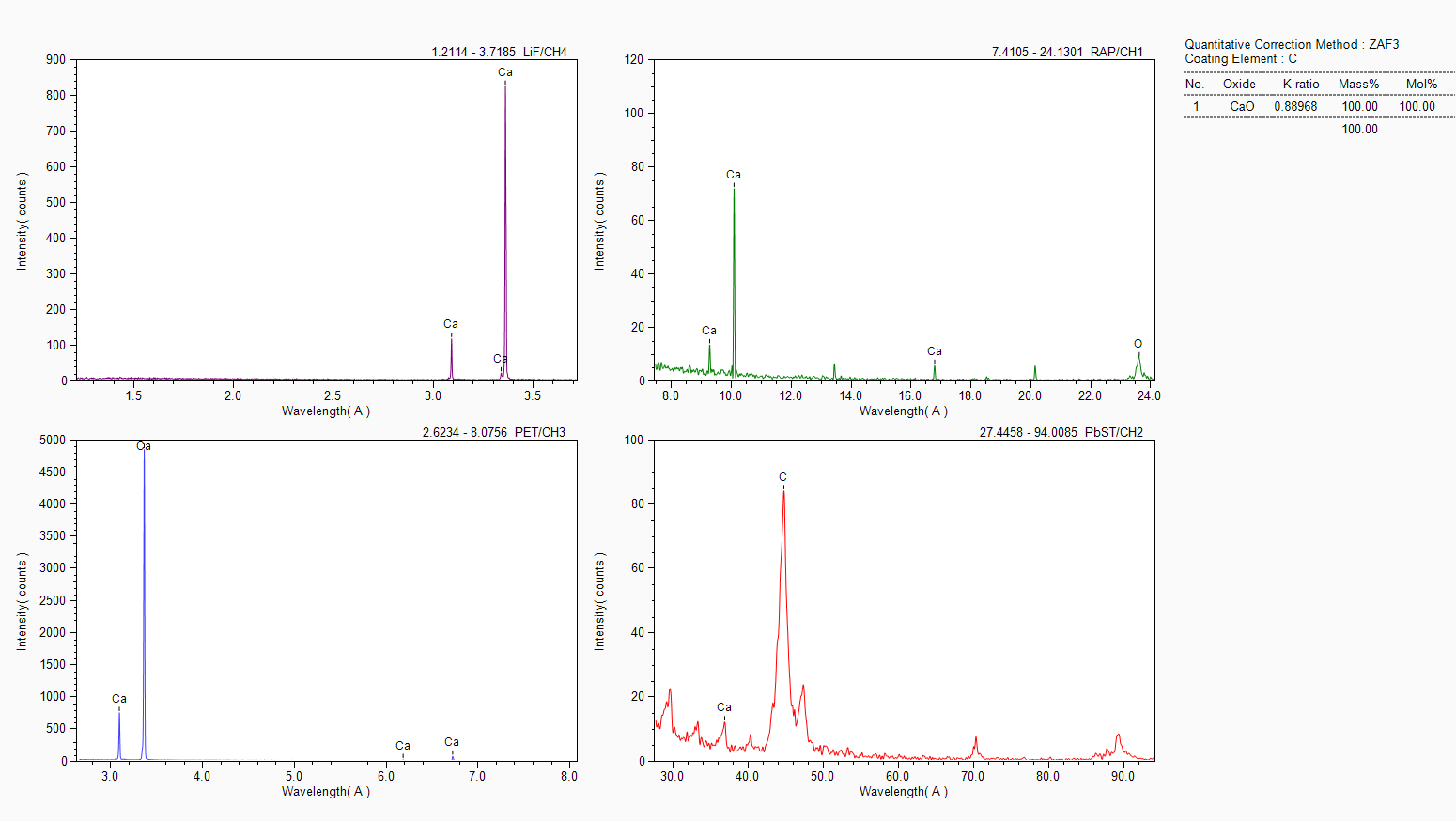

Supplement: Supplementary file 1 [file DataSheet2.zip › Electron Scans/xxy1-10/XH-5-1qual-2.bmp]

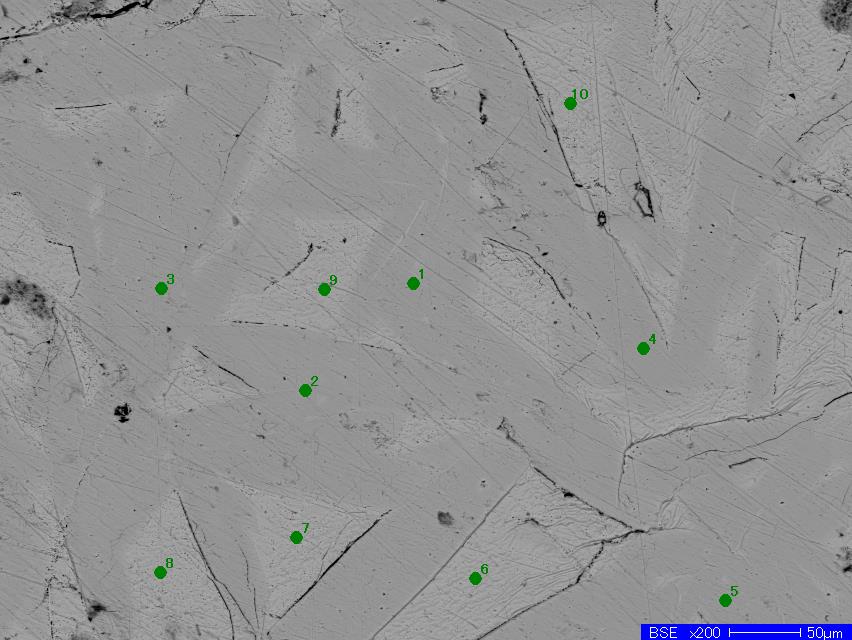

Supplement: Supplementary file 1 [file DataSheet2.zip › Electron Scans/xxy1-10/XH-6quant.jpeg]

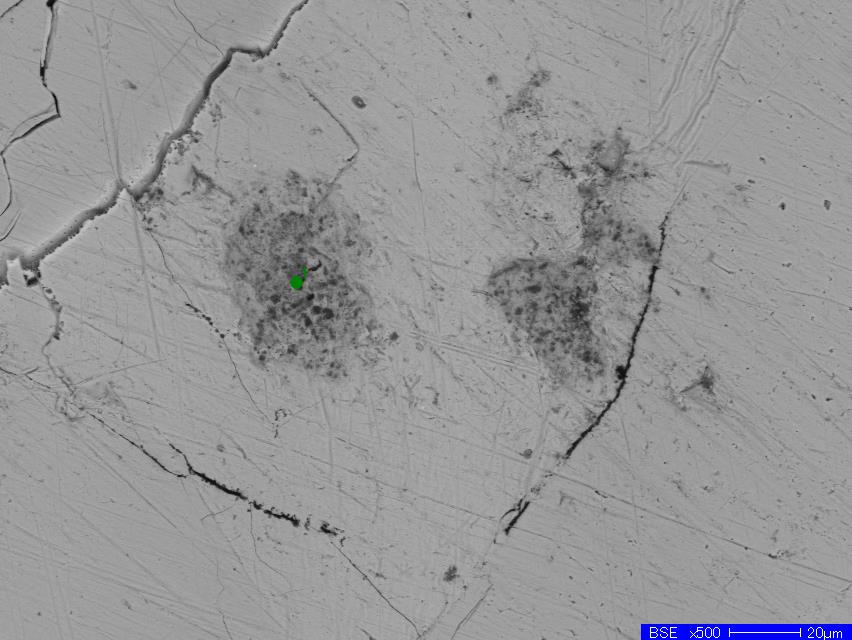

Supplement: Supplementary file 1 [file DataSheet2.zip › Electron Scans/xxy1-10/XH-6qual.jpeg]

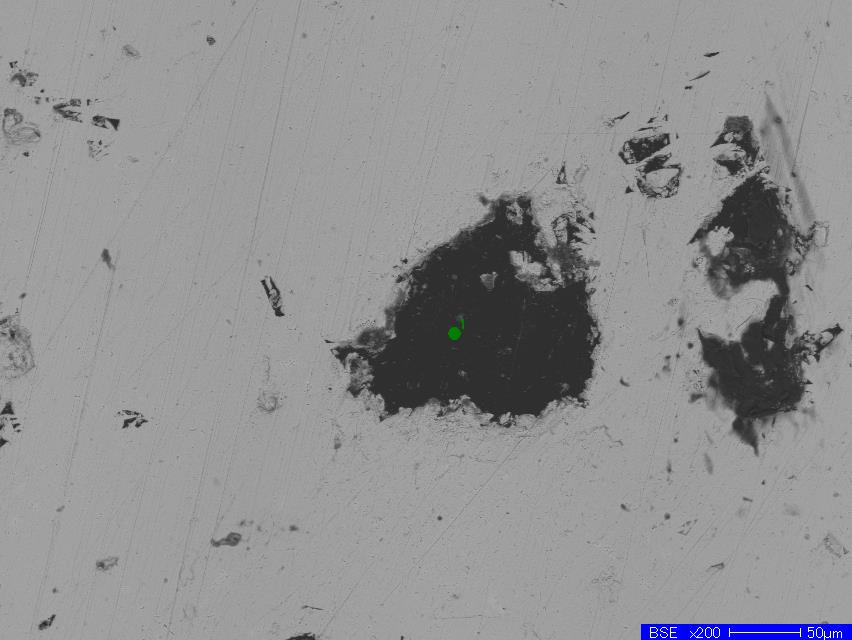

Supplement: Supplementary file 1 [file DataSheet2.zip › Electron Scans/xxy1-10/XH-7qual.jpeg]

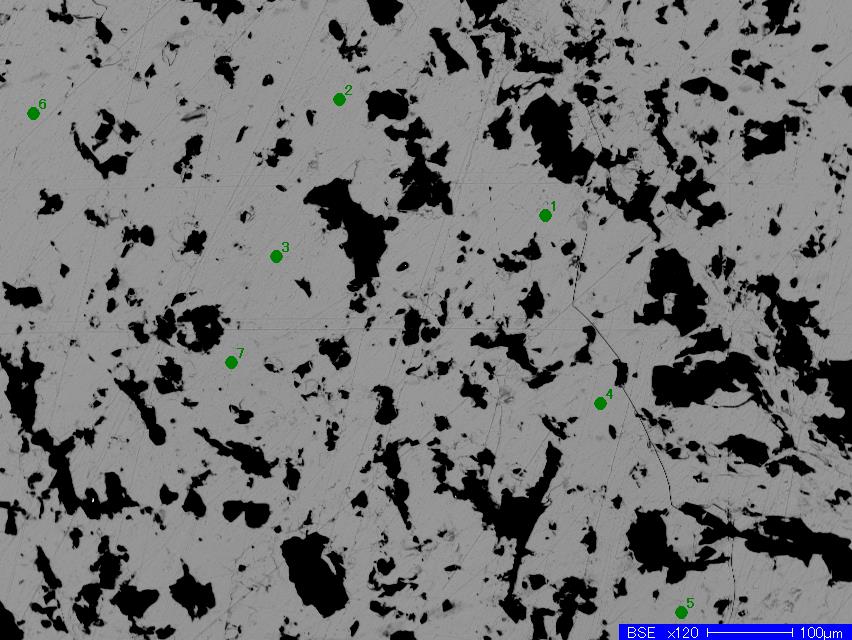

Supplement: Supplementary file 1 [file DataSheet2.zip › Electron Scans/xxy1-10/XH-1quant.jpeg]

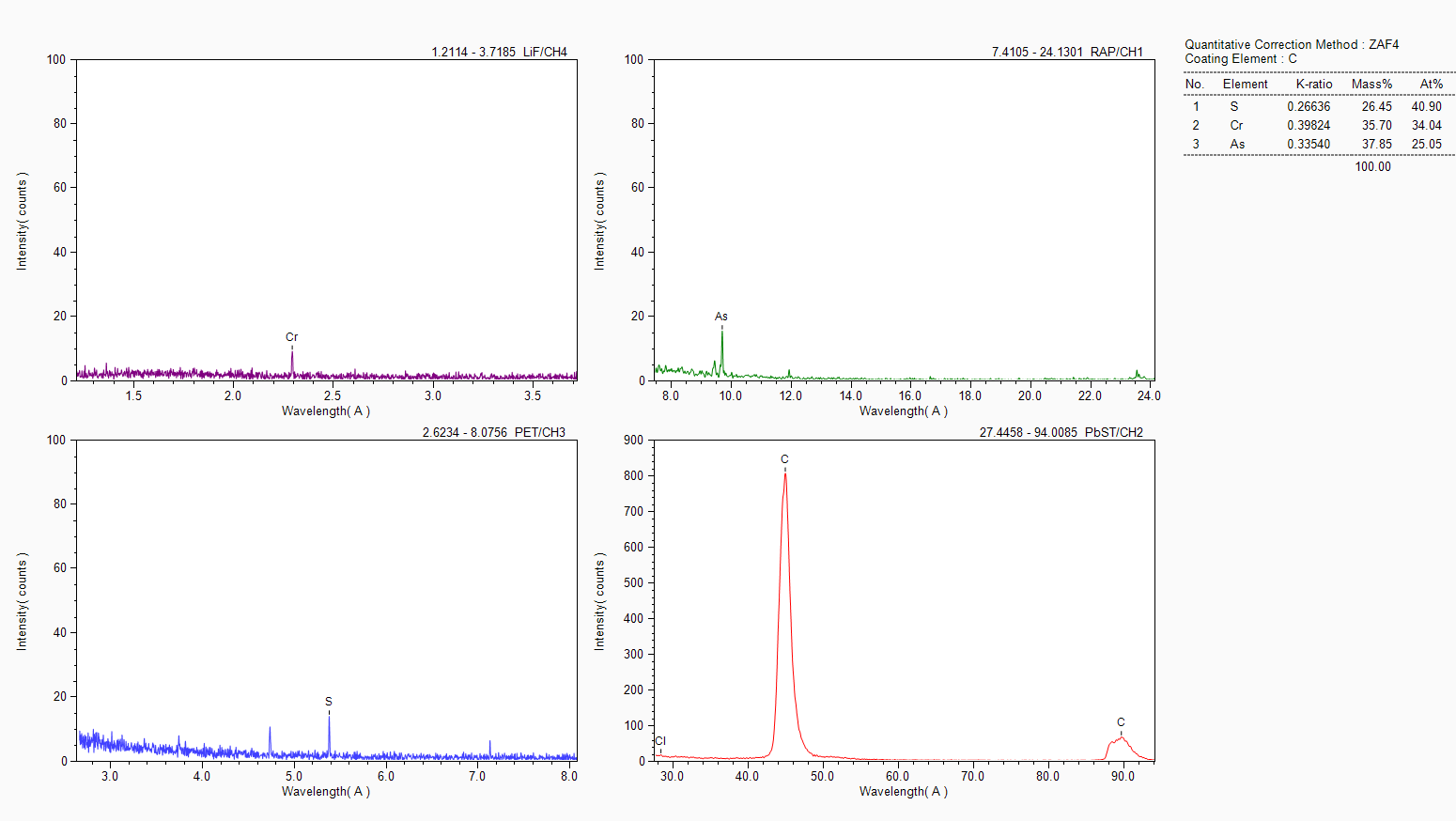

Supplement: Supplementary file 1 [file DataSheet2.zip › Electron Scans/xxy1-10/XH-10-1qual-2.bmp]

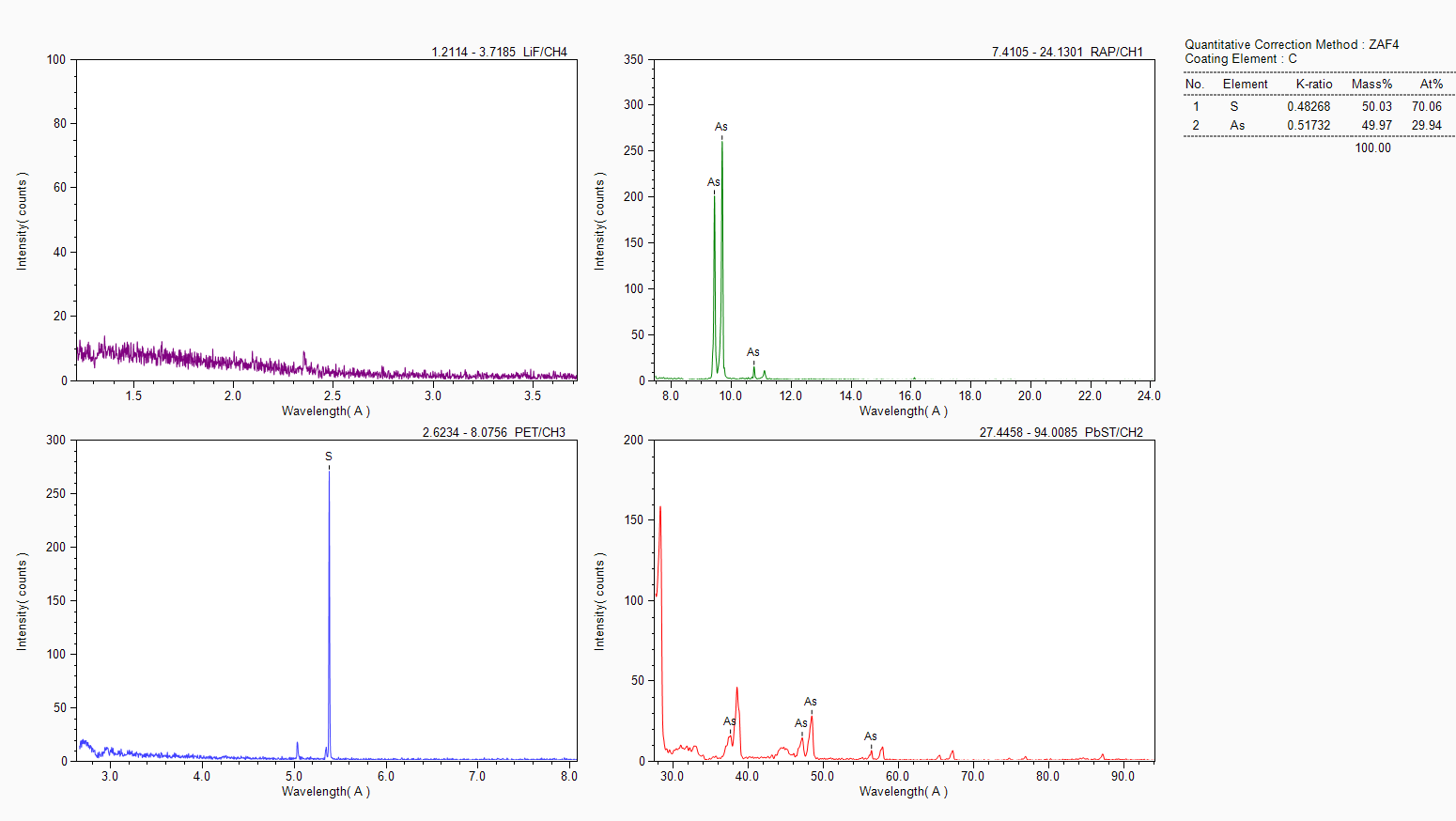

Supplement: Supplementary file 1 [file DataSheet2.zip › Electron Scans/xxy1-10/XH-10-1qual-1.bmp]

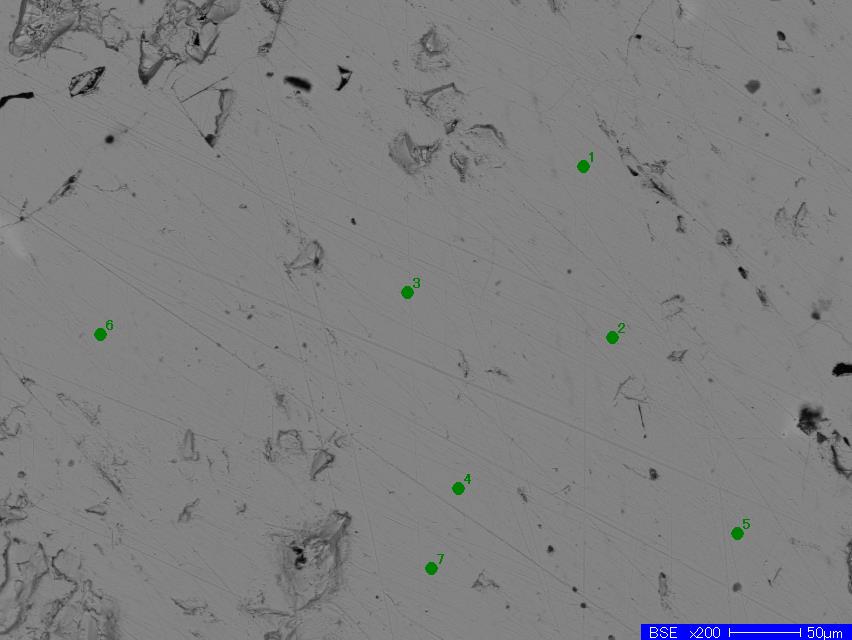

Supplement: Supplementary file 1 [file DataSheet2.zip › Electron Scans/xxy1-10/XH-10-1quant.jpeg]

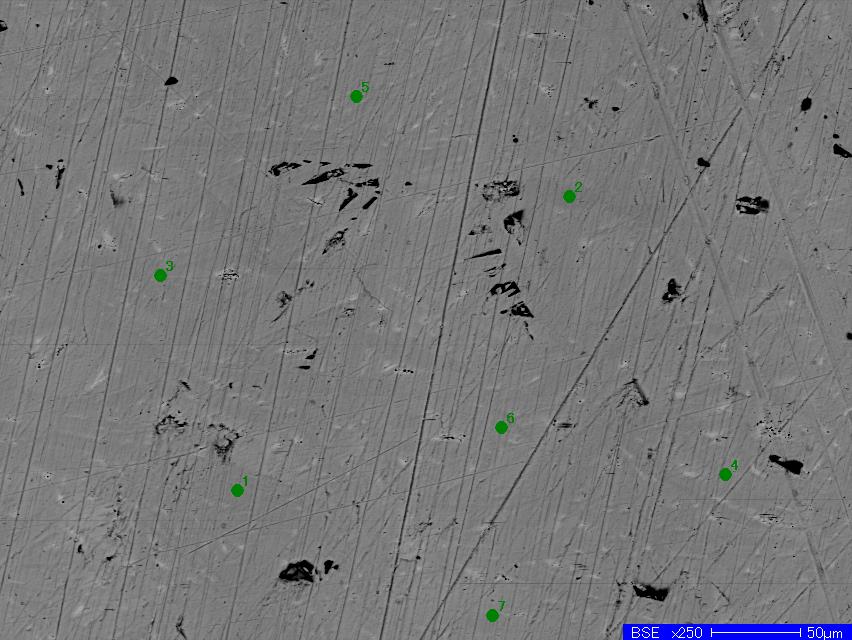

Supplement: Supplementary file 1 [file DataSheet2.zip › Electron Scans/xxy1-10/XH-7quant.jpeg]

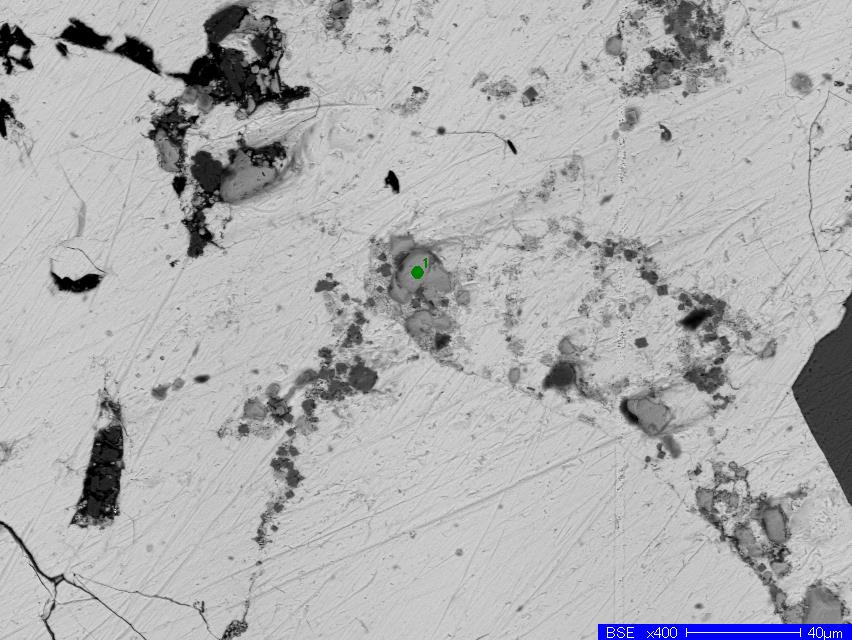

Supplement: Supplementary file 1 [file DataSheet2.zip › Electron Scans/xxy1-10/XH-5-2qual.jpeg]
