# Supplementary material for: Characterization and comparation of toxicity between natural realgar and artificially optimized realgar
Source: Front Pharmacol. 2024 Oct 28;15:1476139. doi: 10.3389/fphar.2024.1476139 (PMC11550961; doi:10.3389/fphar.2024.1476139)
Supplement: Supplementary file 3 [file DataSheet1.docx]

Realgar samples information statistics table

Number Sample Location

XH-01 medicinal material samples Bozhou, Anhui Province, China

XH-02 medicinal material samples Bozhou, Anhui Province, China

XH-03 medicinal material samples Sichuan Province, China

XH-04 medicinal material samples Sichuan Province, China

XH-05 medicinal material samples Shimen, Hunan Province, China

XH-06 medicinal material samples Guizhou Province, China

XH-07 medicinal material samples Bozhou, Anhui Province, China

XH-08 medicinal material samples Bozhou, Anhui Province, China

XH-09 medicinal material samples Shimen, Hunan Province, China

XH-10 medicinal material samples Chatian, Hunan Province, China

XH-11 medicinal material samples Shimen, Hunan Province, China

XH-12 medicinal material samples Shimen, Hunan Province, China

XH-13 medicinal material samples Guangxi Province, China

XH-14 medicinal material samples Wanshan, Guizhou Province, China

XH-15 medicinal material samples Guizhou Province, China

XH-16 medicinal material samples Guizhou Province, China

XH-17 medicinal material samples Anhui Province, China

XH-18 medicinal material samples Yueyang, Hunan Province, China

XH-19 medicinal material samples Yunnan Province, China

XH-20 medicinal material samples Bozhou, Anhui Province, China

XH-21 medicinal material samples Hebei Province, China

XH-22 medicinal material samples Hebei Province, China

XH-23 medicinal material samples Hebei Province, China

XH-24 medicinal material samples Brazil

XH-25 realgar processed products Beijing, China

XH-26 realgar processed products Beijing, China

XH-27 realgar processed products Beijing, China

XH-28 realgar processed products Henan Province, China

XH-29 realgar processed products Guangdong Province, China

XH-30 realgar processed products Henan Province, China

Supplement table 1: Realgar samples information statistics table.

| Reagent Name | Batch Number | Company |
| --- | --- | --- |
| Dilute Hydrochloric Acid | 7647-01-0 | Nanjing Chemical Reagent Company |
| Trypsin | 8049-47-6 | Sigma, USA |
| Pepsin | 9001-75-6 | Sigma, USA |
| Sodium Hydroxide | 1310-73-2 | Sigma, USA |
| Arsenobetaine Solution Standard | GBW08670 | National Institute of Metrology, China |
| Arsenocholine Solution Standard | GBW08671 | National Institute of Metrology, China |
| Arsenate Solution Standard | GBW08667 | National Institute of Metrology, China |
| Arsenite Solution Standard | GBW08666 | National Institute of Metrology, China |
| Monomethylarsonic Acid Solution Standard | GBW08668 | National Institute of Metrology, China |
| Dimethylarsinic Acid Solution Standard | GBW08669 | National Institute of Metrology, China |

Supplement table 2: Preparation of different reagents.

**
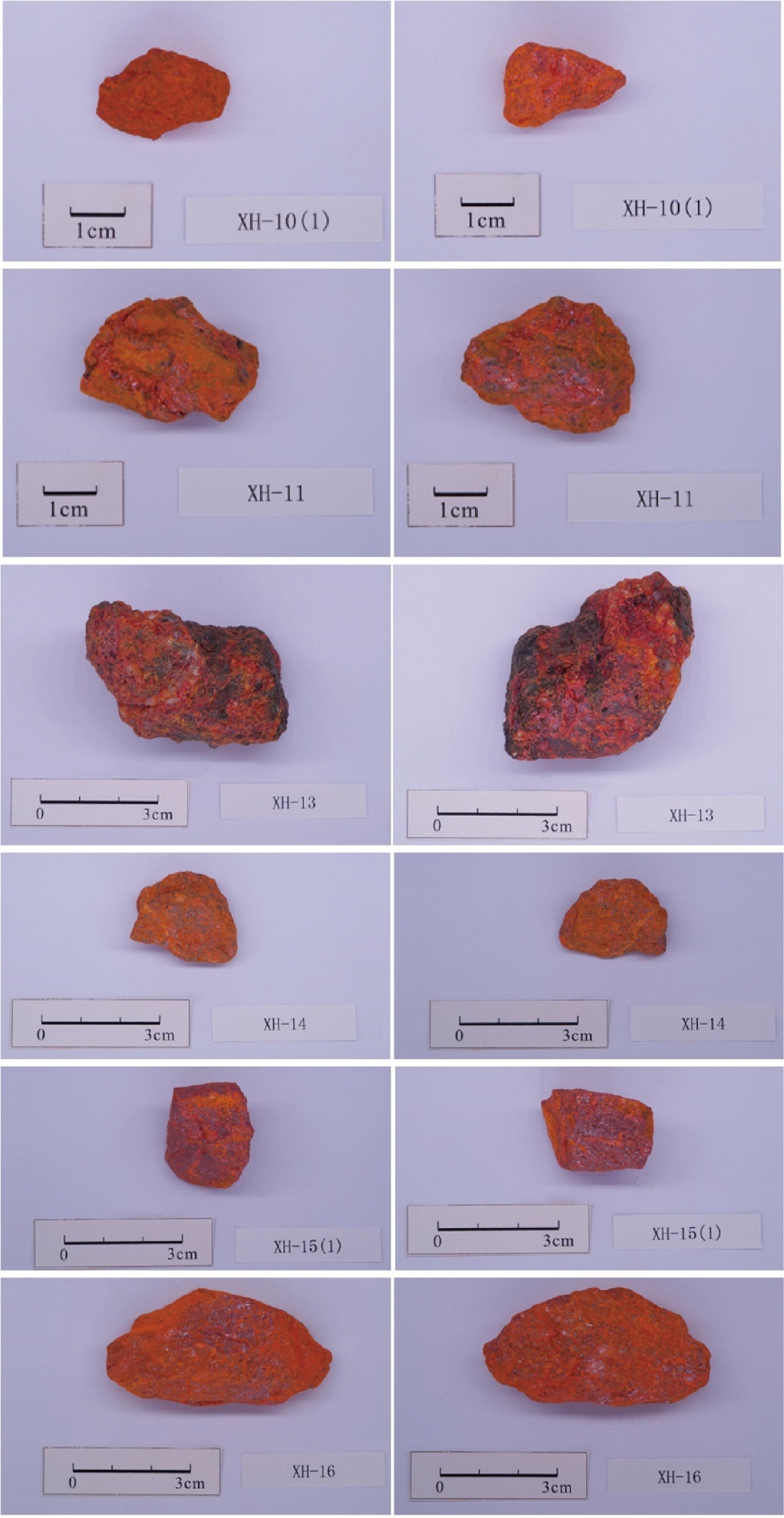
**


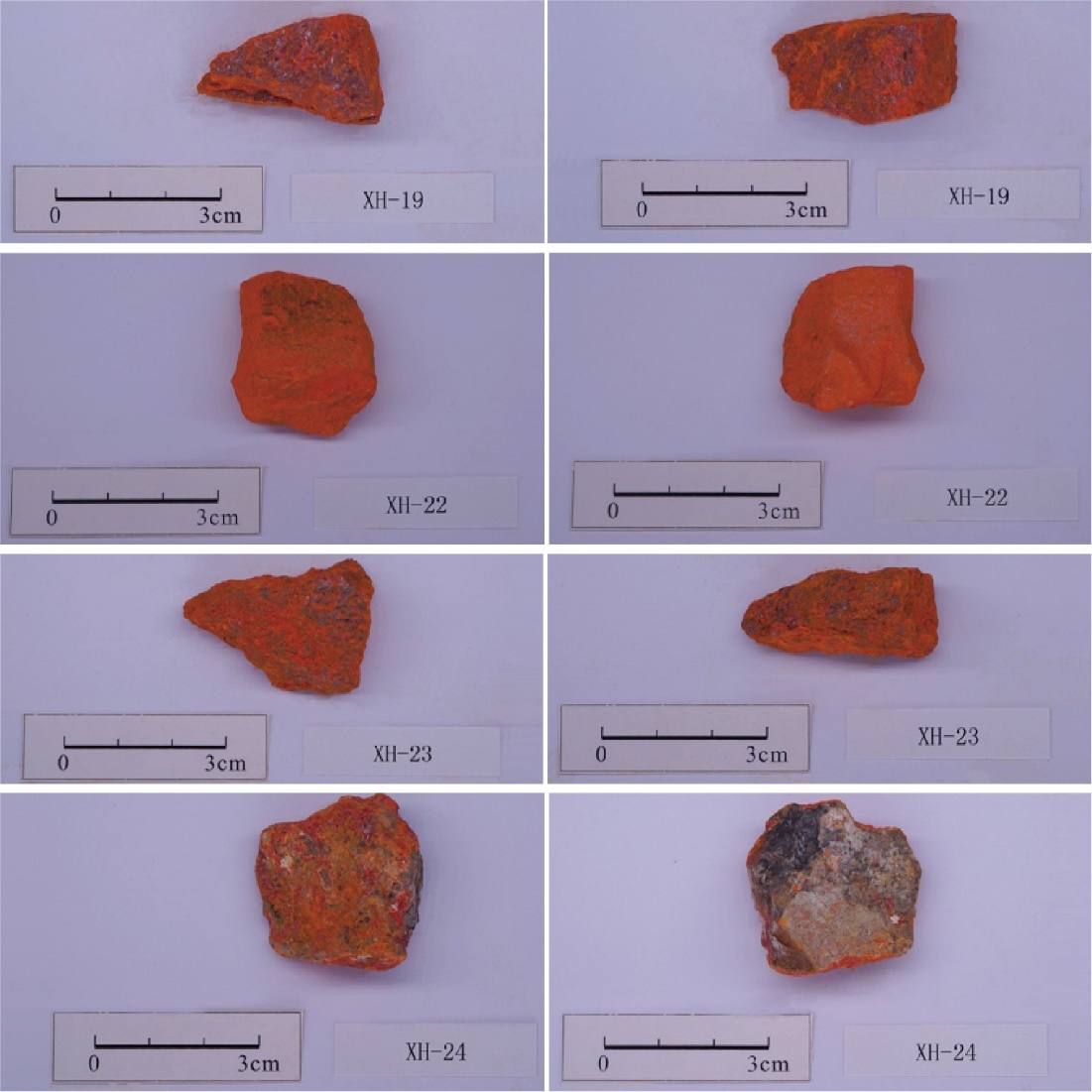


Supplement Figure 1. The identification of characteristic feature in realgar processed products


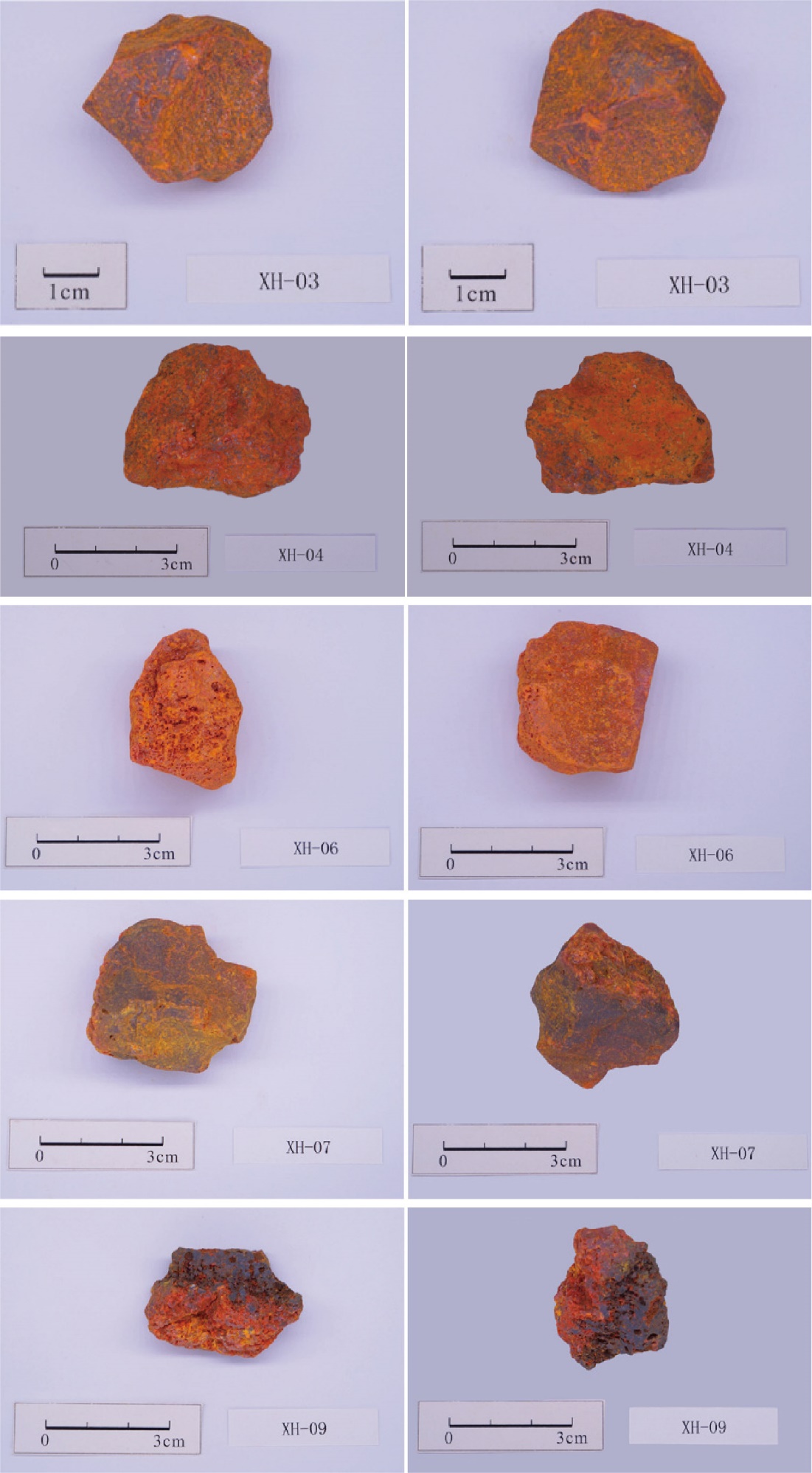


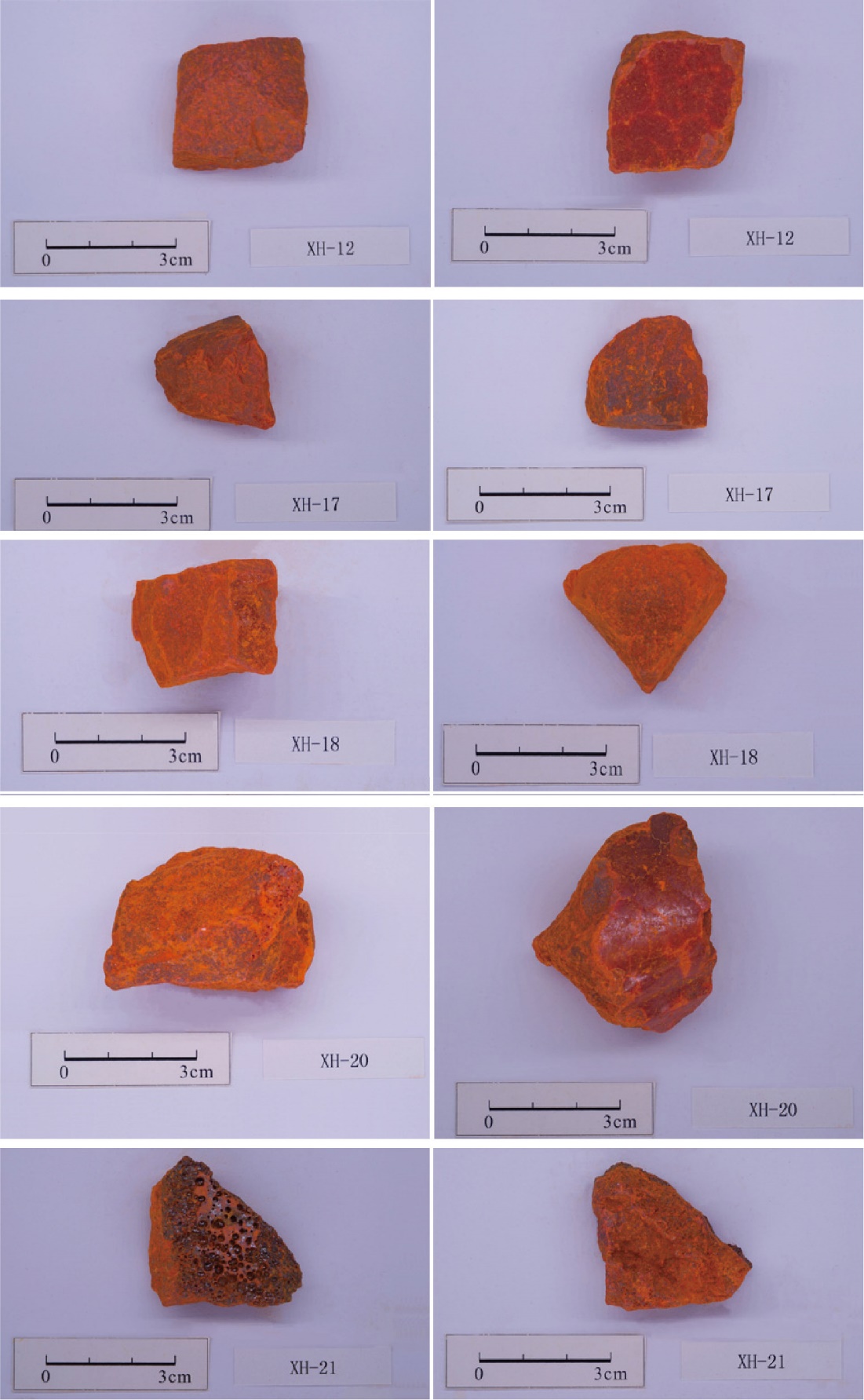


Supplement Figure 2: The identification of characteristic feature in artificially optimized realgar or realgar processed products.


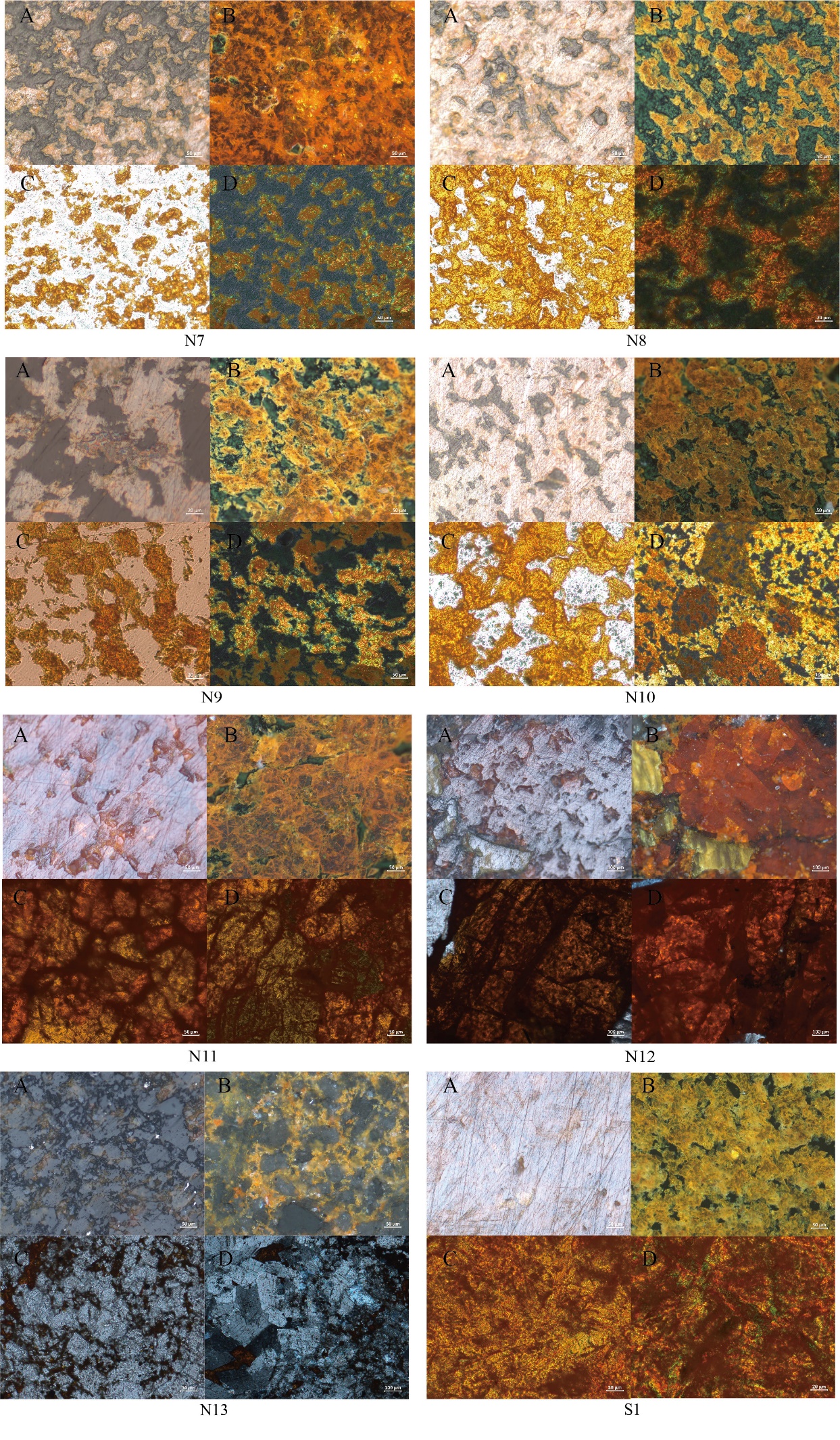

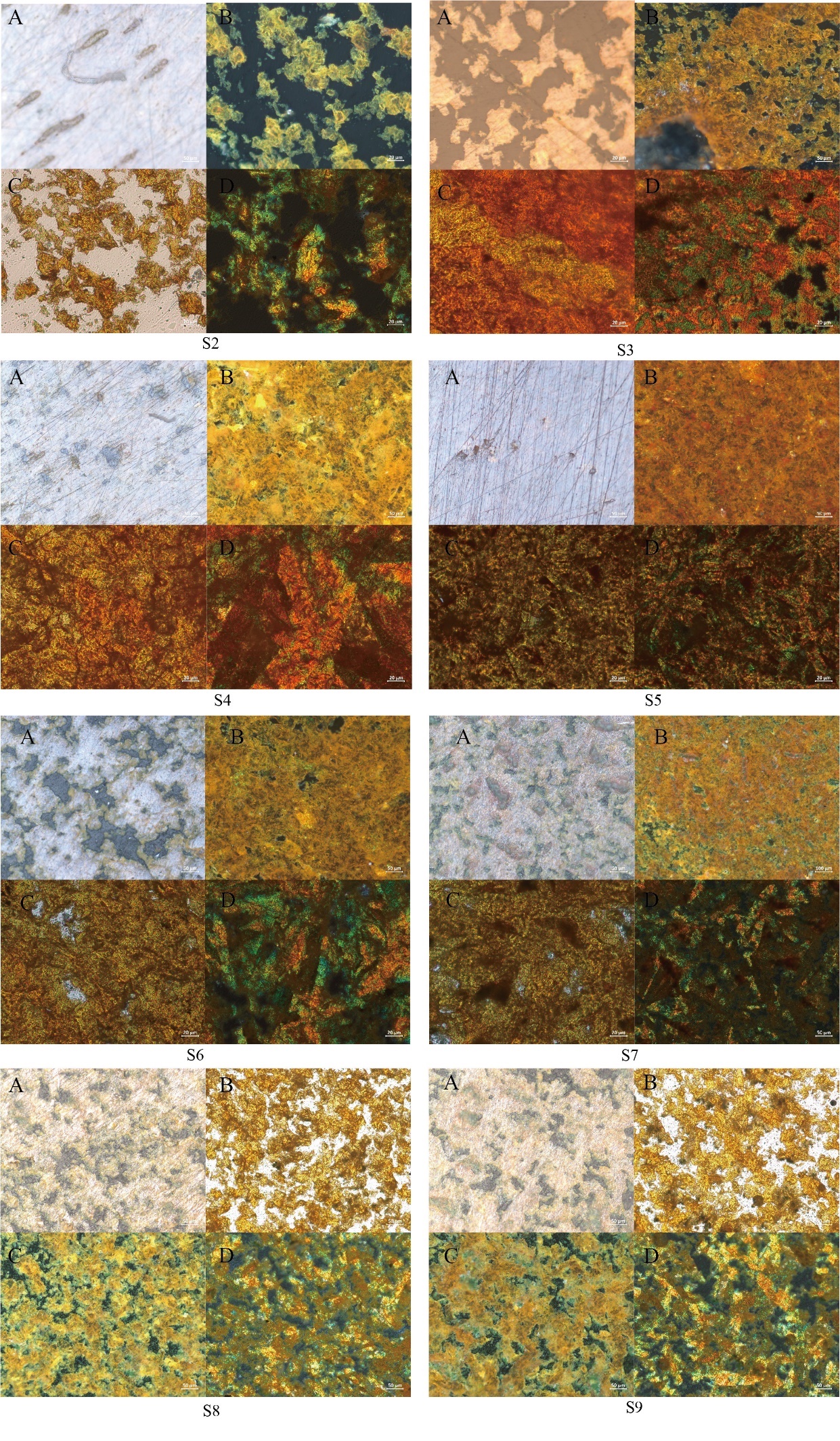


Supplement Figure 3. The microscopic characteristics of natural realgar and artificially optimized realgar.
